# Dynamics of extended-spectrum cephalosporin resistance genes in *Escherichia coli* from Europe and North America

**DOI:** 10.1038/s41467-022-34970-7

**Published:** 2022-12-12

**Authors:** Roxana Zamudio, Patrick Boerlin, Racha Beyrouthy, Jean-Yves Madec, Stefan Schwarz, Michael R. Mulvey, George G. Zhanel, Ashley Cormier, Gabhan Chalmers, Richard Bonnet, Marisa Haenni, Inga Eichhorn, Heike Kaspar, Raquel Garcia-Fierro, James L. N. Wood, Alison E. Mather

**Affiliations:** 1 Quadram Institute Bioscience, Norwich Research Park, Norwich, NR4 7UQ UK; 2 Department of Pathobiology, University of Guelph, Guelph, N1G 2W1 Canada; 3 Microbes Intestin Inflammation et Susceptibilité de l’Hôte (M2ISH), Faculté de Médecine, Université Clermont Auvergne, Clermont-Ferrand, 63001 France; 4 Centre National de Référence de la résistance aux antibiotiques, Centre Hospitalier Universitaire de Clermont-Ferrand, Clermont-Ferrand, 63000 France; 5 Unité Antibiorésistance et Virulence Bactériennes, Anses Laboratoire de Lyon, Université de Lyon, Lyon, 69007 France; 6 Institute of Microbiology and Epizootics, Department of Veterinary Medicine, Freie Universität Berlin, Berlin, 14163 Germany; 7 Veterinary Centre for Resistance Research (TZR), Department of Veterinary Medicine, Freie Universität Berlin, Berlin, 14163 Germany; 8 National Microbiology Laboratory, Public Health Agency of Canada, Winnipeg, Manitoba R3E 3R2 Canada; 9 Department of Medical Microbiology and Infectious Diseases, Max Rady College of Medicine, Rady Faculty of Health Sciences, University of Manitoba, Winnipeg, Manitoba R3E 0J9 Canada; 10 Department Method Standardisation, Resistance to Antibiotics Unit Monitoring of Resistance to Antibiotics, Federal Office of Consumer Protection and Food Safety, Berlin, 12277 Germany; 11 Department of Veterinary Medicine, University of Cambridge, Cambridge, CB3 0ES UK; 12 University of East Anglia, Norwich, NR4 7TJ UK

**Keywords:** Antimicrobial resistance, Phylogeny, Bacterial genetics, Bacterial genomics

## Abstract

Extended-spectrum cephalosporins (ESCs) are critically important antimicrobial agents for human and veterinary medicine. ESC resistance (ESC-R) genes have spread worldwide through plasmids and clonal expansion, yet the distribution and dynamics of ESC-R genes in different ecological compartments are poorly understood. Here we use whole genome sequence data of Enterobacterales isolates of human and animal origin from Europe and North America and identify contrasting temporal dynamics. AmpC β-lactamases were initially more dominant in North America in humans and farm animals, only later emerging in Europe. In contrast, specific extended-spectrum β-lactamases (ESBLs) were initially common in animals from Europe and later emerged in North America. This study identifies differences in the relative importance of plasmids and clonal expansion across different compartments for the spread of different ESC-R genes. Understanding the mechanisms of transmission will be critical in the design of interventions to reduce the spread of antimicrobial resistance.

## Abstract (web summary)

Extended-spectrum cephalosporin resistance genes in *Escherichia coli* have spread worldwide. Here, the authors dissect the emergence and distribution of these genes over time, and across geographic location and host species, to better understand their dynamics and mechanisms of transmission.

## Introduction

Third- and fourth-generation cephalosporins are broad-spectrum β-lactam antimicrobial agents classified as critically important for human medicine by the World Health Organization due to their use in the management and treatment of serious infections caused by multidrug resistant (MDR) Gram-negative and Gram-positive bacteria^1,2^. Third-generation cephalosporins were introduced for medical use in the early 1980s, with fourth-generation cephalosporins introduced in 1994^3^; since then, resistance to these drugs has increased^4^. Resistance to these extended-spectrum cephalosporins (ESCs) is widespread worldwide in Enterobacterales from both humans and animals, and is mediated predominantly by two main groups of inactivating enzymes: extended-spectrum beta-lactamases (ESBLs) and plasmid-mediated AmpC β-lactamases (AmpCs)^5,6^.

There are differences in the distribution of ESC resistance (ESC-R) genes between host species and geographical origins^6^. ESBLs were initially detected in Europe; in 1983, the first ESBL—a SHV variant—was identified in Germany in *Klebsiella pneumoniae* and *Serratia marcescens* isolates^7^. Until the end of the 1990s, most of the ESBLs detected were variants of SHV and TEM^8^. Since then, the main types in Europe have changed, with CTX-M β-lactamases becoming the dominant ESC-R enzymes in Enterobacterales in humans and farm animals^9^. For example, the ESBL gene *bla*_CTX-M-1_ is broadly disseminated among *Escherichia coli* from animals in Europe^6^. In human patients, the frequency of ESBL-producing *E. coli* and *K. pneumoniae* isolates was higher in Europe compared to North America between 2004 and 2006^8,10^. In Canada, a national study reported the AmpC gene *bla*_CMY-2_ in *E. coli* isolates from patients of tertiary-care hospitals in 2000^11^. Subsequently, this variant was also reported in *E. coli* isolates from intensive care units (ICUs) between 2005 and 2006^12^. The first reports of *bla*_CMY-2_ in Enterobacterales from food-producing animals in Canada were between 1995 and 1999^13^, and the gene has been commonly found in *E. coli* and *Salmonella enterica* from food-producing animals and food since then^14–16^. ESBLs in Canada were first observed in 2000 in *E. coli* and *Klebsiella* spp. from human clinical samples^17^. More recently, there have been reports of increasing occurrence of CTX-M-type β-lactamases in Enterobacterales in farm and companion animals from Canada^18,19^. In contrast, *bla*_CMY-2_ was rarely reported in *E. coli* from humans in Europe^20,21^, but *bla*_CMY-2_ isolates from livestock production have been reported^6^. A recent study from Germany found *bla*_CMY-2_ in isolates from humans, livestock and meat products^22^, and it has also been reported in *E. coli* from companion animals in France^23^. International travel and trade have likely influenced the global dissemination of ESC-R genes^24^. Comparison among ESBL and AmpC-producing *E. coli* isolates of human and animal origins from Germany, the Netherlands and the United Kingdom showed that human isolates were more closely related to each other than to isolates from animals^25^. In another recent study in The Netherlands, the majority of community-acquired ESBL and AmpC carriage among *E. coli* was found to be due to human-to-human transmission, with much less attributed to food, animal and environmental sources^26^.

ESBL and AmpC genes are typically located on mobile genetic elements^27^ and their spread has been linked to horizontal gene transfer via plasmids^28–30^. However, the expansion of bacterial clones carrying ESC-R genes also plays a role^31^. For example, the global spread of *bla*_CTX-M-15_ in humans is associated with the *E. coli* epidemic lineage sequence type (ST) 131^31,32^. Although numerous studies have been conducted examining the types and putative sources of ESC-R genes^9–16,19,21,27^, these mostly investigated isolates from single host species or geographical regions. To gain a broader understanding of the emergence and spread of resistance to these important drugs, diverse sources must be examined. In addition, the relative importance of horizontal gene transfer and clonal expansion in the spread of ESC-R between ecological niches and biological compartments is unclear but may shed light on where to target interventions to reduce the burden of antimicrobial resistance (AMR). In this study, we used genomic and evolutionary approaches with an extensive collection of Enterobacterales isolates to determine the dynamics and evolution of ESBL and AmpC over time in different ecological compartments (country and source) and identify potential factors contributing to the spread and persistence of ESC resistance. Thus we (i) identified similarities and differences in ESC-R genes between multiple Enterobacterales species, host species and geographical regions; (ii) analysed clonal lineages and putative plasmids able to spread across compartments; and (iii) examined the temporal trend of ESC-R genes in different compartments.

## Results

### Bacterial isolates and epidemiological data

A total of 1930 genomes were used in this study (Supplementary Table S1) for which extensive epidemiological metadata were available. These included *E. coli*, *K. pneumoniae*, *S. enterica*, *Enterobacter hormaechei*, *Klebsiella oxytoca*, *Proteus mirabilis*, *Klebsiella michiganensis*, *Citrobacter europaeus*, *Citrobacter freundii* and *Raoultella ornithinolytica* (Supplementary Table S2). Of the 1818 *E. coli*, 1617 were ESC-R isolates collected between 2008 and 2016, 79 ESC-R isolates before 2008 or after 2016 and 122 ESC-susceptible isolates mainly from 2008 to 2016. The set also included 112 non-*E. coli*, comprising 108 ESC-R genomes from 2008 to 2016, and four ESC-susceptible non-*E. coli* (Supplementary Figs. S1–S3).

The isolates derived from diverse sources, which were classified into eleven categories: human, cattle, chicken, food (porcine, bovine and avian meat), pig, turkey, dog, horse, wastewater (including wastewater from human activity as well as from animal and meat processing plants), cat and other avian sources (bird and duck) (Supplementary Table S3 and Source Data file). Overall, the majority of isolates were of human (*n* = 817, 42.3%), cattle (*n* = 372, 19.3%) and chicken (*n* = 263, 13.6%) origins (Supplementary Fig. S4). Assessment of the potential inclusion of outbreak-associated isolates, which may introduce bias into the analyses, identified only two ST10 and six ST177 genomes sharing ≤10 single nucleotide polymorphisms (SNPs) in their core genome. For ST131, there were only 36 genomes (mostly with *bla*_CTX-M-15_) sharing ≤10 SNPs, but these were from different years (from 2008 to 2016) and countries. Overall, this suggests outbreak isolates were not included in this study.

### MLST and plasmid replicon identification

Assembly summary statistics are presented in the Source Data file. There were 285 known STs and 13 unknown STs identified among the 1818 *E. coli* isolates, the most common being ST131 at 19.7% (*n* = 359), ST10 at 6.9% (*n* = 125) and ST117 at 5.6% (*n* = 102) (Supplementary Fig. S5A). These three common STs were identified in all four countries (Canada, France, Germany and the UK) (Supplementary Fig. S5B) and multiple host species, with ST131 mainly associated with humans, ST10 mainly with cattle and ST117 mainly with poultry (Supplementary Fig. S5C–F). The distribution of isolates showed relatively even coverage of the main study time period between 2008 and 2016 for Canada, France and Germany. Relatively higher numbers of isolates were included in specific years for the UK (2015) and Germany (2012), but this was due to the sample selection in the previously published studies^22,32,33^ (Supplementary Figs. S6A, B, S7A–D). Regarding identification of putative plasmids, 60 replicons were identified in the *E. coli* isolates, representing 19 plasmid incompatibility (Inc) types (Supplementary Table S4, Supplementary Figs. S8, S9).

### Genes conferring resistance to third generation cephalosporins

By study design, the majority of isolates selected for inclusion were phenotypically resistant to ESCs and so do not represent the prevalence of ESC-R in the general populations. Among the 1696 ESC-resistant *E. coli* isolates, 39 different ESC-R genes were identified. There were 55 combinations, or profiles, of ESC-R genes, some of which comprised more than one ESC-R gene. The most frequent types and profiles of ESC-R genes were: *bla*_CTX-M-1_ (*n* = 414) and *bla*_CTX-M-15_ (*n* = 418) with 24.4% and 24.6%, respectively, followed by *bla*_CMY-2_ 19.2% (*n* = 326), *bla*_CTX-M-14_ 10.8% (*n* = 183), *bla*_SHV-12_ 8.3% (*n* = 140), *bla*_CTX-M-27_ 3.5% (*n* = 60), *bla*_CTX-M-55_ 1.2% (*n* = 20), *bla*_CTX-M-2_ 0.9% (*n* = 16), and *bla*_CMY-2_ + *bla*_SHV-12_ 0.7% (*n* = 12). The remaining 46 ESC-R profiles were rarely observed, each with 10 or fewer isolates. The non-*E. coli* isolates shared 11 ESC-R profiles with *E. coli*, while there were 44 ESC-R profiles unique to *E. coli* and 16 ESC-R profiles unique to the non-*E. coli* isolates (Supplementary Fig. S10A, B).

**Fig. 1:**
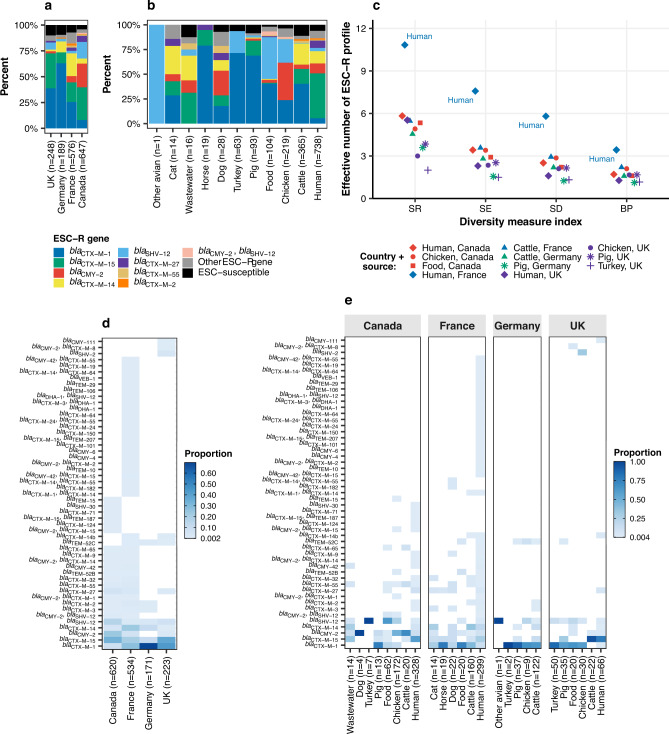
Frequency and diversity of ESC-R genes in *E. coli* by country and source. **a** Percentage of ESC-R gene by country and **b** source (total *n* = 1,660 ESC-R + ESC-susceptible genomes*). The bars are coloured by ESC-R gene, as shown in the inset legend. **c** Diversity indices (SR: species richness; SE Shannon entropy, SD Simpson diversity, and reciprocal BP Berger-Parker) of ESC-R profile by compartment (country + source), where only compartments with minimum sample size of *n* = 30 were included in the analysis (total *n* = 1361 ESC-R genomes*). The shape of the dots is linked with the source and the colours with the country. **d** Common and unique ESC-R genes among countries and **e** compartments (total *n* = 1548 ESC-R genomes*). Heatmaps are coloured as per inset scale bars. *Genomes from Pietsch et al.^22^ (*n* = 158; 148 ESC-R genomes + 10 ESC-susceptible genomes) were excluded. Source data are available in the Source Data file. ESC-R: extended-spectrum cephalosporin resistance.

The major ESC-R genes in *E. coli*, *bla*_CTX-M-1_, *bla*_CTX-M-15,_ *bla*_CMY-2_, *bla*_CTX-M-14_ and *bla*_SHV-12_, were globally distributed, though differences in frequency were found between countries, sources and compartments (Fig. 1a, b, Supplementary Fig. S11 and Supplementary Tables S5, S6). To note, this analysis did not include the isolates from Pietsch et al.^22^ (*n* = 148 ESC-R genomes) as the design of that study focused specifically on German *bla*_CMY-2_ isolates; after excluding these 148 genomes, this resulted in 1548 ESC-R *E. coli* genomes which were used for further comparative analysis. In this collection of 1548 ESC-R *E. coli* genomes, the *bla*_CTX-M-1_ gene was more frequent in European countries (France, Germany and the UK), while *bla*_CTX-M-15_ and *bla*_CMY-2_ were more common in North America (Canada) (Fisher’s Exact test, adjusted *p*-value < 0.0001; Fig. 1a)_._ The *bla*_CTX-M-1_ gene was more frequent in isolates of animal (cattle, chicken, pig, turkey, dog, horse and cat) than of human origin, whereas *bla*_CTX-M-15_ was more frequent in human isolates (Fisher’s Exact test, adjusted *p*-value < 0.0001). The frequency of *bla*_CMY-2_ was similar between isolates from humans and animals (Fisher’s Exact test, adjusted *p*-value = 0.1) (Fig. 1b).

Fifteen ESC-R profiles were observed at least in two countries (Fig. 1d). Six profiles were found only in Canada, in human (*n* = 5), chicken (*n* = 1) and cattle (*n* = 1) isolates. Three profiles were unique to UK isolates from human (*n* = 1), food (*n* = 1) and chicken (*n* = 10) sources (Fig. 1e). Twenty-three ESC-R profiles were unique to France, mainly in human isolates (Fig. 1e). Highly diverse ESC-R profiles were observed in isolates of French human origin (Fig. 1c), which was not the case for the ST and plasmid replicon profiles (Supplementary Fig. S12A, B).

### Location of ESC-R genes in plasmid and chromosome contigs

Ascertainment of ESC-R gene locations in plasmids or chromosomes was obtained through MOB-suite^34^ and RFPlasmid^35^ (Supplementary Table S7). Initial use of MOB-suite alone identified an unexpectedly high proportion of ESC-R genes on chromosome-classified contigs, particularly for the *bla*_CTX-M-15_ and *bla*_SHV-12_ genes. Plasmid-classified contigs with ESC-R genes were further categorised into typeable plasmids (presence of replicon sequences in the same contig) and non-typeable plasmids (no identified replicon sequences in the same contig). In contrast, application of the RFPlasmid tool classified the majority of all ESC-R genes examined as located on plasmids, the majority of which were non-typeable plasmids. To resolve these disagreements and validate the location of ESC-R genes, we randomly selected 20 isolates—five each with the four most common ESC-R genes identified, *bla*_CTX-M-1_, *bla*_CTX-M-15,_ *bla*_CMY-2_ and *bla*_CTX-M-14_—where prediction of the genomic location of the ESC-R gene differed between MOB-suite and RFPlasmid and performed long-read sequencing to obtain complete contiguated genome sequences. In all 20 long-read genome sequences, the RFPlasmid predictions were correct, suggesting MOB-Suite over-predicted the number of chromosome-located ESC-R genes. Using the RFPlasmid results (Supplementary Table S7), the majority of each of the five main ESC-R genes were located on plasmids, but to different degrees. The vast majority of *bla*_CMY-2_ genes were predicted to be plasmid-borne (95.7%), in contrast to *bla*_CTX-M-14_ (78.1%) and *bla*_CTX-M-15_ (79.9%). Overall, 313 typeable plasmids linked with ESC-R genes (hereafter ESC-R plasmids) were recovered from the short-read data (*bla*_CTX-M-1_ *n* = 136, *bla*_CTX-M-15_ *n* = 23, *bla*_CMY-2_ *n* = 88, *bla*_CTX-M-14_ *n* = 40 and *bla*_SHV-12_ *n* = 26), representing 27.6% of the predicted plasmids associated with ESC-R genes. Of these, IncI1 and IncF were the most frequently identified plasmid Inc types associated with ESC-R genes. *bla*_CTX-M-1_ and *bla*_CMY-2_ were predominantly associated with IncI1 plasmids, *bla*_CTX-M-15_ and *bla*_SHV-12_ with IncF plasmids, and *bla*_CTX-M-14_ with both IncI1 and IncF plasmids (Supplementary Table S8). Analysis of the entire genetic content (plasmidome) of the 313 typeable and a subset of 79 non-typeable ESC-R-carrying plasmid contigs showed clustering by ESC-R genes, plasmid replicon types and subtypes (Supplementary Fig. S13).

### Inferring the mechanisms of transmission and fitness of main ESC-R genes in *E. coli*

Simpson’s diversity indices of STs and plasmid replicon profiles in the subset (*n* = 1343) of the genomes carrying at least one of the five major ESC-R genes were calculated and compared; we refer to this as the ‘main ESC-R gene’ dataset hereafter. This analysis was also performed on the subset of 313 typeable ESC-R plasmid contigs as a validation step (Table 1). The ST diversity indices for most ESC-R genes were similar, ranging from 17.94 to 33.90 in the main ESC-R gene dataset and from 10.46 to 23.79 in the typeable plasmid subset. The exception was for genomes with *bla*_CTX-M-15_, where the ST diversity was 3.24 in the main ESC-R gene dataset and 3.66 in the typeable plasmid subset. The plasmid replicon profile diversity for genomes carrying most ESC-R genes were also similar in the main ESC-R gene dataset, with indices ranging from 72.88 to 81.88; the exception was genomes with *bla*_CMY-2_, where the index was 145.71. In the typeable plasmid subset, the range of plasmid replicon profile diversity was lower, between 1.20 and 5.83. To infer the relative importance of clonal expansion versus putative plasmids for dissemination of ESC-R genes in *E. coli*, the ratio of plasmid-to-ST diversities for each major ESC-R gene was calculated. For *bla*_CTX-M-1_, *bla*_CTX-M-14_ and *bla*_SHV-12_ in the main ESC-R gene dataset, the ratio was slightly <3 (range 2.42–2.88); for *bla*_CMY-2_ the ratio was 8.12, and for *bla*_CTX-M-15_ the ratio was 22.49. In the typeable plasmid subset, the ratio was 0.05 and 0.10 for *bla*_CTX-M-1_ and *bla*_CTX-M-14_, respectively, 0.17 for *bla*_CMY-2_, 0.56 for *bla*_SHV-12_ and 1.59 for *bla*_CTX-M-15_. Plasmid replicon profile and ST Simpson’s diversity indices were also calculated for each major ESC-R gene by individual compartment in the full dataset (Supplementary Fig. S14).

**Table 1:** Characteristics and Simpson’s diversity (SD) of sequence types (STs) and plasmid replicon profiles of *E. coli* (*n* = 1,343) and *E. coli* with typeable ESC-R gene-carrying plasmids (*n* = 313) harbouring the five major ESC-R genes

	Main ESC-R gene genome dataset				Typeable ESC-R plasmids subset				
Main ESC-R gene	# of isolates (% of total 1,343)^a^	SD of STs (95% CI)^a^	SD of plasmid replicon profiles (95% CI)^a^	Ratio of plasmid/ST diversity^a^	# of typeable plasmids^b^	SD of STs (95% CI)^b^	SD of plasmid replicon profiles (95% CI)^b^	Ratio of plasmid /ST diversity^b^	Inference of transmission^b^
*bla*_CTX-M-1_	414 (30.8%)	29.35 (27.53–34.39)	79.04 (66.64–99.11)	2.69	136	21.86 (19.07–29.03)	1.20 (1.20–1.32)	0.05	Dominant plasmid [IncI1 pST3]
*bla*_CTX-M-15_	418 (31.1%)	3.24 (3.23–3.74)	72.88 (62.31– 94.27)	22.49	23	3.66 (3.37–6.68)	5.81 (4.94–8.31)	1.59	Dominant ST [ST131]
*bla*_CMY-2_	188 (14.0%)	17.94 (16.53–23.90)	145.71 (93.26–198.16)	8.12	88	18.12 (15.3–26.14)	3.10 (3.05–3.74)	0.17	Diverse plasmids
*bla*_CTX-M-14_	183 (13.6%)	25.56 (22.64–30.89)	73.61 (52.91–106.33)	2.88	40	23.79 (15.39–35.63)	2.37 (2.32–2.81)	0.10	Dominant plasmid [IncF F2:A-:B-]
*bla*_SHV-12_	140 (10.4%)	33.90 (27.61–43.16)	81.88 (52.13–112.92)	2.42	26	10.46 (7.86–22.18)	5.83 (5.05–8.91)	0.56	Diverse plasmids in a common ST [ST117]

^a^Numbers and diversity analysis obtained from 1343 *E. coli* genomes carrying the main five ESC-R genes from short-read data.

^b^Numbers and diversity analysis obtained from 313 typeable ESC-R plasmids.

### Trends over time of ESC-R genes by compartment in *E. coli*

**Fig. 2:**
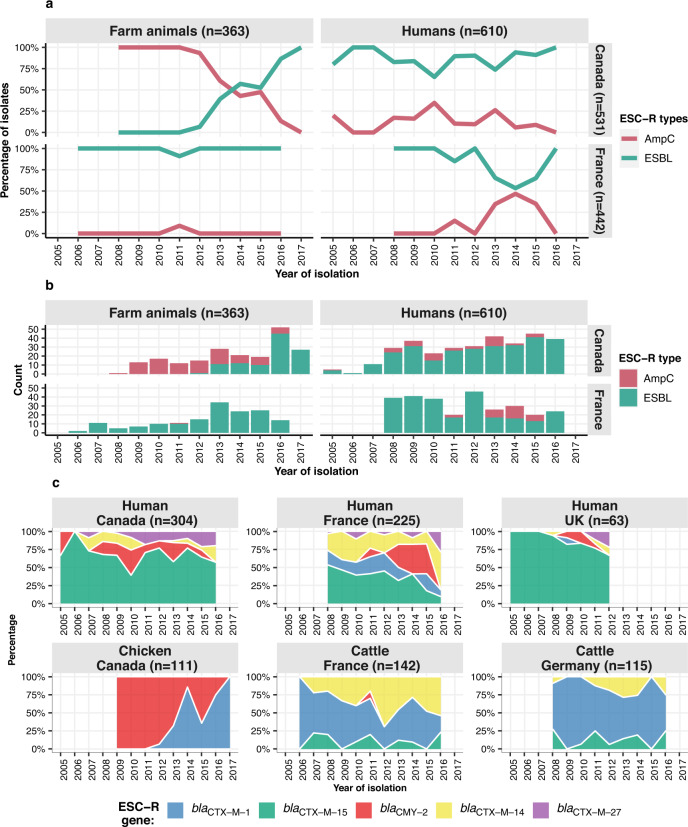
Distribution and trend over time of ESC-R genes in *E. coli* by compartment. **a** Trends in percentage and **b** count for ESC-R types over time in Canada and France across humans and farm animals (cattle, chickens, pigs and turkeys). The lines and bars are coloured by ESC-R types. **c** Trends over time of the percentage of the major ESC-R gene in main compartments (colour key is in inset legend). Source data are available in the Source Data file. ESC-R extended-spectrum cephalosporin resistance, ESBL extended-spectrum β-lactamases.

In the two countries from which both human and animal data were available, the dynamics of AmpCs and ESBLs changed over the study period (Fig. 2a, b). In farm animals (mainly chickens) in Canada, the main ESC-R gene type shifted from AmpC to ESBL (Wald z-statistic, *p* < 0.0001). Conversely, in humans from France, the proportion of ESBLs declined over time while that of AmpCs increased (Wald z-statistic, *p* < 0.0001); nevertheless, ESBLs remained the most common gene type (Supplementary Table S9). There were no significant changes in AmpC and ESBL frequency over time in the other examined compartments, humans from Canada (Wald z-statistic, *p* = 0.2), nor in farm and companion animals from France (Wald z-statistic, *p* = 1.0) (Supplementary Table S9). When evaluated by individual ESC-R genes, contrasting trends within individual compartments were observed. For example, over the study period *bla*_CMY-2_ increased in human isolates from France, while this ESC-R gene decreased in chickens from Canada and was replaced by *bla*_CTX-M-1_ (Fig. 2c, Supplementary Table S9).

### Resistance to other antimicrobial classes

**Fig. 3:**
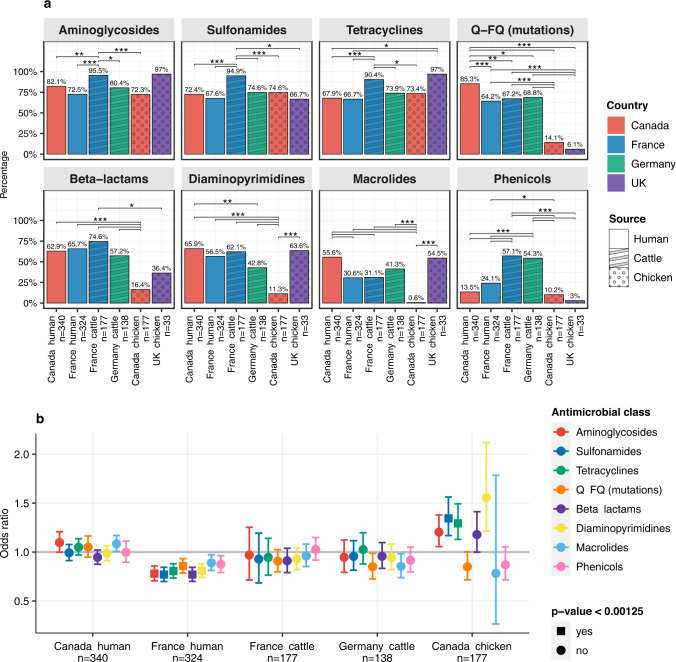
Presence and dynamics of AMR determinants in *E. coli*. **a** Presence of resistance determinants by antimicrobial class and compartment (country + source) for *n* = 1189 *E. coli* isolates. Two-sided Fisher’s Exact Test was used to compare the proportion of AMR class between compartments, and the significance levels are indicated with asterisks: *p < 0.01, **p < 0.001 and ***p < 0.0001. The bars are coloured by country and the pattern by source. **b** Odds ratios and 95% confidence intervals (coloured as per inset legend) demonstrating change in AMR determinant presence over the study period by compartment; significance cut-off is 0.00125 after Bonferroni adjustment for multiple hypothesis testing. Source data are available in the Source Data file. Q-FQ quinolone-fluoroquinolone.

In addition to the ESC-R genes, an additional 137 acquired AMR genes conferring resistance to other antimicrobial agents were identified, with up to 22 AMR genes and up to 12 plasmid replicons in a single genome (Supplementary Figs. S15, S16). There was variable presence of determinants (genes or SNPs) conferring resistance to different antimicrobial classes between compartments (Fig. 3a, Supplementary Tables S10, S11). Logistic regression analysis identified that determinants conferring resistance to aminoglycosides, sulfonamides, tetracycline, beta-lactams, diaminopyrimidines and quinolones-fluoroquinolones (due to mutations in *gyrA*, *parC* and *parE*; Supplementary Fig. S17 and Table S12) decreased over time in human isolates from France (Wald z-statistic, *p*-value < 0.00125), while in chickens from Canada the presence of genes conferring resistance to sulfonamides and tetracycline increased over time (Wald z-statistic, *p*-value < 0.00125) (Fig. 3b). Diversity indices showed highly diverse AMR profiles in isolates of French human origin and animals from France and Germany (Supplementary Fig. S18).

### Core-gene SNP-based phylogeny for *E. coli*

**Fig. 4:**
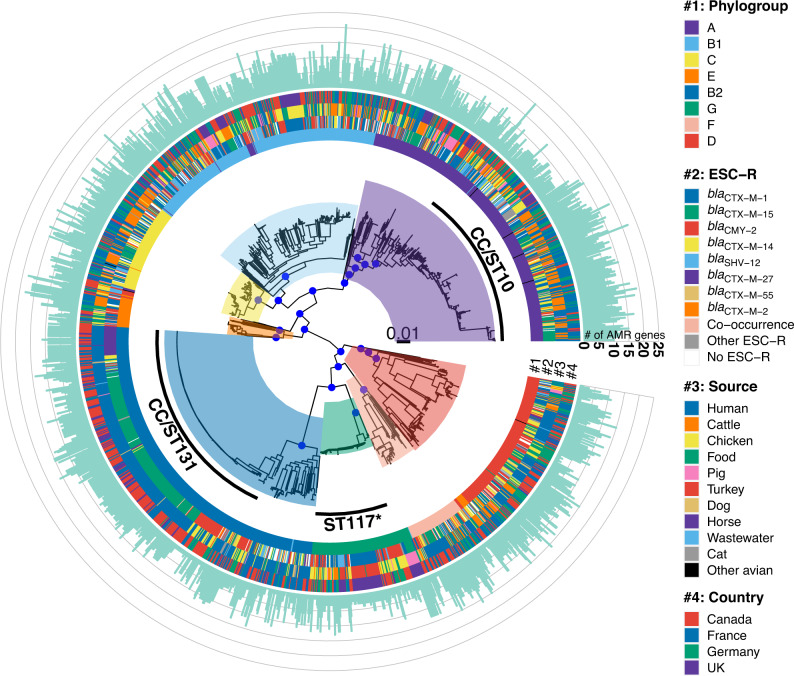
Core-gene phylogenetic tree for 1818 *E. coli* isolates. Maximum likelihood tree with eight main clusters linked with phylogroup. Bootstrap support values > 95 are represented by blue dots for main clusters. Metadata are represented next to the tree by four rings: phylogroup, ESC-R gene, source and country (coloured as per inset legend). The number of AMR genes per genome is plotted on the outside as a bar plot (light green colour).*ST117 cluster contains mainly ST117 but also other closely related STs. Source data are available in the Source Data file. ESC-R extended-spectrum cephalosporin resistance.

A maximum likelihood core-gene tree was constructed for 1818 *E. coli* genomes (*n* = 1696 ESC-R and *n* = 122 ESC-susceptible). The length of the core-gene alignment was 2300,993 bp (with 365,784 SNPs), which represents 45% of the reference genome EC958 (Refseq: NZ_HG941718.1). In the phylogenetic tree, there were eight major clusters largely concordant with phylogroup identification^36^ (Fig. 4). Within phylogroup A, CC/ST10 was predominant, in phylogroup G the main ST was ST117 and in phylogroup B2 it was CC/ST131 (Fig. 4). These results were consistent with the pangenome network analysis, where clusters largely matched the population structure of the core-gene tree (Supplementary Fig. S19). There were some exceptions, particularly with phylogroup D genomes, that were phylogenetically clustered in the core-gene tree but fell into distinct clusters in the gene content network (Supplementary Fig. S19A).

Genomes from different countries and sources were distributed across the different phylogroups, with ESC-susceptible genomes placed in diverse clusters. Previously identified host associations were observed, such as ST131 with humans and ST117 with chickens. Similarly, the ESC-R genes were widely distributed in the different clusters, with the exception of the previously identified association of *bla*_CTX-M-15_ with ST131. Separate phylogenetic trees were constructed for genomes carrying *bla*_CTX-M-1_, *bla*_CTX-M-15_, *bla*_CMY-2_, *bla*_CTX-M-14_, *bla*_SHV-12_, *bla*_CTX-M-27_, *bla*_CTX-M-55_ or *bla*_CTX-M-2_ (Supplementary Figs. S20–S22). Overall, the main clusters displayed in each tree correlated with phylogroups.

The 360 CC/ST131 genomes were classified in five clades based on marker alleles for the type 1 fimbriae *fimH* (H) adhesin: clade A (H41, *n* = 13) with half of the isolates carrying *bla*_CTX-M-14_, the remaining carrying other ESC-R genes; clade B (H22, *n* = 47; H30, *n* = 2; H54, *n* = 1) mostly carrying *bla*_CMY-2_; clade B0 (H27, *n* = 1) with a single *bla*_SHV-12_ isolate; clade C1, also known as *H*30-R (H30, *n* = 70), with more than half of isolates carrying *bla*_CTX-M-27_; and clade C2, also known as H30-Rx (H30, *n* = 220; H35, *n* = 6), mostly harbouring *bla*_CTX-M-15_. Similar results were previously reported^22,32,37–41^. All genomes in Clades C1 and C2 also had double mutations in the *gyrA* and *parC* genes, which are associated with high-level resistance to fluoroquinolones^42^.

## Discussion

Resistance to ESC drugs is a global public health concern due to its widespread nature and the use of these drugs for difficult to treat infections. Detailed within-country investigations have been conducted, which have identified patterns of transmission between host species. For example, in The Netherlands ESBL/AmpC gene profile distribution from livestock and food-associated *E. coli* were different from those in *E. coli* from humans^43^, and a further study revealed humans as the main source of community-acquired ESBL and AmpC carriage in *E. coli* from humans based on distinguishable patterns of ESBL and AmpC gene occurrence in different sources^26^. Similarly, in the UK, whole genome sequencing (WGS) demonstrated distinct populations of *E. coli*, including those with ESBL and AmpC genes, in livestock and retail meat compared with those isolated from bloodstream infections in humans^33^. However, given the global nature of food supplies, animal movement and human travel, examining patterns from multiple countries and sources can provide additional insight and comparison of patterns and transmission of AMR. In particular, the use of WGS applied in this context provides valuable information for monitoring trends of specific ESBL and AmpC genes, as well as other AMR genotypes and potential linkage to particular plasmids, as was recently demonstrated in a collection of pig isolates from the UK^44^ and in calf isolates from France^45^. Here, a genomic and evolutionary approach was used to determine the distribution and dynamics over time of ESBL and AmpC in *E. coli* from different geographical origins and sources and to infer the mechanisms underlying the transmission of the major ESC-R genes.

In comparing Canada and France, countries for which both human and animal data were available, two trends are clear. First, in farm animals in Canada (represented predominantly by chickens in this dataset), the majority of isolates carried AmpC genes at the beginning of the study period, but by the end, the majority of ESC-R was due to ESBL genes. In contrast, the main determinant of ESC-R in French farm animals (represented predominantly by cattle in this dataset) consistently was ESBL genes. Second, in humans from Canada both AmpC and ESBL genes were found over the entire study period, with ESBL genes consistently found at a higher prevalence. In humans from France, ESC-R was initially dominated by ESBL genes, and then part way through the study period the AmpC gene *bla*_CMY-2_ was identified and increased over time, although ESBLs remained as the main resistance mechanism. These observations agree with previous work describing the emergence and increase of ESBLs in Europe^8,9,46^, where *bla*_CTX-M_ variants have become major determinants in humans and animals in different enteric bacterial species^9^, with later emergence of *bla*_CMY-2_^22^. The results presented are also consistent with previous work from North America, where the AmpC gene *bla*_CMY-2_ was identified in isolates from humans, animals and foods in the early to mid 2000s^12,14^, while more recent studies have detected the presence of *bla*_CTX-M_-type variants in animals from Canada^19^.

The major ESC-R genes in *E. coli* identified in this study were *bla*_CTX-M-1_, *bla*_CTX-M-15_, *bla*_CMY-2_, *bla*_CTX-M-14_ and *bla*_SHV-12_. These genes are also found in other enteric bacterial species, like those included in this study, and across the globe^6^. Despite their ubiquitous nature, there was variation in their distribution and frequency between geographical origins and sources. In these data, *bla*_CTX-M-1_ was more frequent in Europe compared to North America, where *bla*_CMY-2_ and bla_CTX-M-15_ were more common. With respect to source (host species), *bla*_CTX-M-1_ was more frequent in animals, *bla*_CTX-M-15_ in humans, and *bla*_CMY-2_ was frequent in both humans and animals. The link between specific sources and these ESC-R genes has been previously reported^6,22,32,33^. ESC-susceptible genomes were distributed throughout different clusters and compartments (country + source), thereby indicating that, in general and with the exceptions mentioned, ESC-R genes were not associated with particular chromosomal backgrounds or ecological compartments.

The first report of *bla*_CTX-M-15_ identified its location on large plasmids isolated from *E. coli*, *K. pneumoniae* and *E. aerogenes* isolates from urinary and pulmonary specimens of patients hospitalized in New Delhi, India, in 1999^47^. From August 2000 to April 2002 in Canada, a major outbreak of multidrug-resistant and ESBL-producing *E. coli* isolates was identified in long-term care facilities^48^. A following study identified a plasmid with the *bla*_CTX-M-15_ gene associated with the outbreak strain, and this plasmid was genetically very similar to the one isolated in India^49^. In the UK, *bla*_CTX-M-15_ isolates were initially isolated from clinical samples in 2001^50^; subsequently, the spread of *bla*_CTX-M-15_ was associated with the expansion of the human-associated clone ST131^32^. In this study, a higher proportion of *bla*_CTX-M-15_ was identified in Canada and the UK compared to France, related to the higher proportion of ST131 in these countries. In France, more diverse STs were associated with *bla*_CTX-M-15_, suggesting plasmids—particularly IncF plasmids—may play a role in the dissemination of this gene in that setting. Although ST131 and, therefore, *bla*_CTX-M-15_ was strongly associated with human-derived isolates, it was also observed in isolates of dog, pig and wastewater origin. Transmission between humans and pets has been observed previously^6,51^. In addition to the *bla*_CTX-M-15_/ST131/human association, *bla*_CTX-M-27_ was also observed to occur mainly in ST131 clade C1 human isolates, again with multiple IncF replicons. In 2006, ST131 C1/*H*30R with *bla*_CTX-M-27_ was detected in Japan^52^, followed by a reported rise of *bla*_CTX-M-27_^46^. The expansion of the ST131 C1/*H*30R clade was found to be responsible for the increase of this ESBL gene^40^, and was subsequently observed in France^53^ and Germany^54^. Moreover, our study also found *bla*_CMY-2_ predominantly in ST131 clade B in humans and chickens from Canada, an association previously reported elsewhere^22,41^.

The AmpC gene *bla*_CMY-2_ has been reported frequently in North America and to be increasing in Europe^22^. Here we confirm those results in our temporal analysis of genomes from France, from which both human- and animal-derived genomes are available. We also revealed that this significant increase was observed only among isolates from humans and not from farm animals (here, predominantly cattle). We observed similar frequencies between humans from Canada and France, but temporal trend analysis showed the frequency increased over time in French human isolates. In contrast to the relatively constant presence of the gene in Canadian human-derived genomes, *bla*_CMY-2_ decreased over time in Canadian chickens, and was replaced by *bla*_CTX-M-1_. IncA/C plasmids have previously been associated with *bla*_CMY-2_ in isolates from cattle faeces and wastewater in Canada^55^; in this study, IncA/C2, IncB/O/K/Z and IncI1 replicons were observed frequently in *bla*_CMY-2_-carrying *E. coli* from chickens in Canada. In Germany, the spread of *bla*_CMY-2_ was attributed to transmission of IncI1 and IncK plasmids in isolates of human origin^22^. Here we observed that human French *bla*_CMY-2_-carrying isolates frequently harboured IncI1 replicons, which is consistent with a previous French study of isolates from companion animals^23^.

Recovery of fully contiguated plasmid sequences from short-read data is very challenging due to the frequent occurrence of repeat elements that impede assembly of complete plasmid or chromosome sequences^56^. Despite this limitation, diverse bioinformatic tools are available for plasmid detection, classification and reconstruction^34,57,58^, with a recent study identifying MOB-suite as the only tool to reconstruct correctly the majority of *E. coli* plasmids in a test set^56^. Using MOB-suite, we were able to recover typeable ESC-R plasmids, but it over-represented the proportion of ESC-R genes carried on the chromosome. This was particularly true for *bla*_CTX-M-15_ and *bla*_SHV-12_ genes, which may be because these genes are associated with IncF plasmids. IncF plasmids are highly diverse with multiple copies of insertion sequences (ISs)^59^, which will limit successful plasmid assembly. The chromosome overrepresentation was resolved with RFPlasmid^35^, which identified a much larger proportion of ESC-R genes carried on plasmids and was later confirmed by long-read sequence data of a subset of isolates. Through this plasmid prediction approach, we were able to infer the proportions of ESC-R genes located on plasmids and the chromosome: *bla*_CMY-2_, *bla*_SHV-12_ and *bla*_CTX-M-1_ were frequently located on plasmids with 95.7%, 93.6% and 85.4%, respectively, while the proportion of plasmid-borne *bla*_CTX-M-14_ and *bla*_CTX-M-15_ was 78.1% and 79.9%, respectively. A total of 313 typeable ESC-R plasmids were recovered by MOB-suite and RFPlasmid, representing 27.6% of all predicted plasmid contigs associated with ESC-R genes. Although relatively low, this percentage is comparable to that obtained in a recent study^56^; the remaining plasmid contigs were not typeable as they did not contain replicon sequences.

As part of this study, the predominant mode of transmission of each of the five main ESC-R genes was assessed using the diversities of bacterial backgrounds (STs), plasmid replicons and their ratio in two datasets: (1) in the main ESC-R gene dataset of 1343 *E. coli* ESC-R genomes carrying at least one of the five main ESC-R genes, and (2) in the subset of 313 genomes containing typeable ESC-R plasmids (Table 1). From the analysis of the main ESC-R gene dataset, two main trends were apparent. First, in four of the five ESC-R genes, ST diversity was similar; the exception was *bla*_CTX-M-15_, which had lower ST diversity. This was to be expected, given the predominance of ST131 representing the chromosomal backgrounds of isolates with the *bla*_CTX-M-15_ gene. In this study, 55% of genomes with *bla*_CTX-M-15_ were ST131; for the other ESC-R genes, the most common ST (either ST10, ST117 or ST131) represented only 9–17% of genomes. Second, the diversity of plasmid profiles, representing unique combinations of replicons, was similar for four out of the five main ESC-R genes. In this case, the exception was *bla*_CMY-2_, with a Simpson’s diversity index of 145.7 compared to the 72.9–81.9 observed with the other ESC-R genes. However, when the diversity of plasmid replicons in genomes carrying *bla*_CMY-2_ was assessed in individual compartments (Supplementary Fig. S14), the diversities within each compartment appeared to be similar to those for other ESC-R genes. The reason for this apparent contradiction is that the Simpson’s diversity index evaluates the number and abundance of unique combinations of plasmid replicons; it does not assess whether or not those plasmid replicons are the same. Here, each compartment had approximately the same diversity of plasmid replicon profiles with the *bla*_CMY-2_ gene, but these profiles were different between compartments, explaining the higher diversity when all compartments were considered together, as in Table 1. Given the difficulty in reconstructing full plasmid sequences with short-read genome data and, therefore, definitively linking ESC-R genes with plasmid types, we repeated this analysis on a subset of 313 genomes containing typeable plasmids, where the plasmid contig contained both the ESC-R gene and a replicon sequence. Similar to the main ESC-R gene dataset of 1343 genomes, this approach identified *bla*_CTX-M-15_ with the lowest ST diversity and highest ratio of plasmid replicon to ST diversity. In contrast to the main ESC-R gene dataset where *bla*_CMY-2_ genomes had the highest plasmid replicon diversity, in the typeable plasmid subset *bla*_CTX-M-15_ and *bla*_SHV-12_ had the highest plasmid replicon diversity. This discrepancy is likely because of the differential recovery of typeable plasmids; due to the multiple ISs in IncF plasmids, IncF plasmids represented a lower proportion of plasmids in the typeable plasmid subset (26.8%) than in the main ESC-R gene dataset (36.7%) (Supplementary Table S13). As IncF plasmids are highly diverse^60^, we used the replicon sequence typing (RST) scheme for IncF plasmids^61^ in these analyses to subdivide them beyond IncF. Therefore, the lower proportion of IncF plasmids in the typeable plasmid subset would have an even greater effect on plasmid replicon diversity compared to the main ESC-R gene dataset. Conversely, IncI1 plasmids were overrepresented in the typeable plasmid subset (62.9%) vs the main ESC-R gene dataset (15.3%). Furthermore, as specific ESC-R genes have associations with specific plasmid Inc types, such as *bla*_CTX-M-15_ with IncF (Supplementary Table S8), the typeable plasmid subset is not representative of the main ESC-R gene dataset.

The comparative use of ST and plasmid replicon diversities for different ESC-R genes can shed light into their evolutionary history and epidemiology. Using *bla*_CMY-2_ from the main ESC-R gene dataset as an example, the ST diversity was much higher in genomes derived from French humans, where this ESC-R emerged more recently than in Canada and, therefore, may not have yet undergone the same process of purifying selection. The ratios of ST to plasmid diversity for each ESC-R gene also identified differences in how these genes spread. A higher ratio could be due to two factors: (1) the ST diversity is relatively low, as was the case for *bla*_CTX-M-15_ in both the main ESC-R gene dataset and typeable plasmid subset, reflecting clonal expansion of lineages with *bla*_CTX-M-15_ carried on either plasmids or the chromosome as the predominant mechanism of spread, or (2) the plasmid replicon diversity is relatively high, as was the case for *bla*_CMY-2_ in the main ESC-R gene dataset, suggesting the gene is highly mobile between plasmids. While acknowledging the typeable plasmid subset is not representative of the main ESC-R gene dataset, examining those plasmid contigs with both ESC-R genes and plasmid replicons provides further insight into the mechanisms of ESC-R spread. This revealed dominant plasmid types associated with *bla*_CTX-M-1_ and *bla*_CTX-M-14_—IncI1 plasmid sequence type (pST) 3 plasmids with *bla*_CTX-M-1_ and IncF (subtype F2:A-:B-) plasmids with *bla*_CTX-M-14_—suggesting the spread of these genes is mainly due to dominant plasmids across different genetic backgrounds. The higher ST:plasmid diversity ratio of *bla*_SHV-12_ in the typeable plasmid subset is a result of relatively low ST and high plasmid diversity, which suggest that both ST clonal expansion (here, mainly ST117) and plasmids contribute to its spread. In the 26 genomes with *bla*_SHV-12_ typeable plasmids, ST117 comprised 30.8% of the STs harbouring *bla*_SHV-12_ IncF plasmids, while other STs carried IncF and IncI1 plasmids. When comparing the plasmid subtypes between *E. coli* and non-*E. coli*, we found three common plasmid subtypes (IncN pST1 *bla*_CTX-M-1_, IncI1 pST12 *bla*_CMY-2_ and IncI1 pST26 *bla*_SHV-12_), which suggest that some plasmid subtypes are crossing species borders and mobilising the ESC-R genes between bacterial species (Supplementary Table S14). This result supports previous observations where plasmid replicons^62^ and ESC-R genes were detected in diverse Enterobacterales species^14,18^.

It is important to consider the limitations of these data when interpreting the results. The bacterial collections from which isolates were selected for sequencing were generated for a variety of purposes, including surveillance and research projects, and as such utilised different inclusion criteria and methods as outlined in the Supplementary Material. The human isolates from France and Canada and the animal isolates from Germany and France were from cases of clinical disease, whereas animal isolates from Canada were from clinical cases and asymptomatic carriage. To reduce potential bias, isolates collected over the same time period were randomly selected from the different collections proportionately by year and from a diverse assortment of host species. Importantly, the identification of ESC-R was based on phenotypic testing, thus allowing comparison of the identity and diversity of ESC-R genes between compartments and was thus the focus of this study. To ensure the most robust inference from this dataset, we also restricted statistical comparisons to those ecological compartments with good representation, such as farm animals and humans from Canada and France when analysing the temporal trends in ESC-R genes. For the same reason, in the diversity analyses we took multiple random subsets of ecological compartments with larger sample sizes to comparable sizes of ecological compartments with smaller sample sizes. One striking result was the comparatively high diversity of ESC-R genes in genomes derived from French humans; these genomes were collected over the entire study period and from various parts of the country. Although the sampling strategy cannot be ruled out as a causative factor in the results, the underlying causes for this greater diversity of ESC-R in *E. coli* from humans in France warrants further investigation. Through the plasmidome analysis, we showed that the included 79 non-typeable plasmids were closely related to the typeable plasmids, demonstrating their likely Inc type but also the limitations of assembly with short-read data. Thus, large scale long-read sequence data are necessary to identify reliably the location of ESC-R genes to the chromosome or to specific plasmids, as we demonstrated with the 20 genomes for which we used long-read sequence data to validate the ESC-R gene location. With such data, a greater resolution of the plasmidome network and transmission can be obtained.

The differing trends and types of ESC-R in the countries examined could be due in part to different amounts of antimicrobial use (AMU). In Europe, the use of antimicrobials agents as growth promoters in animals has been stopped since 2006, and overall sales of veterinary antimicrobial agents have decreased, including in France^63^, Germany^64^ and the UK^64^. In comparison, Canada stopped the use of antimicrobials for growth promotion in 2016 and had higher sales for use in animals, with the predominant antimicrobial agents sold being tetracyclines, beta-lactams, macrolides and diaminopyrimidines-sulfonamides^65^. This contrast is perhaps reflected in the results of this study identifying an increase in resistance to sulfonamides and tetracyclines over the study period in chicken isolates from Canada. A recent study on AMU and AMR in broiler chickens in Canada during 2013-2019 showed that resistance to some antimicrobial classes decreased due to an overall reduction in AMU, but other antimicrobial classes, such as aminoglycosides, increased; this could be associated with the increased use of aminoglycosides in water to treat disease^66^. There can be differences in AMU between humans and animals by antimicrobial class, and between different species of animals^63,67^, which may also influence the prevalence of resistance to specific antimicrobials. However, there are limited data available on antimicrobial consumption and there are other factors, such as genetic linkage, which make understanding the causal relationships of use and resistance complex. Combining the high-resolution data conferred by WGS to understand genetic linkage of AMR genes along with greater available of AMU data will facilitate greater understanding of the relative contributions of different drivers of AMR.

In conclusion, this study confirmed the changes over time of the relative importance of ESBL and AmpC genes causing resistance to ESC drugs in Europe and North America. There was variation in the major ESC-R gene frequency between compartments (country + source), and in the main mechanism, clonality and/or plasmids, responsible for the dissemination of these genes. In addition, the co-occurrence of ESC-R genes with other AMR determinants was higher in some compartments in different countries. The changing patterns of antimicrobial use may be associated with the changing trends of resistance, but more data are required; given the demonstrated differences between compartments, the collection/addition of AMU data to WGS in future studies will enable greater understanding of this link. Utilising WGS to obtain high-resolution data not only to identify the type and diversity of ESC-R genes present, but also linking this information to bacterial host and plasmid diversity, offers deeper insight into the epidemiology of resistance to these critical drugs. Further in-depth study of these plasmids and the role of other mobile genetic elements, such as transposons, in the transmission and global epidemiology of ESC-R is required, and will be facilitated by the increasing feasibility of large scale long-read genome sequencing to generate fully contiguated genome sequences. In line with the conclusions of other studies^26^, these results highlight the need for long-term internationally harmonised monitoring in order to understand the dynamics and relative importance of different sources to the burden of AMR over time.

## Methods

### Bacterial isolates

All ESC-R isolates included in this study were selected independent of the genetic mechanism causing the resistance. The majority (84%) of isolates in this study were selected to include representatives from different ecological compartments (human, animal, food/meat) of ESC-R *E. coli* isolated from 2008 to 2016. These isolates were randomly selected from available culture collections across three countries: France, Germany and Canada. The remaining 16% of isolates were selected to provide context, namely: (1) ESC-R *E. coli* isolates obtained prior to 2008 or after 2016 (4%); (2) ESC-R isolates of bacterial species other than *E. coli* from 2008 to 2016 (5.6%); and (3) ESC-susceptible *E. coli* (6.5%). With this strategy, 1524 isolates were selected: 718 isolates from Canada, 607 from France and 199 from Germany (Table S1). Bacterial collection and isolation methods for each subset are described in the Supplemental methods, section 1.

In addition, genomes from previous studies were included. This included 158 from Germany^22^ and 248 from the UK^32,33^; these genomes were selected following a similar approach of sample selection as described above, where all genomes available between 2008 and 2016 were included and a small number of genomes collected prior to 2008 and/or genomes of ESC-susceptible isolates were selected.

### Whole genome data, MLST and typing of *fimH* allele and phylogroups

Paired-end short-read whole genome sequence data were generated on the Illumina platform for the 1524 isolates selected above, and fastq data available from the previous studies for the German (study accession PRJEB23663)^22^ and UK genomes (study accessions PRJEB4681, PRJEB8774 and PRJEB8776)^32,33^ were downloaded from the European Nucleotide Archive (http://www.ebi.ac.uk/ena). The methods for DNA extraction and WGS are detailed for each subset in the Supplemental method, section 1. Raw reads were processed with Trimmomatic v0.33^68^ to remove adapters and poor quality bases. The trimmed reads were used to assemble draft genomes using Spades v3.11.1^69^; summary statistics of the assemblies were obtained using Quast v4.5^70^, and assemblies with a high number of contigs (>900 contigs) were excluded. Genome quality was also assessed using CheckM v1.1.2^71^ where genomes with completeness below 95.0% and/or contamination in the assembly with more than 15.0% were excluded. Centrifuge 1.0.3-beta^72^ and Kleborate v2.0.4^73^ were used for the species assignment. In cases where species assignment differed between Centrifuge and Kleborate, FastANI v1.32^74^ was applied and compared with NCBI genome references. BWA v0.7.17^75^, Samtools v1.9^76^ and Bcftools v1.8^77^ were used to map reads against their corresponding assemblies for the largest four contigs to estimate sequencing mean chromosomal depth coverage.

The Achtman sequence typing scheme^78^ was applied using ARIBA v2.13.5^79^ to assign the multilocus sequence type (MLST) for each genome. The sequence type (ST) was allocated to clonal complexes (CC) using the *E. coli* database available from the Enterobase website^80^. The *fimH* alleles for *E. coli* were downloaded from the Center for Genomic Epidemiology (http://www.genomicepidemiology.org/) and used with ARIBA for typing the alleles. Phylotyping for *E. coli* was performed using the ClermonTyping v1.4.1^36^ tool.

### Assessment of potential inclusion of outbreak isolates

To determine whether or not highly similar outbreak isolates were included in the dataset, which could affect the proportion of specific ESC-R genes and temporal analysis, pairwise SNP distances were analysed within the core genome for each main ST: ST131, ST10 and ST117. To obtain the core genome, reads were mapped to the relevant reference genome using Snippy v4.3.6^81^. The reference genomes were: EC958 (NZ_HG941718.1; https://www.ncbi.nlm.nih.gov/nuccore/NZ_HG941718.1) for ST131, K-12 MG1655 (NC_000913.3; https://www.ncbi.nlm.nih.gov/nuccore/NC_000913.3) for ST10 and MDR_56 (NZ_CP019903.1; https://www.ncbi.nlm.nih.gov/nuccore/NZ_CP019903.1) for ST117. Pairwise SNP differences were obtained using snp-dists v0.7.0 (https://github.com/tseemann/snp-dists). High similarity between pairs of isolates was considered to be differences ≤10 SNPs.

### In silico identification of AMR determinants and plasmid replicons

ARIBA v2.13.5^79^ was used to identify acquired AMR genes and plasmid replicons with the ResFinder^82^ and PlasmidFinder^61^ databases, respectively. ESBL and AmpC genes were identified from these databases. Mutations in gyrase genes (*gyrA* and *gyrB*) and topoisomerase IV genes (*parC* and *parE*) associated with quinolone resistance were also identified using ARIBA v2.13.5^79^ with the wild-type sequences as the input databases. The frequency and distribution of resistance determinants to ESC and other antimicrobial classes were analysed by country, by source (i.e., host species) and by compartment, which is defined as the unique combination of country and source. A similar approach was used for plasmid replicons and mutations in topoisomerase genes. The numbers of acquired AMR genes and plasmid replicons for each sample were compiled and compared by analysing the distribution of their frequencies among groups (country + ESC-R genes) for each source.

### Prediction of ESC-R gene location to plasmid or chromosome contig in de novo assembly

The prediction of contigs as plasmid or chromosomal origin was first achieved using MOB-suite v2.0^34^ with the mob_recon option with default setting. In addition, RFPlasmid^35^ was also used; a cut-off of ≥0.6 was used to identify the plasmid contigs. Resfinder^82^ and Plasmidfinder^61^ in Abricate v0.9.8 (https://github.com/tseemann/abricate) were used to identify ESC-R genes and plasmid replicon sequences in contigs. The plasmid subtyping was done using Blast+ v2.9.0^83^ and the plasmid MLST (pMLST) scheme (https://bitbucket.org/genomicepidemiology/pmlst_db/). Where the prediction of chromosome vs plasmid origin differed between MOB-suite and RFPlasmid, a subset of five genomes for the four most common ESC-R genes, for a total of 20 isolates, was randomly selected for long-read sequencing (see Supplemental Method, section 2, for further information on long-read data), to confirm the localization of the ESC-R gene either to a plasmid or the chromosome. The plasmidome analysis using PANINI^84^ was performed in the set of 313 typeable plasmids plus a subset of 79 non-typeable plasmids (identified with MOB-suite) carrying ESC-R genes recovered from short-read data (See Supplemental Method, Section 3).

### Diversity analysis of ST, ESC-R, AMR and plasmid replicon profiles

The diversities of STs, ESC-R genes, AMR profiles and plasmid replicon profiles were estimated as previously described for *Salmonella* Typhimurium^85^ using the vegan v2.5-6^86^ R package. Four diversity measures were calculated, covering a range of weightings of richness and abundance: SR (species richness), SE (Shannon entropy), SD (Simpson’s diversity) and reciprocal BP (Berger-Parker)^85,87^. The ESC-R profile was defined as the combination of unique ESC-R genes within a single genome. The AMR profile comprised the presence of all AMR determinants, including ESC-R genes and mutations in the topoisomerase genes. The diversity analysis was performed by compartment (country + source). As sample size varied, compartments with larger sample sizes were randomly subsampled to the size of the smaller compartment. The subsampling consisted of 10,000 permutations without replacement and the mean with 95% confidence intervals of diversity indices were calculated. The calculated values for SE, SD and BP were converted into the effective number of profiles prior to plotting the results^85^.

In addition, Simpson’s diversity (SD) for ST and plasmid replicon profiles were further investigated in two datasets: (1) in the main ESC-R gene dataset of 1343 *E. coli* genomes with the five main ESC-R genes, where we investigated diversity (i) within genomes carrying each major ESC-R genes, and (ii) between main compartments within each major ESC-R gene; (2) in the subset of 313 typeable ESC-R plasmids, where SD assessment of plasmid replicon profiles and their respective bacterial ST across ESC-R genes were performed; in this analysis, contigs predicted to be plasmids (identified through both MOB-suite and RFPlasmid) that were carrying both a main ESC-R gene and a replicon sequence were used. The diversity estimates were computed through the sample-size-based rarefaction and extrapolation method implemented in the iNEXT v2.0.20^88^ R^89^ package.

### Phylogenetic analysis

The draft assemblies were annotated using Prokka v1.13^90^ and the pangenome was analysed with Roary v3.12.0^91^; core genes were identified with 90% sequence similarity and presence in 95% of isolates. SNPs were extracted from the core gene alignment using snp-sites v2.5.1^92^ and used to construct a maximum likelihood tree using the HKY and discrete GAMMA nucleotide model including constant sites information in IQ-tree v1.6.11^93^. A phylogenetic tree was constructed for the entire *E. coli* (*n* = 1839) genome collection; due to the large sample size, the ultrafast bootstrap approximation^94^ -B 1000 was used. Separate phylogenies were also constructed for genomes containing each main ESC-R gene (*bla*_CTX-M-1_, *bla*_CTX-M-15_, *bla*_CMY-2_, *bla*_CTX-M-14_ and *bla*_SHV-12_) using subsets of the same core gene alignment. For the separate phylogenies, the standard nonparametric bootstrap^95^ -b 100 was applied. Metadata were plotted against the trees using the ggtree v2.0.2^96^ R^89^ package. The pangenome network analysis was also performed using PANINI^84^; details are provided in the Supplemental Method, Section 4.

### Statistical analysis

Fisher’s Exact Test from the rstatix’s v0.5.0^97^ R^89^ package was used to compare the proportions of the major ESC-R genes between host species (human vs animal) and continents (Europe vs North America), and similarly, between main compartments within each major ESC-R gene and for overall AMR class. A Bonferroni correction was applied to account for multiple hypothesis testing.

Kruskal-Wallis tests, also available in rstatix’s v0.5.0^97^, were used to compare the numbers of acquired AMR genes between groups constituted by country and ESC-R gene; groups with similar (statistically not significant) distributions were combined. The medians of the number of acquired AMR genes between the groups were compared through Mann–Whitney *U* tests.

To test for temporal trends in antimicrobial resistance, a logistic regression was applied, where the binary outcome variable was the presence or absence of a specific AMR determinant and the predictor variable was the year of isolation. To note, this analysis did not include a number of genomes from humans in France and Canada: 20 genomes that had both AmpC and ESBL genes and four AmpC-carrying genomes, as they were additional samples that were not originally selected with the same study selection strategy. Logistic regression analysis was done individually by compartment (country + source) for each ESC-R gene type (all AmpC genes, including *bla*_CMY_ variants and *bla*_DHA-1_; and all ESBL genes, including variants of *bla*_CTX-M_, *bla*_TEM_, *bla*_SHV_ and *bla*_VEB-1_), main ESC-R genes and other antimicrobial classes (aminoglycosides, sulfonamides, tetracyclines, other beta-lactams [non-ESBL and non-AmpC genes], diaminopyrimidines, macrolides, phenicols and quinolones/fluoroquinolones due to mutations in *gyrA*, *parC* and *parE*). The glm(y ~ x, family = “binomial”) function available in R^89^ was used to obtain the estimates, p-values based on z-scores (Wald statistic), odds ratio (OR) along with the 95% confidence intervals. A Bonferroni correction was applied to account for multiple hypothesis testing, as described above.

### Reporting summary

Further information on research design is available in the Nature Portfolio Reporting Summary linked to this article.

## Supplementary information

Supplementary Information

Reporting Summary

## Data Availability

Illumina sequence read data generated in this study have been deposited in the ENA under BioProject PRJEB38235, PRJEB42322, PRJNA523640, PRJNA556083, PRJNA740259 and PRJEB50837 and individual accession numbers are available in Source Data file. Long-read data for the subset of 20 genomes are deposited in the ENA under BioProject PRJEB54884; the accession numbers for each genome are placed in the Source Data file. Thus, the source data (Source Data file) provided with this paper lists accession numbers for each genome analysed in this study, alongside with the isolate collection metadata generated in this study. Source data are provided with this paper.

## Source data

Source Data
