# Supplementary material for: Dynamics of extended-spectrum cephalosporin resistance genes in Escherichia coli from Europe and North America
Source: Nat Commun. 2022 Dec 12;13:7490. doi: 10.1038/s41467-022-34970-7 (PMC9744880; doi:10.1038/s41467-022-34970-7)
Supplement: Supplementary file 1 — Supplementary Information [file 41467_2022_34970_MOESM1_ESM.pdf]

Supplementary Material

Dynamics of extended-spectrum cephalosporin resistance genes in *Escherichia coli* from Europe and North America

Roxana Zamudio[1], Patrick Boerlin[2], Racha Beyrouthy[3,4], Jean-Yves Madec[5], Stefan Schwarz[6,7], Michael R. Mulvey[8], George G. Zhanel[9], Ashley Cormier[2], Gabhan Chalmers[2], Richard Bonnet[3,4], Marisa Haenni[5], Inga Eichhorn[6,7], Heike Kaspar[10], Raquel Garcia-Fierro[5], James L. N. Wood[11], Alison E. Mather[1,12*]

[1]Quadram Institute Bioscience, Norwich Research Park, Norwich, NR4 7UQ, United Kingdom
[2]Department of Pathobiology, University of Guelph, Guelph, N1G 2W1, Canada
[3]Microbes Intestin Inflammation et Susceptibilité de l'Hôte (M2ISH), Faculté de Médecine, Université Clermont Auvergne, Clermont-Ferrand, 63001, France
[4]Centre National de Référence de la résistance aux antibiotiques, Centre Hospitalier Universitaire de Clermont-Ferrand, Clermont-Ferrand, 63000, France
[5]Unité Antibiorésistance et Virulence Bactériennes, Anses Laboratoire de Lyon, Université de Lyon, Lyon, France
[6]Institute of Microbiology and Epizootics, Department of Veterinary Medicine, Freie Universität Berlin, Berlin, 14163, Germany
[7]Veterinary Centre for Resistance Research (TZR), Department of Veterinary Medicine, Freie Universität Berlin, Berlin, 14163, Germany
[8]National Microbiology Laboratory, Public Health Agency of Canada, Winnipeg, Manitoba, R3E 3R2, Canada
[9]Department of Medical Microbiology and Infectious Diseases, Max Rady College of Medicine, Rady Faculty of Health Sciences, University of Manitoba, Winnipeg, Manitoba, R3E 0J9, Canada
[10]Department Method Standardisation, Resistance to Antibiotics Unit Monitoring of Resistance to Antibiotics, Federal Office of Consumer Protection and Food Safety, Berlin, 12277, Germany
[11]Department of Veterinary Medicine, University of Cambridge, Cambridge, CB3 0ES, United Kingdom
[12]University of East Anglia, Norwich, NR4 7TJ, United Kingdom

*Corresponding author: alison.mather@quadram.ac.uk

TABLE OF CONTENTS

SUPPLEMENTAL METHODS 2

Section 1: Bacterial isolates and whole genome sequencing 2

1.1. Human isolates from Canada 2

1.2. Animal, food (meat) and wastewater samples from Canada 3

1.3. Human isolates from France 3

1.4. Animal and food isolates from France 4

1.5. Animal isolates from Germany 4

Section 2: Long-read data and hybrid assembly 4

Section 3: Plasmidome analysis of plasmids linked with ESC-R genes from short-read data........ 5

Section 4: Pangenome analysis of 1,818 *E. coli* genomes 5

SUPPLEMENTAL FIGURES 6

SUPPLEMENTAL TABLES 28

SUPPLEMENTAL REFERENCES 45

SUPPLEMENTAL METHODS

Section 1: Bacterial isolates and whole genome sequencing

1.1. Human isolates from Canada

Canadian bacterial isolates from human sources were obtained as part of the Canadian Ward (CANWARD) and CAN-ICU Surveillance Studies coordinated by the Canadian Antimicrobial Resistance Alliance (CARA) based out of the Health Science Centre in Winnipeg, Manitoba.
In brief, Canadian hospital laboratories were asked to submit clinically significant isolates (consecutive, one per patient, per infection site) from patients with respiratory, urine, wound and bloodstream infections. The number of tertiary care centres participating in the CANWARD surveillance study by year included 12 centres in 2007, 10 in 2008, 15 in 2009, 14 in 2010, 15 in 2011, 12 in 2012, 15 in 2013, 13 in 2014, 13 in 2015, 13 in 2016, 14 in 2017, and 12 in 2018, representing eight of the 10 Canadian provinces.

The CAN-ICU Study was conducted from September 2005 to June 2006 at 19 medical centres with active ICUs from across Canada. Centres submitted clinically relevant isolates (one pathogen per cultured site per patient) from patients in medical, surgical, and pediatric ICUs. Participating centres were asked to submit 300 consecutive clinically significant bacterial pathogens isolated from blood, urine, wound, and respiratory tract specimens. Isolates were deemed clinically significant according to each submitting laboratory's own standard protocol.

Genomic DNA for whole genome sequencing (WGS) was extracted from cultures grown overnight at 37°C in Luria Broth, using the Epicentre MasterPure™ Complete kits (Mandel Scientific, Guelph, ON, Canada). Isolates were then submitted to the Genomics Core Facility at the Public Health Agency's National Microbiology Laboratory for library preparation and sequencing. Paired-end libraries were constructed using the Nextera XT Library Prep Kit (Illumina, San Diego, CA) and 150-bp paired-end indexed reads were generated on the Illumina MiSeq platform. Raw sequences were provided by the Genomics Core Facility for further analysis.

An additional ten isolates of human origin (nine *E. coli*, one *K. pneumoniae*) from Canada were included, deriving from different patients in a hospital from Ontario. These isolates were cultured, DNA extracted and whole genome sequenced following the same methods described in the second paragraph in section 1.2.

1.2. Animal, food (meat) and wastewater samples from Canada

The Canadian Integrated Program for Antimicrobial Resistance Surveillance (CIPARS) monitors antimicrobial resistance in enteric bacteria isolated from human and agrifood sources across Canada. CIPARS conducts antimicrobial susceptibility testing (AST) on all *Escherichia coli* and *Salmonella* serovars collected from surveillance of chicken, turkey, pig, and cattle sources (clinical, manure, caecal) and retail meat products. Further information on the sampling and testing methods used by CIPARS can be found in the annual reports.[1] This study used a convenience sample of cefoxitin resistant *E. coli* (n=177) and *Salmonella* (n=41) isolates collected from 2012 to 2017 from agrifood that were resistant to cefoxitin as determined by broth microdilution with the Sensititre™ Complete Automated AST System (CMV4AGNF plate). These isolates were cultured, DNA extracted and WGS following the same methods described for the Canadian human isolates from the CANWARD and CARA surveillance studies (section 1.1 described above).

Other sets of *E. coli* and non-*E. coli* isolates (n=130 and n=10 isolates, respectively) from Canada were from animal clinical cases and asymptomatic carriage, food and environment (wastewater) from different bacterial collections across the country. Detailed information of each collection was previously reported[2–4]. Samples were enriched in EC broth containing 2 mg/L of cefotaxime overnight at 37°C. A 10 uL loopful was then streaked onto MacConkey agar plates containing 1 mg/L ceftriaxone, and non-lactose fermenting colonies were purified and identified to the species level using MALDI-TOF MS (Bruker Daltonik GmbH, Bremen, Germany) before further characterisation. Genomic DNA extractions were performed using the Epicentre MasterPure DNA Purification kit for cell samples, followed by the complete removal of RNA (Epicentre, Madison, WI). Short read sequences were obtained using MiSeq and HiSeq technology (Illumina, San Diego, CA) performed at the Advanced Analysis Centre, University of Guelph, ON, and McGill University and Génome Québec Innovation Centre, McGill University, QC, following library preparation using Nextera XT kits (Illumina).

1.3. Human isolates from France

Human isolates came from the French national reference center for antibiotic resistance (78.5%), and from specific surveillance studies of third-generation resistance *Escherichia coli* (21.5%) collected in France. The third-generation cephalosporins (C3G) resistant producing *Enterobacteriaceae* included in this study were collected from the majority of regions in France (n=11/13) and the majority of isolates were collected in the three most densely populated region: Auvergne Rhone Alpes (45%), Ile de France (27%) and Provence-Alpes-Côte d'Azur (10%). Bacteria were isolated on non-selective media. WGS was performed using the next-generation sequencing platform of the teaching hospital of Clermont-Ferrand, France. DNA was extracted with a DNeasy UltraClean Microbial kit (Qiagen). The libraries were prepared with a Nextera XT Kit (Illumina, San Diego, CA, USA), and sequenced by the Illumina MiSeq system generating 2 × 301-base pair (bp) paired-end reads.

1.4. Animal and food isolates from France

Non-human isolates came either from the Resapath network that collects bacteria from sick animals (all included isolates from cats, dogs, horses and the majority of isolates from cattle), from specific surveillance studies at the slaughterhouse (n=32 *E. coli* from cattle) and from one specific study on chicken meat. Bacteria from the Resapath were isolated in peripheral veterinary laboratories on non-selective media. Bacteria from the two specific studies were isolates on selective ChromID ESBL medium (Biomérieux, Marcy l'Etoile, France). DNA from all isolates was extracted using the NucleoSpin Microbial DNA mini kit (Macherey-Nagel, Hoerdt, France). WGS was outsourced to the University of Clermont-Ferrand and DNA were processed as described above for French human isolates.

1.5. Animal isolates from Germany

The 199 isolates from Germany were obtained from the strain collection of the national antimicrobial resistance monitoring program of animal pathogenic bacteria in Germany, GERM-Vet, conducted by the Federal Office of Consumer Protection and Food Safety (BVL), Berlin, Germany. Veterinary diagnostic laboratories from all over Germany provide pre-identified bacteria from acutely diseased animals, which have not been treated with antimicrobial agents in the six weeks prior to sampling, according to a defined sampling plan to this program. When the bacteria arrive at BVL, they are inoculated on non-selective media (i.e. sheep blood agar plates (Oxoid, Wesel, Germany)) for purity checks and are confirmed for their species assignment by MALDI-TOF mass spectrometry (Bruker, Bremen, Germany). Thereafter, the bacteria were subjected to antimicrobial susceptibility testing (AST) by broth microdilution according to CLSI standards using commercial microtiter plates (MSC diagnostics Swalmen, The Netherlands). The panel of antimicrobial agents tested included β-lactams (penicillin, ampicillin, amoxicillin-clavulanic acid, ceftiofur, cefquinome, cephalothin, cefotaxime, cefoperazone), macrolides (tilmicosin, tulathromycin), (fluoro)quinolones (nalidixic acid, ciprofloxacin, enrofloxacin, marbofloxacin), tetracyclines (tetracycline, doxycycline), aminoglycosides (gentamicin, neomycin, streptomycin), a phenicol (florfenicol), a pleuromutilin (tiamulin), a polypeptide (colistin) and the combination of folate pathway antagonists trimethoprim-sulfamethoxazole. *Escherichia coli* ATCC25922 served as a quality control strain in AST.

DNA of the German isolates was prepared using the MasterPure DNA Purification Kit (Epicentre, Madison, WI, USA) according to the manufacturer's recommendations. DNA libraries were prepared using the Nextera XT Library Preparation Kit (Illumina, San Diego, CA, USA) as recommended by the manufacturer. Sequencing was performed on the Illumina MiSeq (Illumina) platform using the Miseq Reagent Kit v3 and the Nextera XT index kit2 v2 with an approximately 100-fold coverage.

Section 2: Long-read data and hybrid assembly

Long-read sequencing of 20 isolates was performed using a MinION (Oxford Nanopore Technologies, Oxford, United Kingdom). Base-calling of the fast5 files and demultiplexing of the Nanopore reads were performed using Guppy Basecaller v4.5.4 and Guppy Barcoder v4.5.4 (Oxford Nanopore Technologies), respectively. Adapters from long reads were removed with Porechop v0.2.4[5]. Filtlong v0.2.0 (https://github.com/rrwick/Filtlong) was used to remove reads <2Kpb in length and low quality reads; read files with a very large size were reduced to a 500 Mbp subset. Hybrid assembly using short and long reads for each

isolate was obtained with Unicycler v0.4.8[6] using the "bold" mode option to get complete assembly. Hybrid assemblies were polished using the Illumina reads with Pilon v1.22[7] up to five or ten cycles of polishing. The mapping of the ESC-R genes to chromosome or plasmids was done using Abricate v0.9.8 (https://github.com/tseemann/abricate) and the Resfinder[8] database.

Section 3: Plasmidome analysis of plasmids linked with ESC-R genes from short-read data

The predicted plasmid contigs (recovered from short-read data) harboring the main ESC-R genes were annotated using Prokka v1.13[9]. With Roary v3.12.0[10], the core genes were identified with 90% sequence similarity and presence in 95% of isolates. The Roary matrix of gene presence absence was used to generate a gene content (plasmidome) network through the PANINI[11] web tool. The plasmidome network was annotated with the metadata (ESC-R gene, plasmid Inc. type, plasmid subtypes, country, source and year) and then visualized.

In this analysis, all 313 typeable ESC-R plasmid contigs were included (136 $bla_{\text{CTX-M-1}}$, 23 $bla_{\text{CTX-M-15}}$, 88 $bla_{\text{CMY-2}}$, 40 $bla_{\text{CTX-M-14}}$ and 26 $bla_{\text{SHV-12}}$) plus 79 non-typeable plasmids. The latter represents a subset of the 823 non-typeable plasmids identified, and were selected using the following criteria: they were predicted as plasmids by both MOB-suite (which reconstructs plasmids) and RFPlasmid, and so should be longer sequences and thus contain more information than those non-typeable plasmids predicted by RFPlasmid alone. The 79 non-typeable plasmids were added in the plasmidome analysis to investigate how they relate to the typeable plasmids.

Section 4: Pangenome analysis of 1,818 *E. coli* genomes

Similar to the analysis described in the above section, the gene presence absence matrix obtained from 1,818 *E. coli* genomes using Roary was used to generate the gene context network, which was annotated with the metadata such as phylogroup, main ST/CC, ESC-R, country, source and year.

SUPPLEMENTAL FIGURES

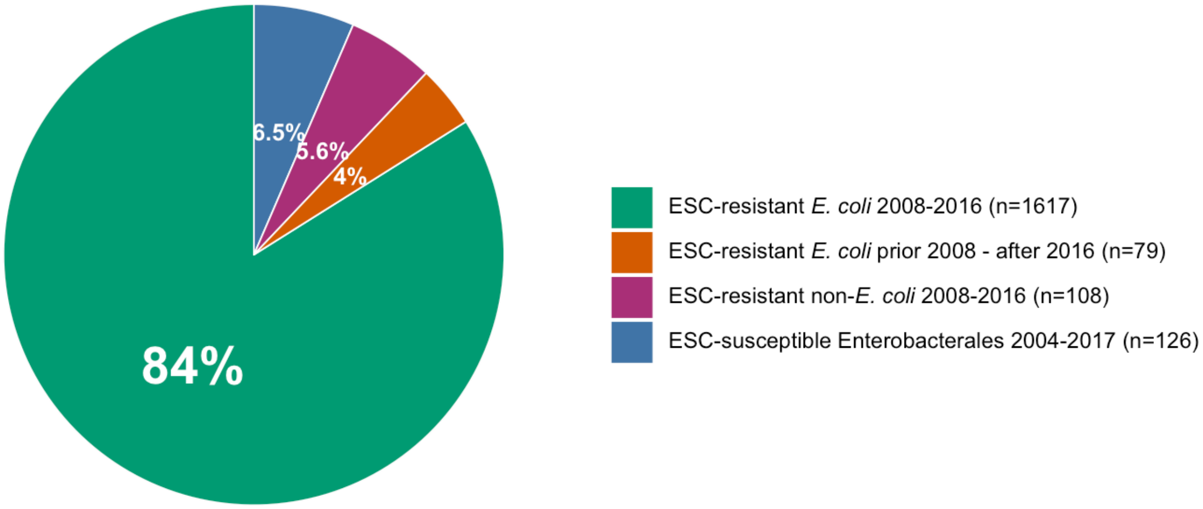

Figure S1. Selected bacterial samples according to study design. Proportion of included samples which were *E. coli* ESC resistant, non-*E. coli* ESC resistant, *Enterobacteriaceae* ESC susceptible (indicated by colour, as per inset legend). In the last group mainly are *E. coli* between 2008 to 2016 (n=100), and the remaining are *Klebsiella pneumoniae* (n=2), *Citrobacter freundii* (n=1) and *Raoultella ornithinolytica* (n=1), and *E. coli* from 2004 (n=1), 2006 (n=1) and 2017 (n=20). ESC: extended-spectrum cephalosporin.

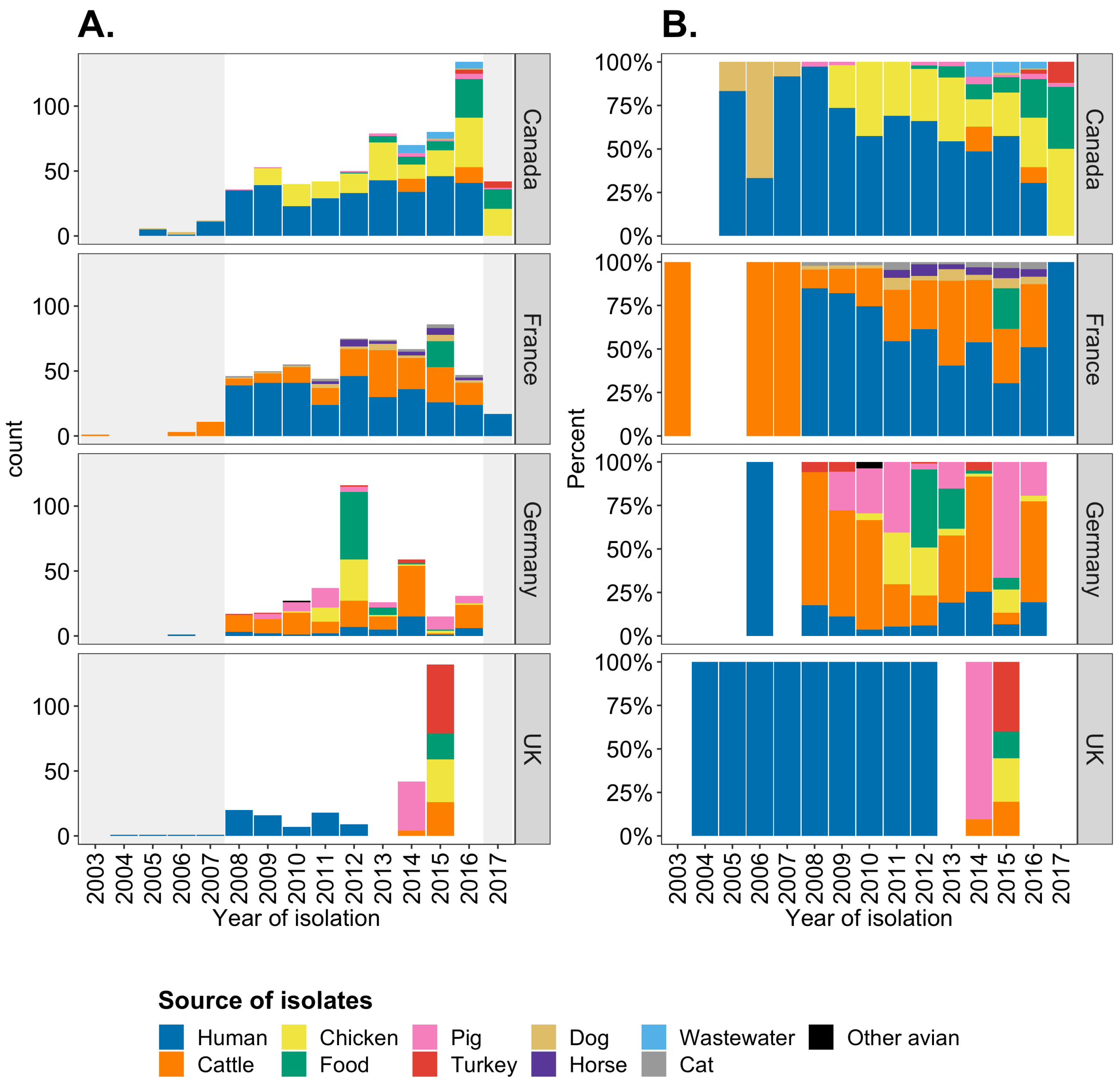

Figure S2. Distribution of the *E. coli* collection over time by country and source. (A) Number of isolates over time by country and source. **(B)** Percentage of isolates over time by country and source. The colour of the bar represents the source.

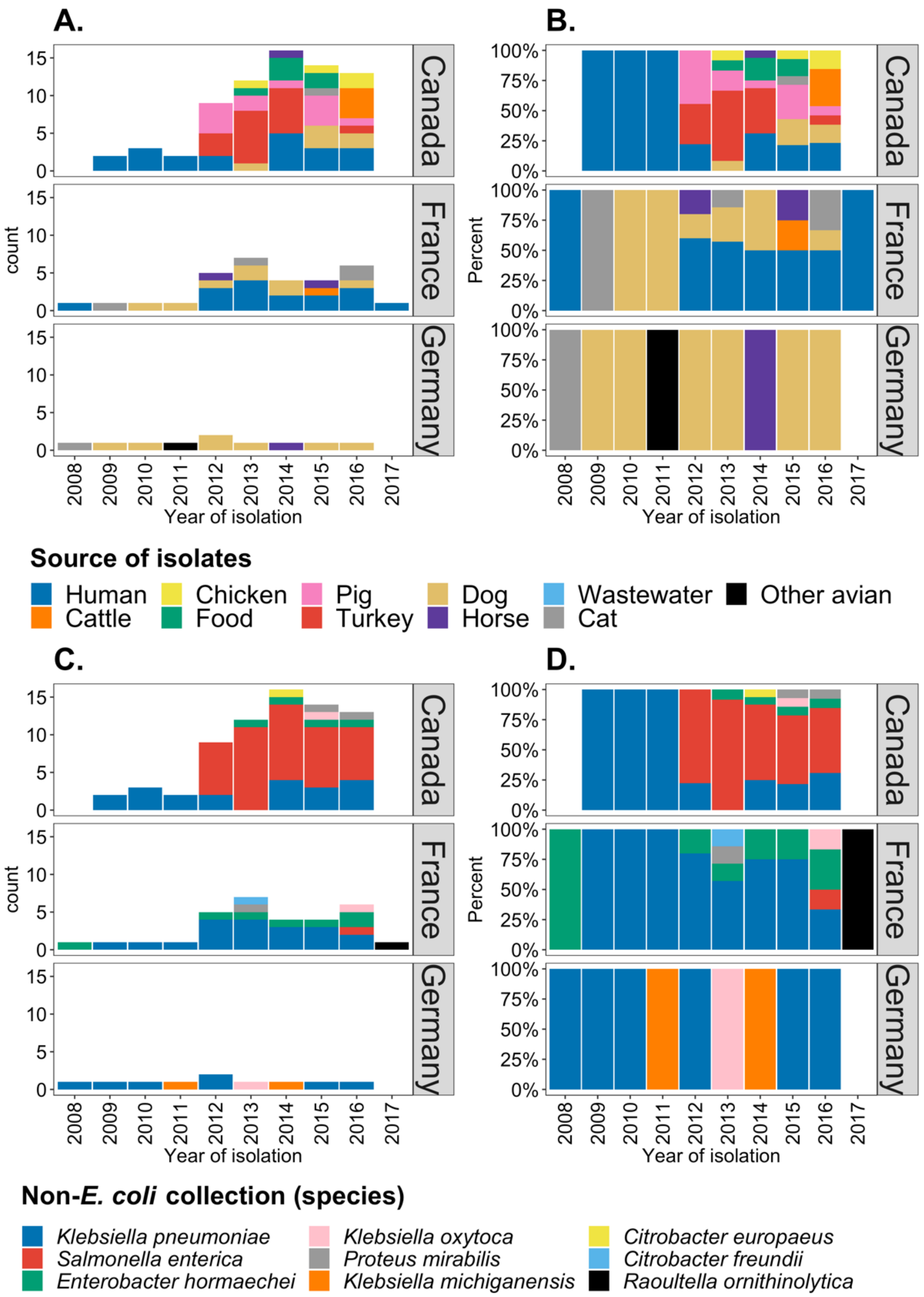

Figure S3. Distribution of the non-*E. coli* collection over time by country, source and bacterial species. **(A)** Number and **(B)** percentage of isolates over time by country and source. Bar colour represents source. **(C)** Number and **(D)** percentage of isolates over time by country and species. Bar colour represents bacterial species.

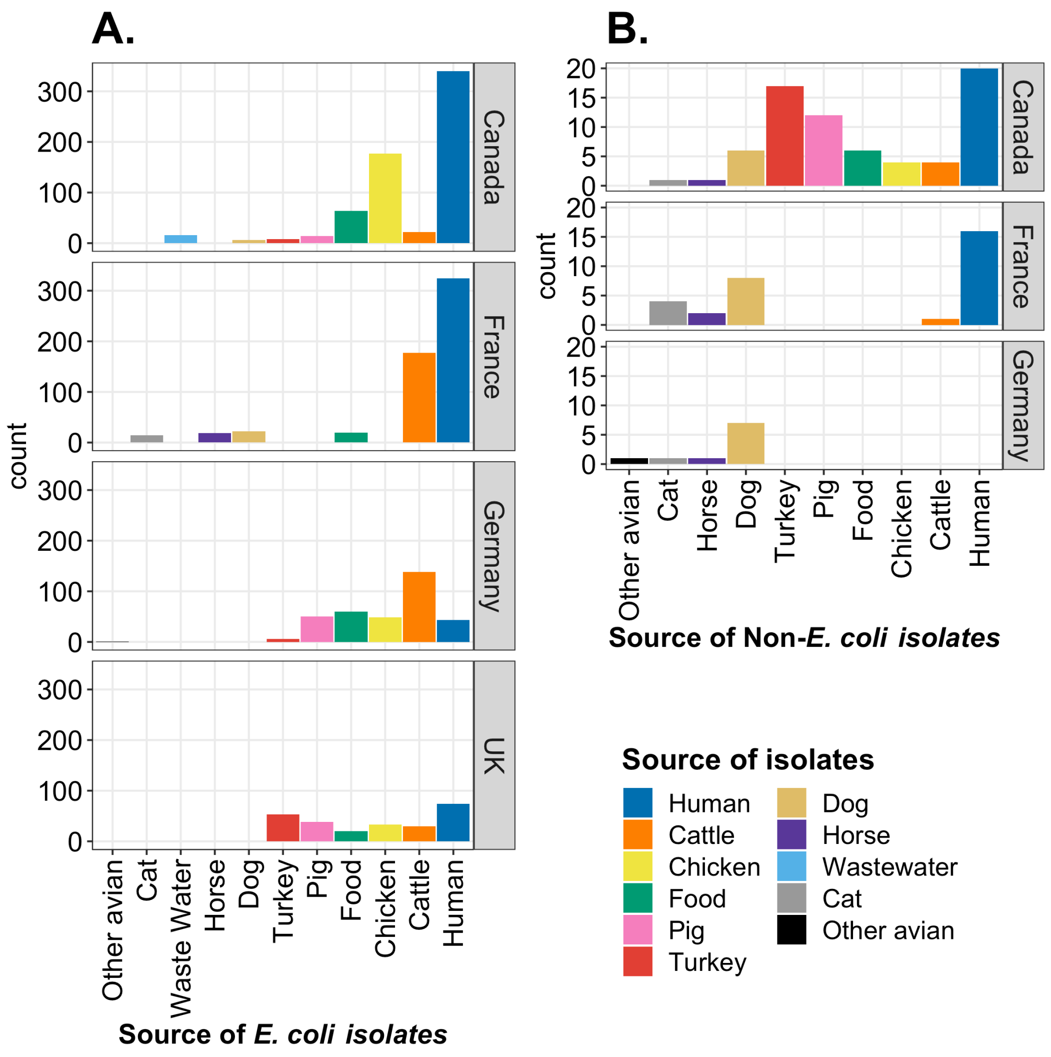

Figure S4. Distribution of the *E. coli* and non-*E. coli* isolates by compartment (country + source). (A) Sample distribution for *E. coli* collection, and **(B)** non-*E. coli* collection. Bar colour represents source.

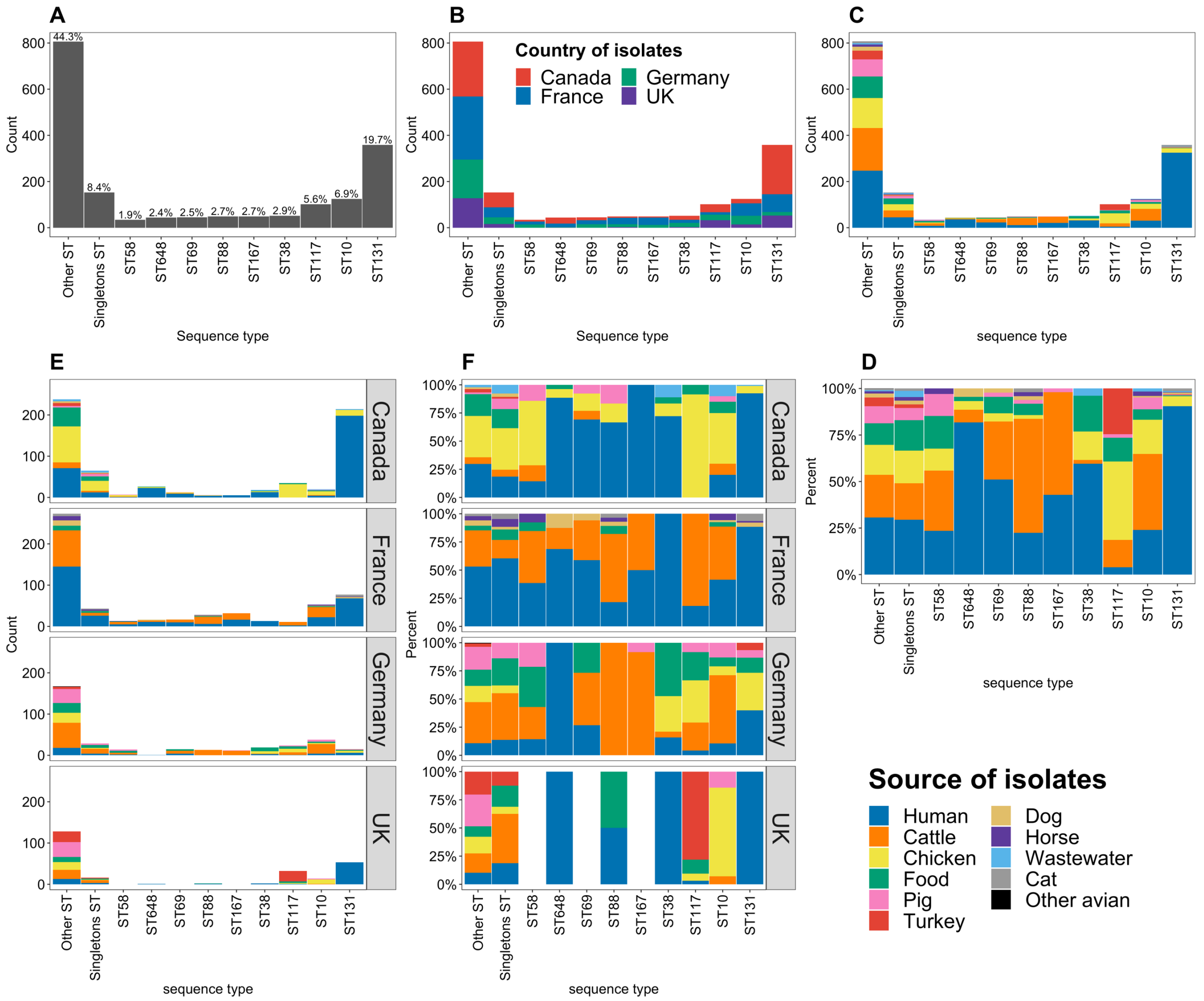

Figure S5. Distribution of sequence types (STs) in the *E. coli* collection. (A) Numbers of genomes by ST. **(B)** Number of isolates by country and **(C)** source within each ST; the colour of the barplot is linked to country and to source, respectively. **(D)** Percentage of sources within each ST. **E)** Count and **(F)** percentage of sources by country within each ST. Colour of the barplot represents source.

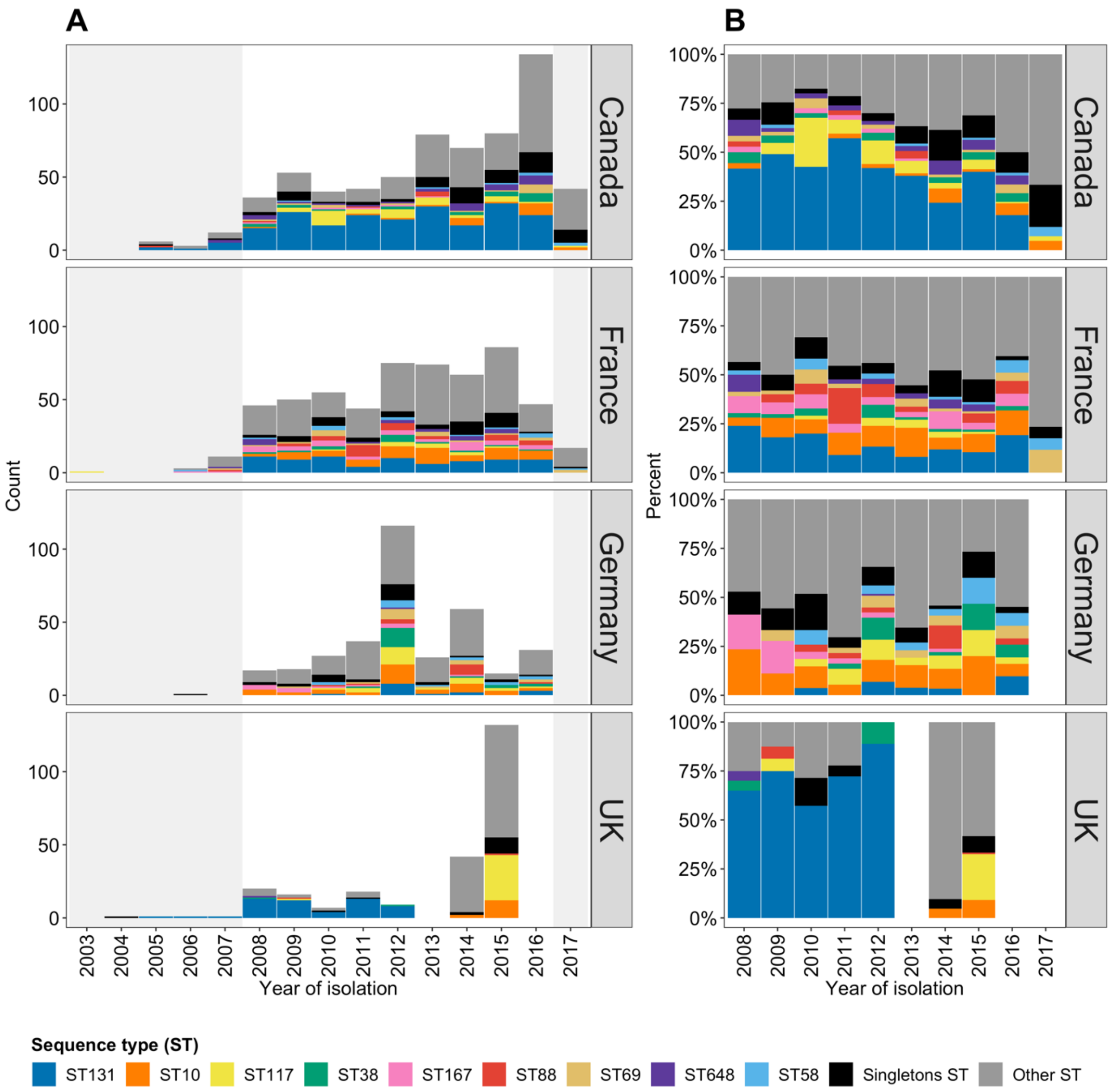

Figure S6. Distribution of STs over time by country in the *E. coli* collection. (A) number of isolates collected between 2003 and 2017. **(B)** Percentage of isolates between 2008 and 2017. Colour represents ST: "Singletons ST" represents STs observed only once; "Other ST" represents STs comprising less than 2% of total genomes.

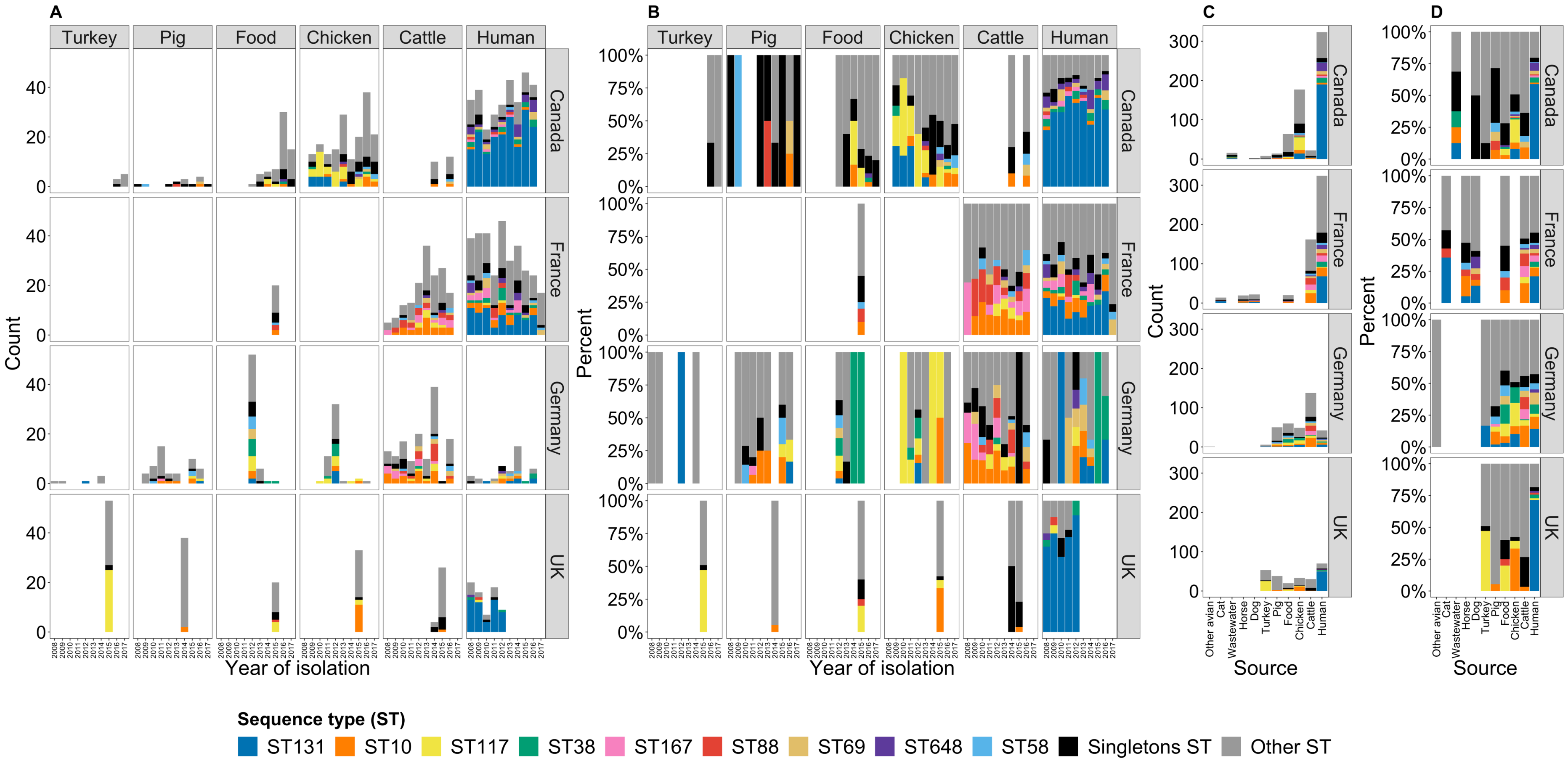

Figure S7. Distribution of STs over time by country and source in the *E. coli* collection. Distribution of STs for frequent sources: human, cattle, chicken, food, pig and turkey. **(A)** Number and **(B)** percentage of isolates by country and source between 2008 and 2017. **(C)** Number and **(D)** percentage of isolates by country and source. Colour represents ST: "Singletons ST" represents STs observed only once; "Other ST" represents STs comprising less than 2% of total genomes.

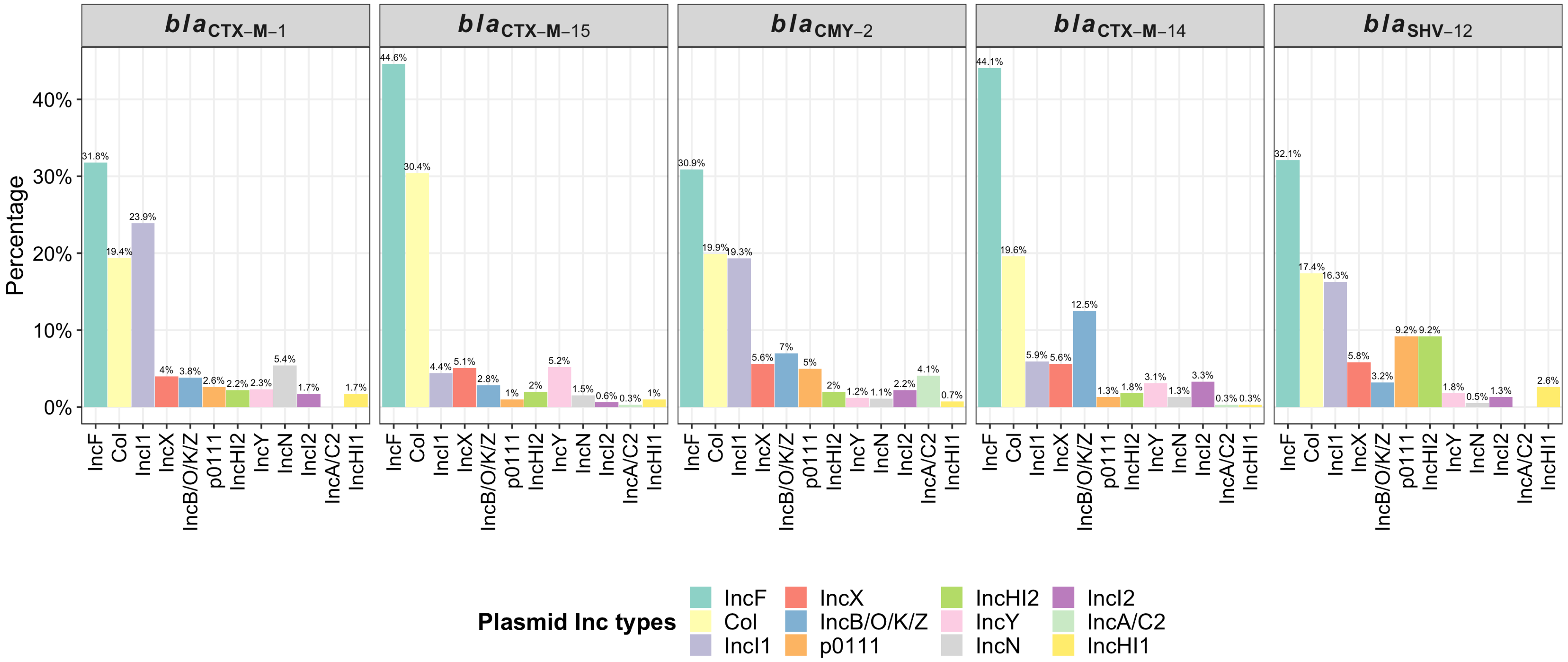

Figure S8. Percentage of main plasmid incompatibility (Inc) types based on replicons within genomes carrying each major ESC-R gene. Bars are coloured by plasmid Inc types (as per inset legend).

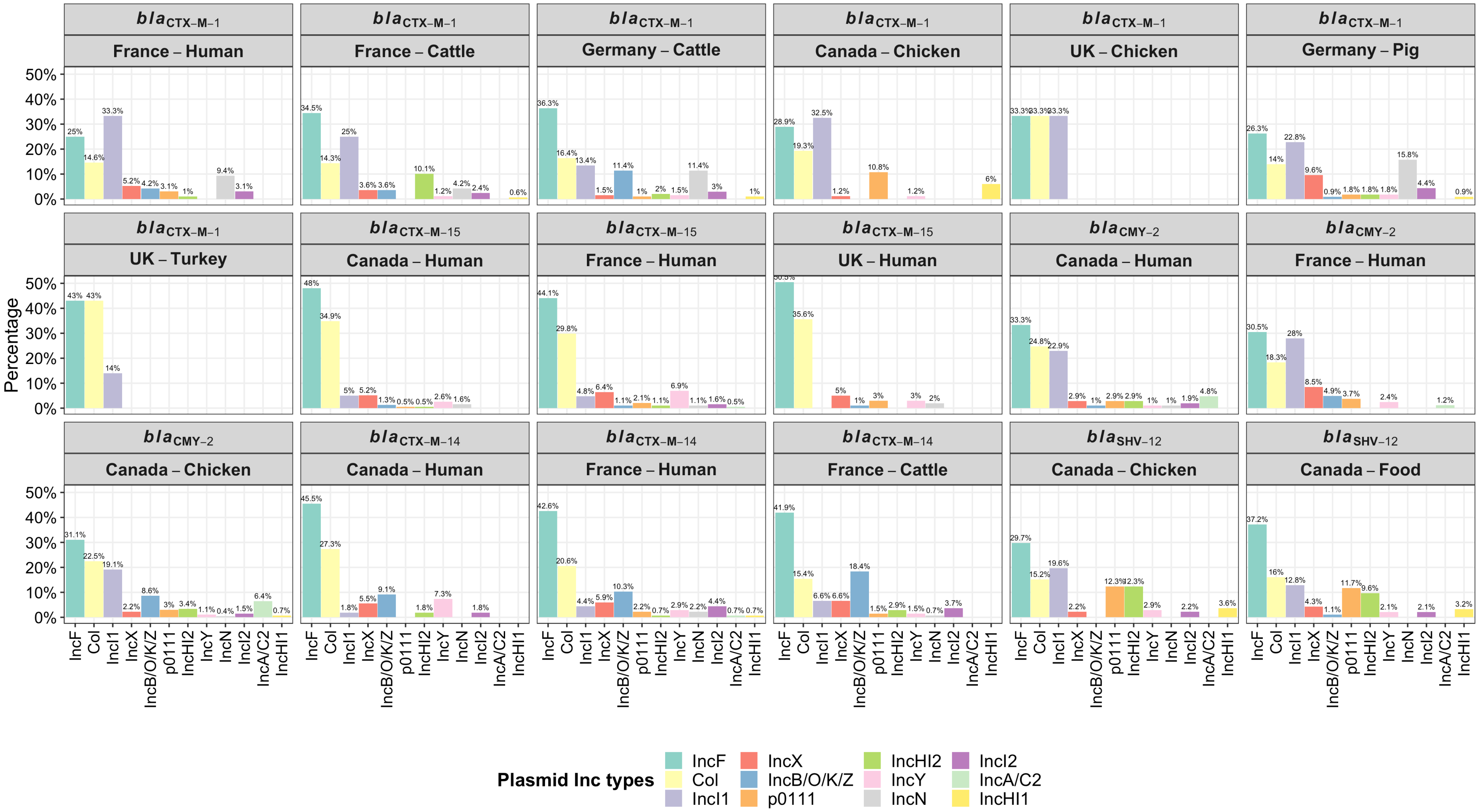

Figure S9. Percentage of main plasmid incompatibility (Inc) types based on replicons by compartments (country + source) for genomes carrying each major ESC-R gene. Bars are coloured by plasmid Inc types (as per inset legend).

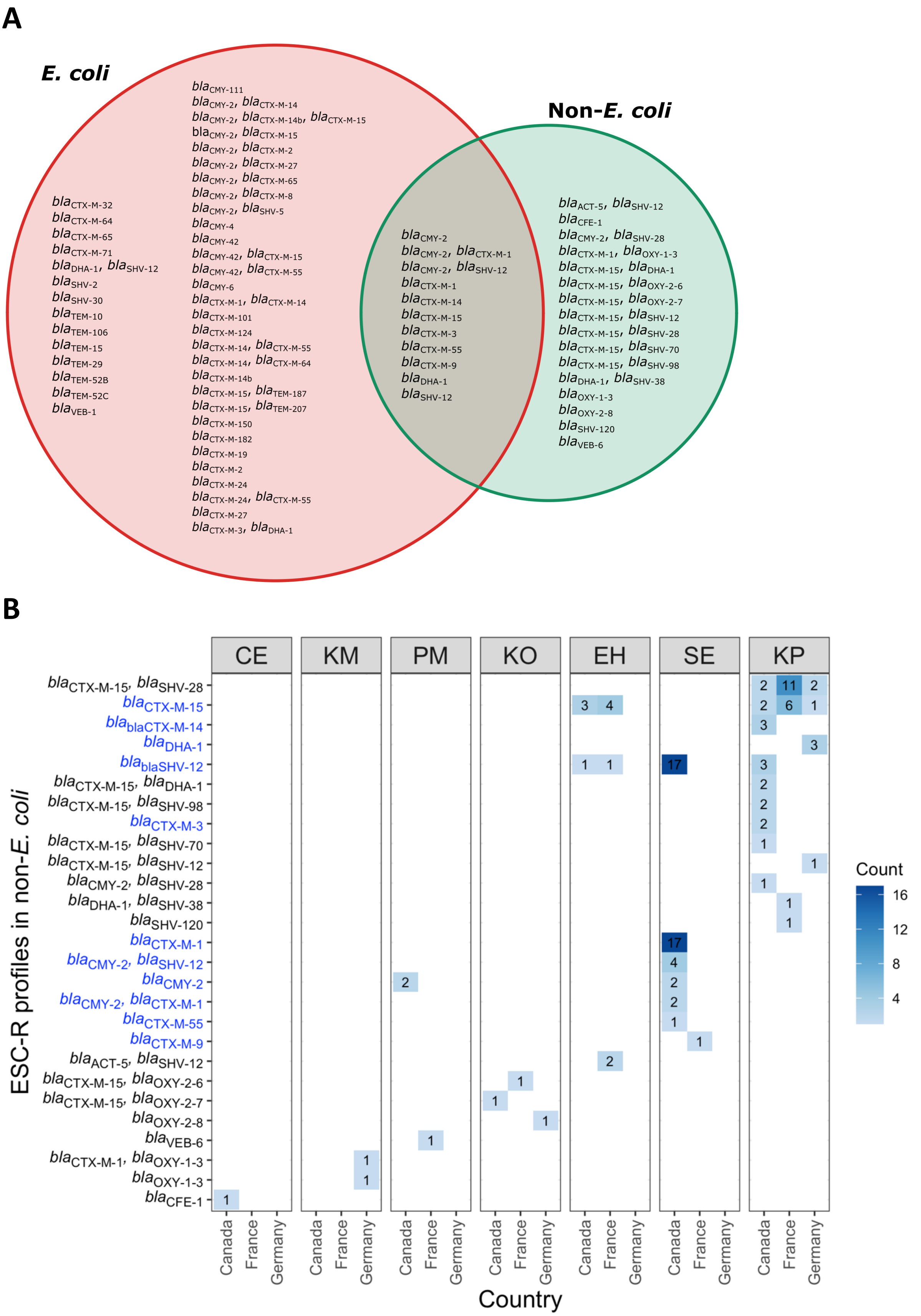

Figure S10. Comparison of ESC-R profiles between *E. coli* and non-*E. coli* isolates. (A) Venn diagram of shared and unique ESC-R profiles between *E. coli* and other Enterobacterales species. **(B)** Distribution by country of ESC-R profiles in non-*E. coli* isolates. KP: *Klebsiella*

pneumoniae; SE: *Salmonella enterica*; EH: *Enterobacter hormaechei*; KO: *Klebsiella oxytoca*; PM: *Proteus mirabilis*; KM: *Klebsiella michiganensis*; CE: *Citrobacter europaeus*. Blue text in the y-axis represents ESC-R profiles also found in *E. coli* genomes in this study.

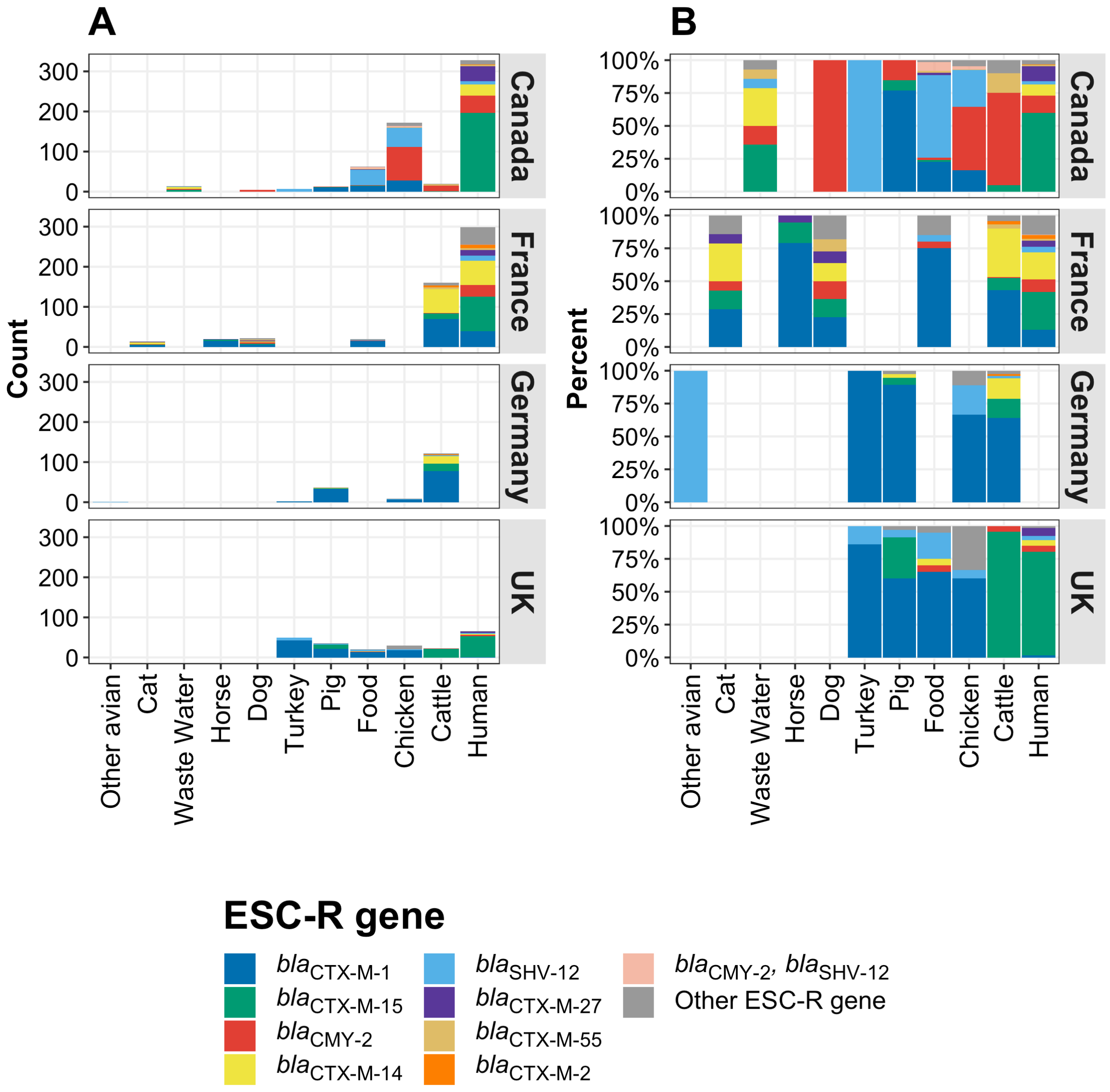

Figure S11. Frequency of ESC-R genes by compartment. (A) Count and **(B)** percentage of ESC-R genes by compartment (country + source). In the German collection, genomes from Pietsch et al 2018[12] were not included due to study design. Bars are coloured by ESC-R gene. ESC: extended-spectrum cephalosporin.

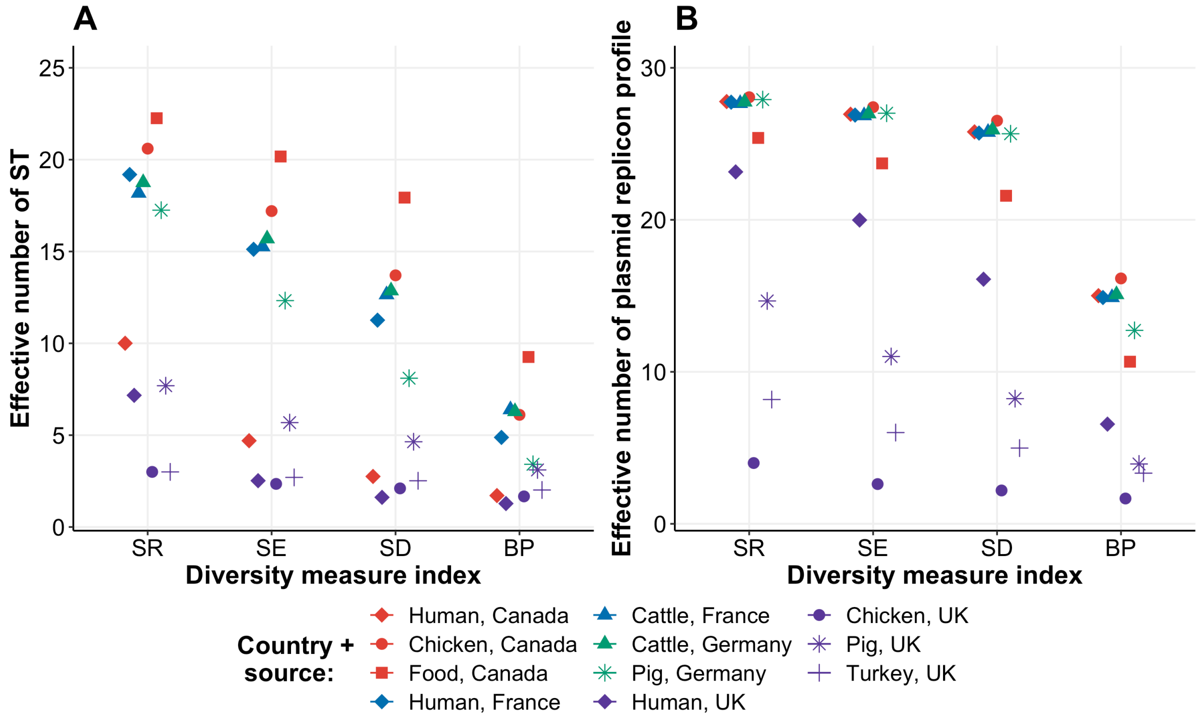

Figure S12. Diversity measures for ST and plasmid replicon profiles by compartment, plotted as effective numbers of profiles. Diversity indices for **(A)** ST and **(B)** plasmid replicon profiles by compartment (country + source), for compartments with minimum sample size of n=30. The shape of the dots is linked with the source and the colours with the country.

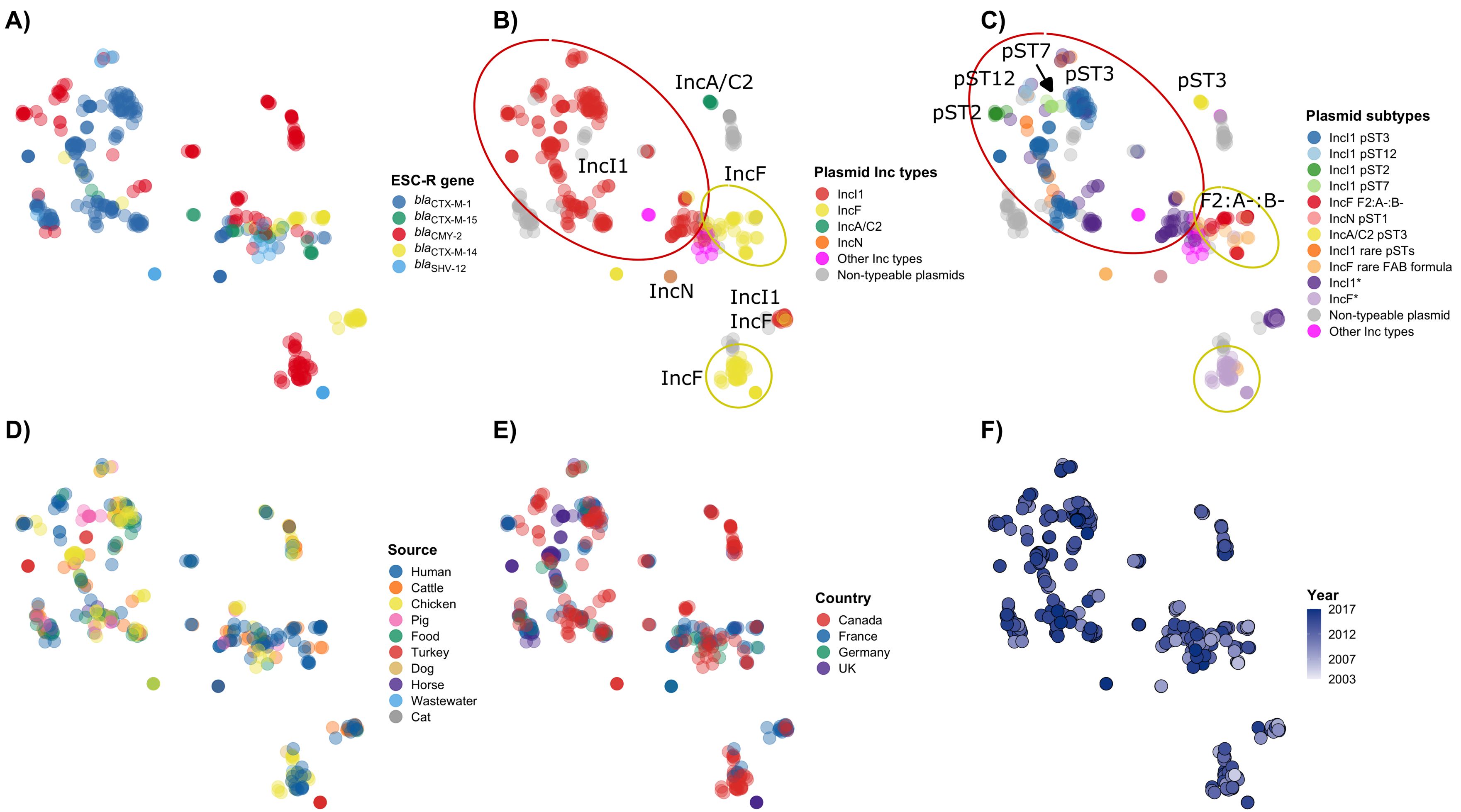

Figure S13. Gene content (plasmidome) network for 313 typeable ESC-R plasmids (136 *bla*$_{CTX-M-1}$, 23 *bla*$_{CTX-M-15}$, 88 *bla*$_{CMY-2}$, 40 *bla*$_{CTX-M-14}$ and 26 *bla*$_{SHV-12}$) plus 79 non-typeable plasmids recovered from short-read data. Each dot represents a plasmid, each of which is coloured by: **(A)** ESC-R gene, **(B)** plasmid Inc. type, **(C)** plasmid subtype, **(D)** source, **(E)** country, and **(F)** year. Clusters of plasmids belonging to the same major plasmid Inc types are circled and annotated. IncI1* and IncF* are those plasmids where the sequences for subtyping were not found in the same contig. ESC: extended-spectrum cephalosporin.

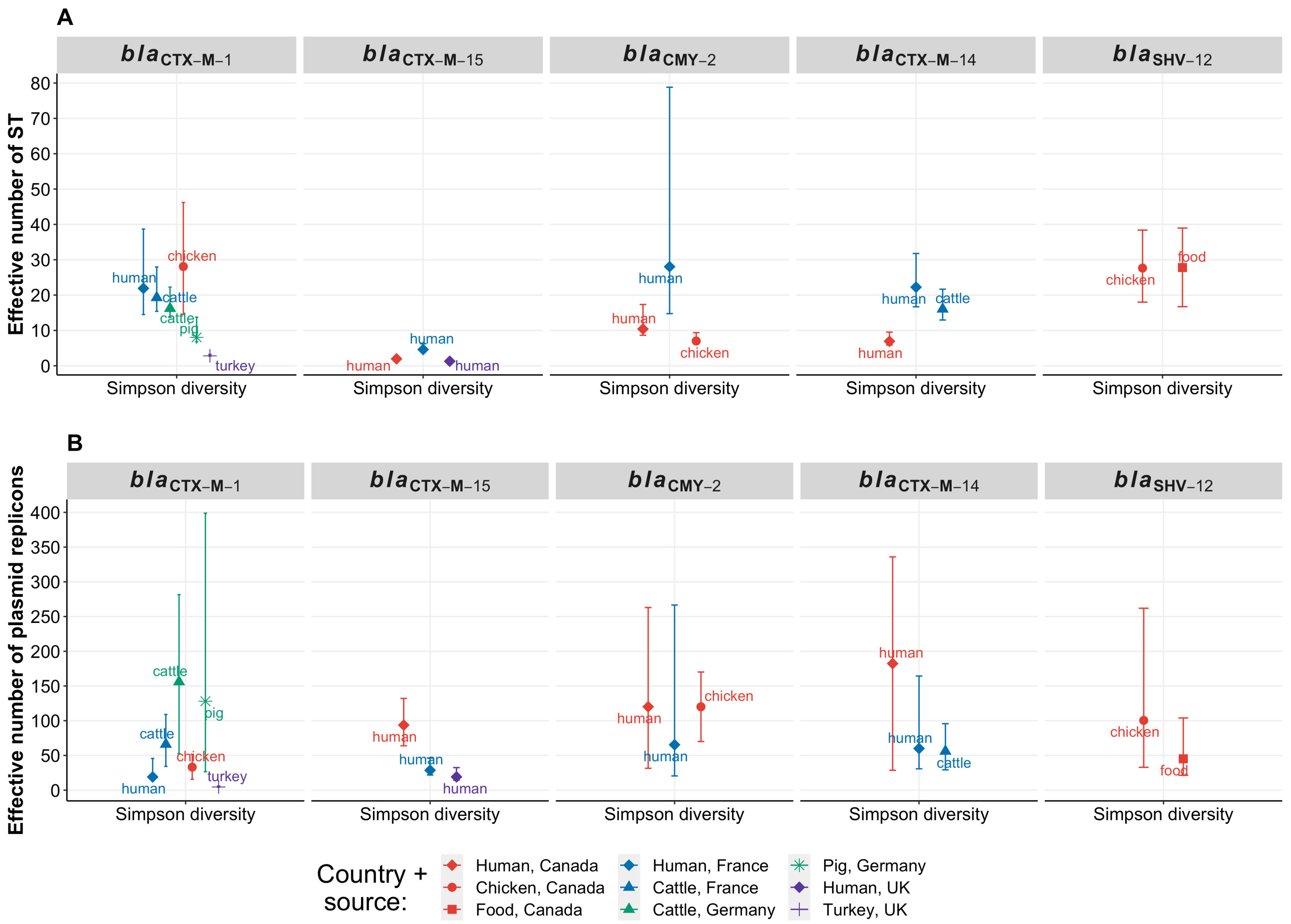

Figure S14. Simpson diversity estimate for ST and plasmid replicon profiles by compartments within genomes (n=1,014) carrying each major ESC-R gene. (A) ST and **(B)** plasmid replicons. Effective number of ST and plasmid replicon profiles are plotted along with 95% confidence intervals (CIs). The dots represent the estimate of Simpson's diversity and the vertical line represent the 95% CIs (coloured as per inset legend). ESC: extended-spectrum cephalosporin.

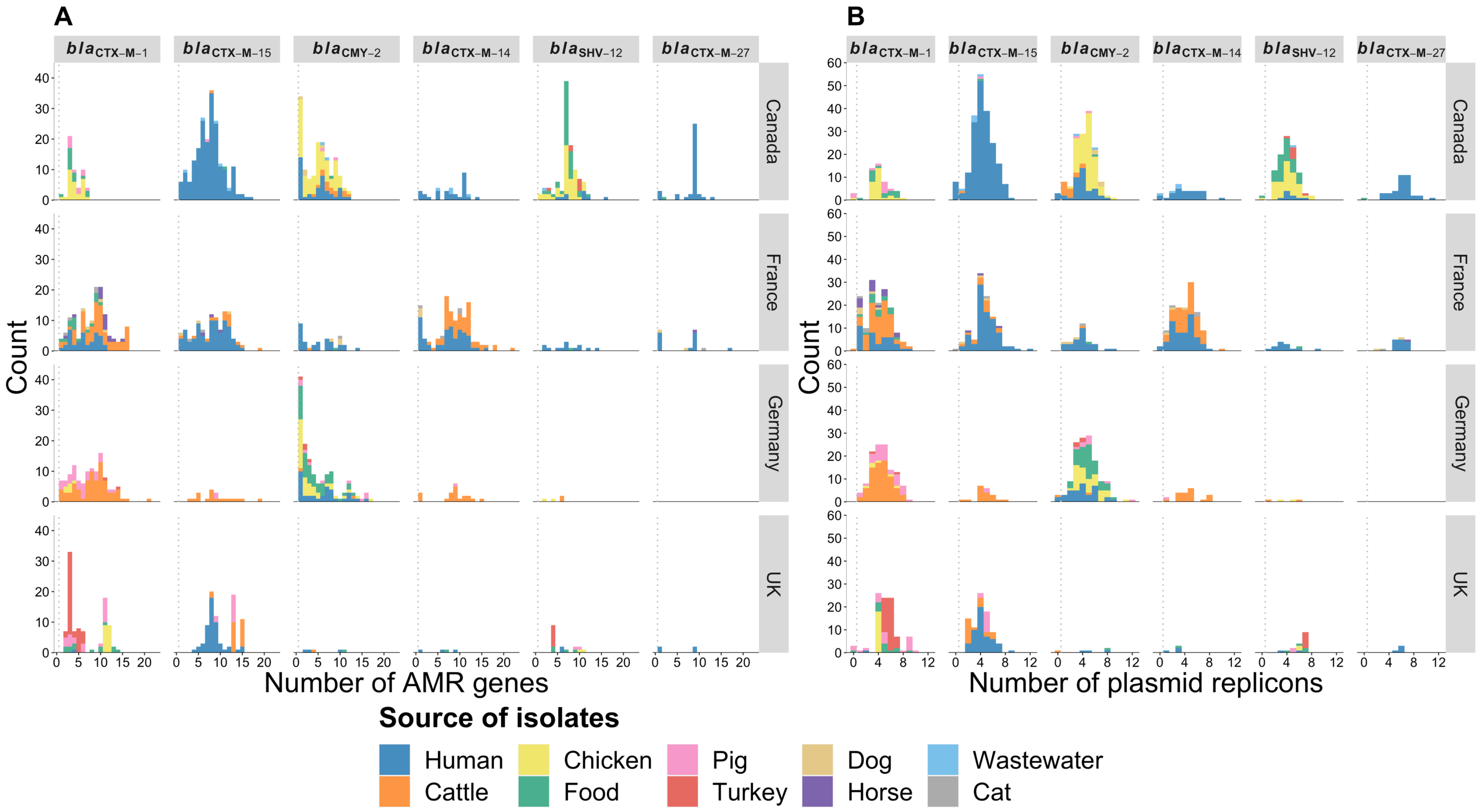

Figure S15. Distribution of the numbers of acquired AMR genes and plasmid replicons per genome by compartment. (A) Count of the numbers of acquired AMR genes and **(B)** plasmid replicons for genomes carrying the main ESC-R genes by compartment (country + source). Bar colour represents source. ESC: extended-spectrum cephalosporin.

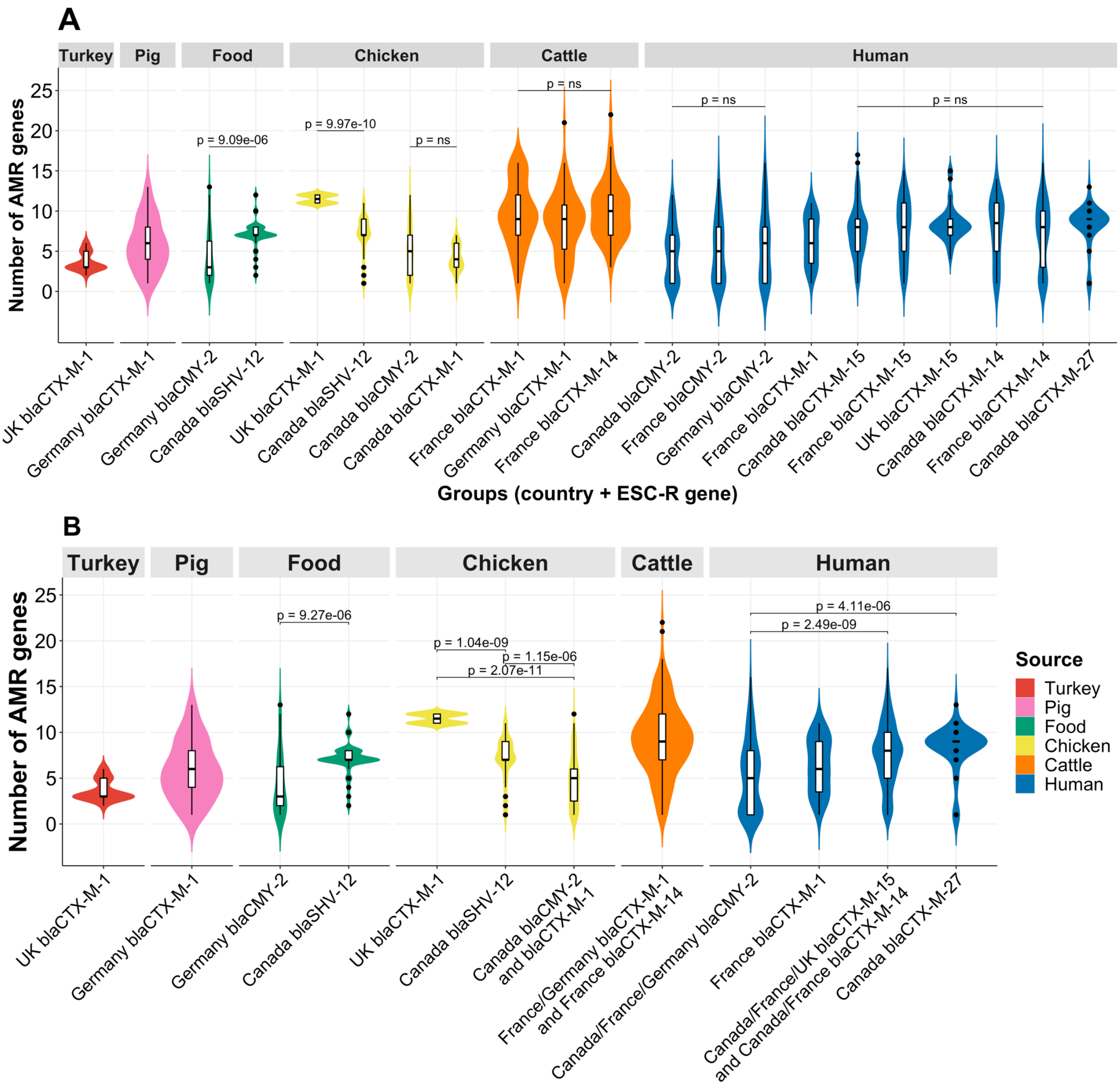

Figure S16. Distribution of the numbers of acquired AMR genes per genome between groups (country + ESC-R genes) for n=1,153 *E. coli* isolates. A) Number of AMR genes among groups of isolates per source; significance of comparisons shown by p-values of two-sided Kruskal-Wallis tests. ns: not significant. **B)** Number of AMR genes among groups per source. Significant two-sided Mann-Whitney U-test p-values < 0.005 shown. Colour represents source. For each boxplot, the length of the box corresponds to the interquartile range with the centre line corresponding to the median (50th percentile), the boundaries of the box represent the 25th percentile and 75th percentile, and the whiskers represent the minimum and maximum values, and the black circles represent outside values.

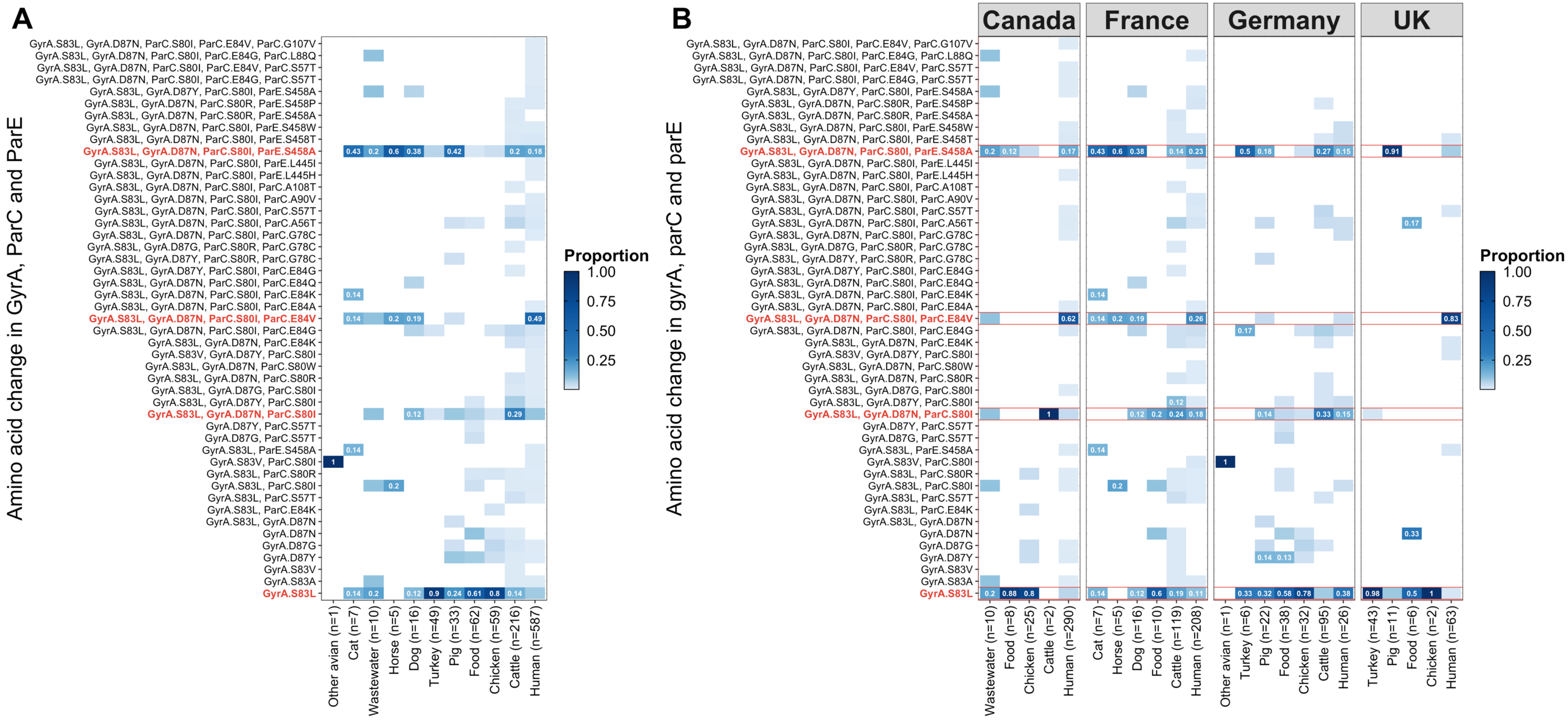

Figure S17. Distribution of substitutions in topoisomerase subunits associated with quinolone-fluoroquinolone resistance in *E. coli* genomes. Proportions of substitutions by **(A)** source and **(B)** compartment (country + source). Intensity of colour represents proportions and the numbers in the squares are for proportions more than 0.1 (10%). The most frequent substitutions were highlighted with red text. Heatmaps are coloured as per inset scale bars.

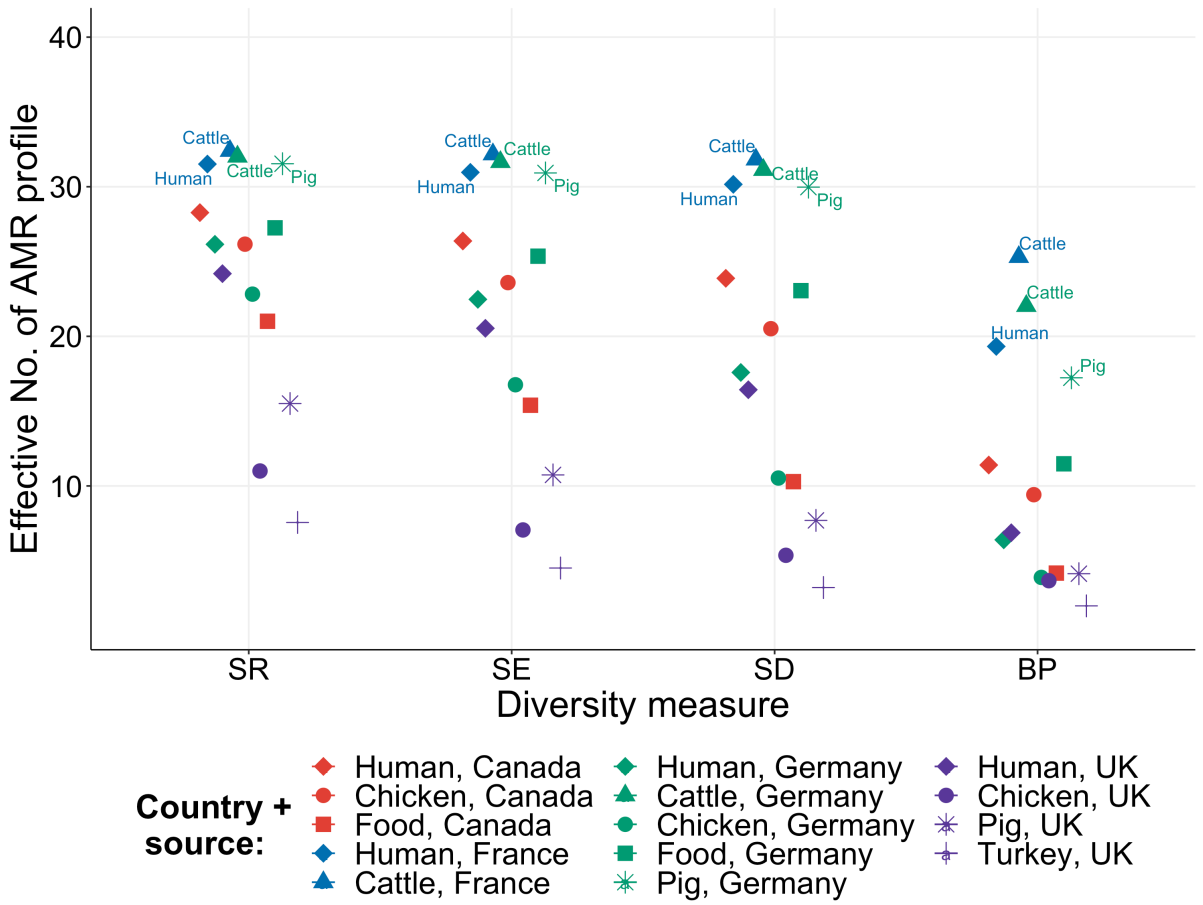

Figure S18. Diversity measures for AMR profiles by compartment, plotted as effective numbers of profiles. Diversity indices (SR: species richness; SE: Shannon entropy; SD: Simpson diversity; and reciprocal BP: Berger Parker) for AMR profiles by compartment (country + source), for compartments with minimum sample size of n=33. The shape of the dots is linked with the source and the colours with the country.

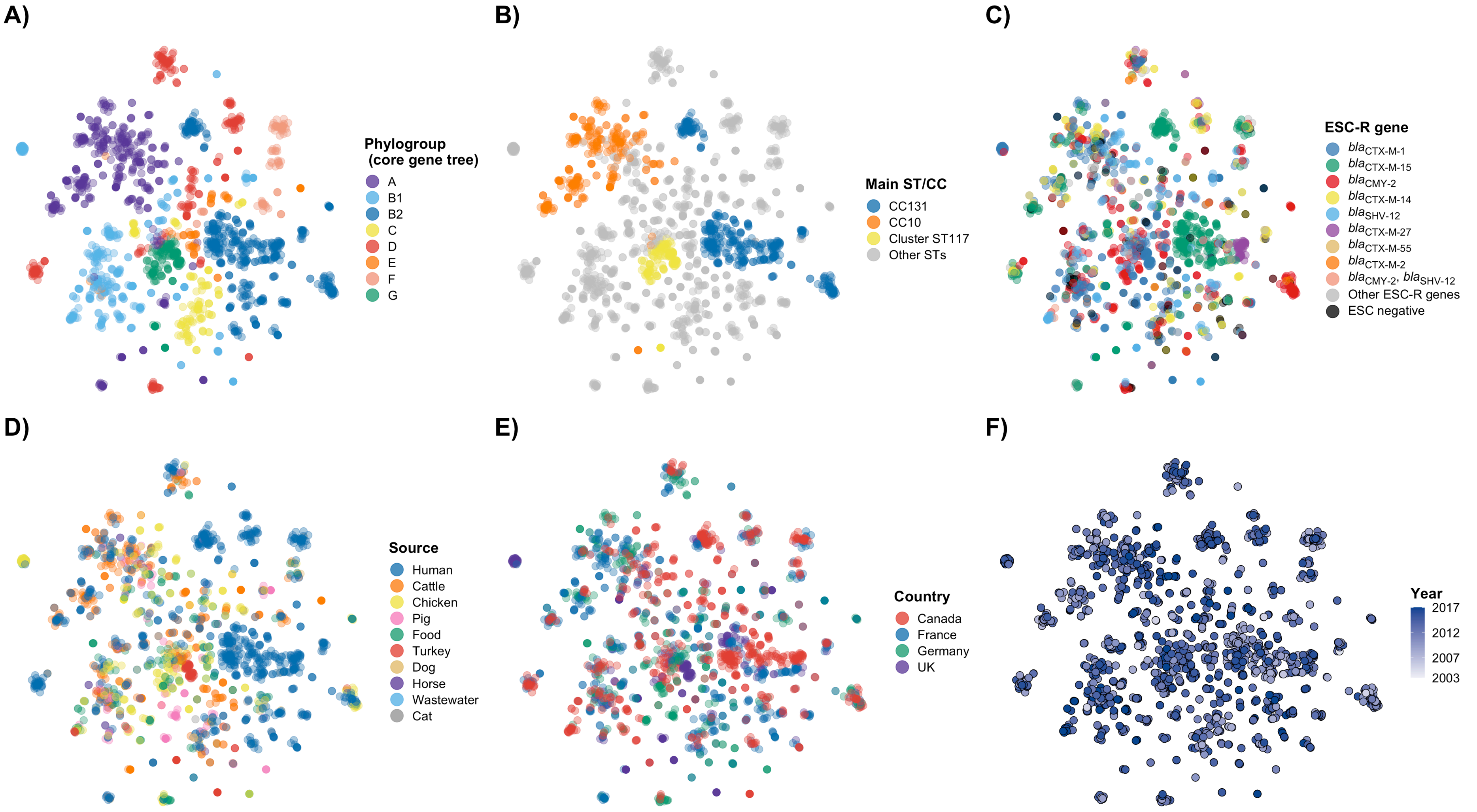

Figure S19. Gene content network for 1,818 *E. coli* genomes. Each dot represents an isolate, each of which is coloured by: **(A)** phylogroup (according to clusters defined by the core gene tree), **(B)** main ST/CC, **(C)** ESC-R gene, **(D)** source of the genome, **(E)** country of the sample, and **(F)** year of sample collection. ESC: extended-spectrum cephalosporin.

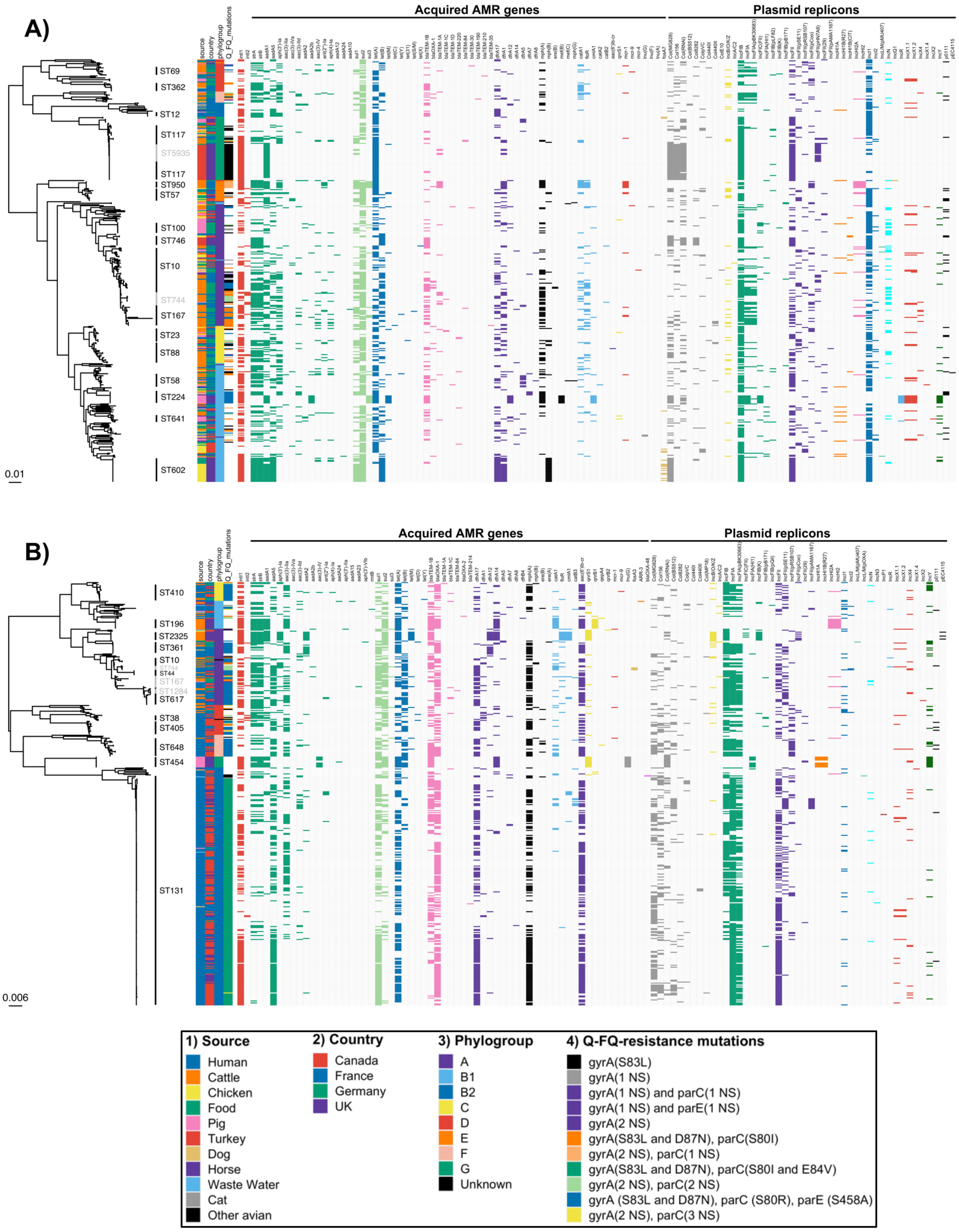

Figure S20. Core-gene phylogenetic trees for *E. coli* genomes with *bla*$_{CTX-M-1}$ and *bla*$_{CTX-M-15}$. Maximum likelihood tree for genomes carrying **(A)** *bla*$_{CTX-M-1}$ (n=420 isolates) and **(B)** *bla*$_{CTX-M-15}$ (n=427). STs with more than four genomes are labelled beside the tree. Source, country, phylogroup and Q-FQ-resistance mutations are coloured as shown in the inset legend. Q-FQ: quinolone-fluoroquinolone due to mutations in *gyrA*, *parC* and *parE*. NS: non-synonymous mutation.

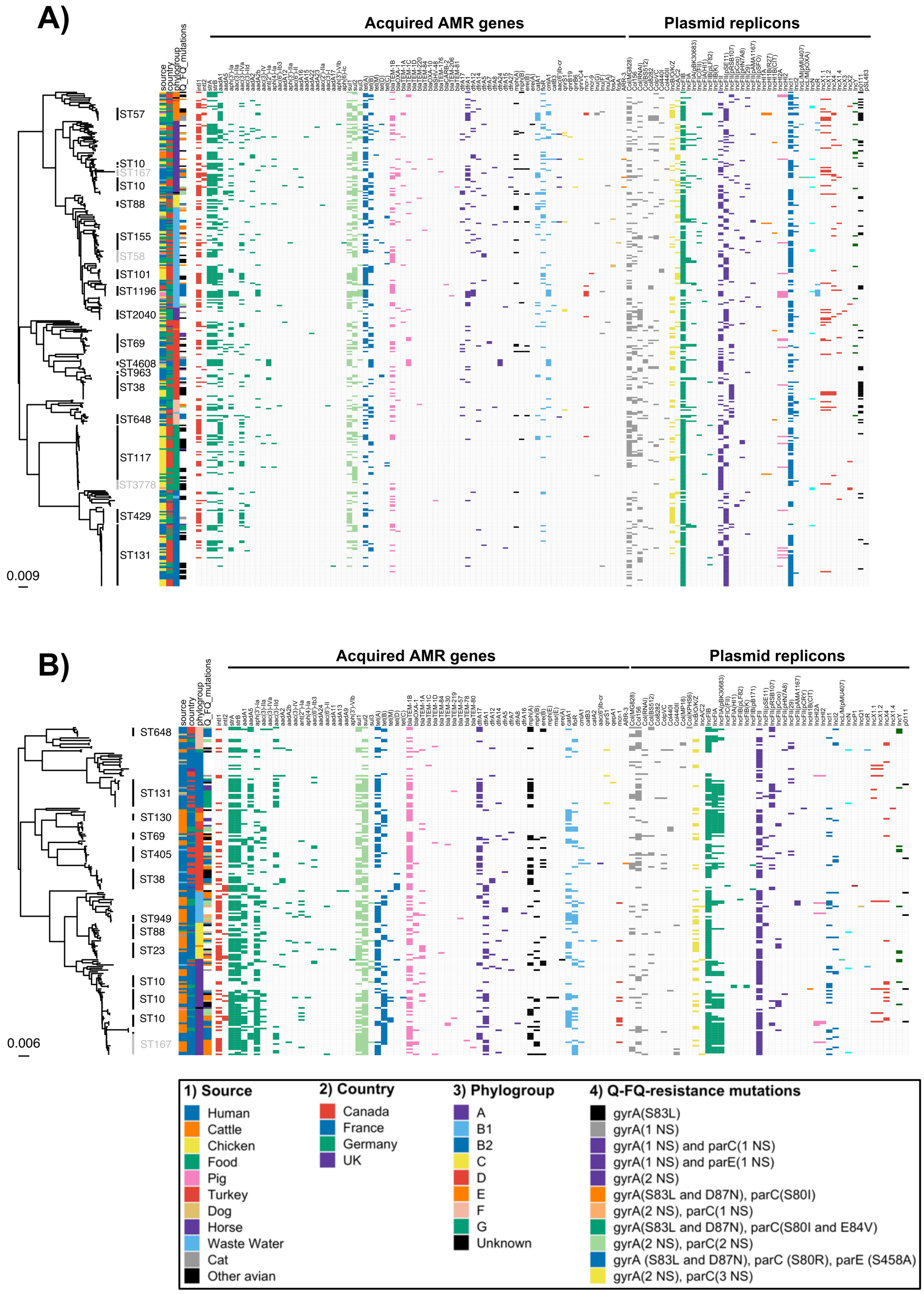

Figure S21. Core-gene phylogenetic trees for *E. coli* genomes with *bla*$_{CMY-2}$ and *bla*$_{CTX-M-14}$. Maximum likelihood tree for genomes with **(A)** *bla*$_{CMY-2}$ (n=355 isolates) and **(B)** *bla*$_{CTX-M-14}$ (n=191 isolates). STs with more than four genomes are labelled beside the tree. Source, country, phylogroup and Q-FQ-resistance mutations are coloured as shown in the inset legend. Q-FQ: quinolone-fluoroquinolone due to mutations in *gyrA*, *parC* and *parE*. NS: non-synonymous mutation.

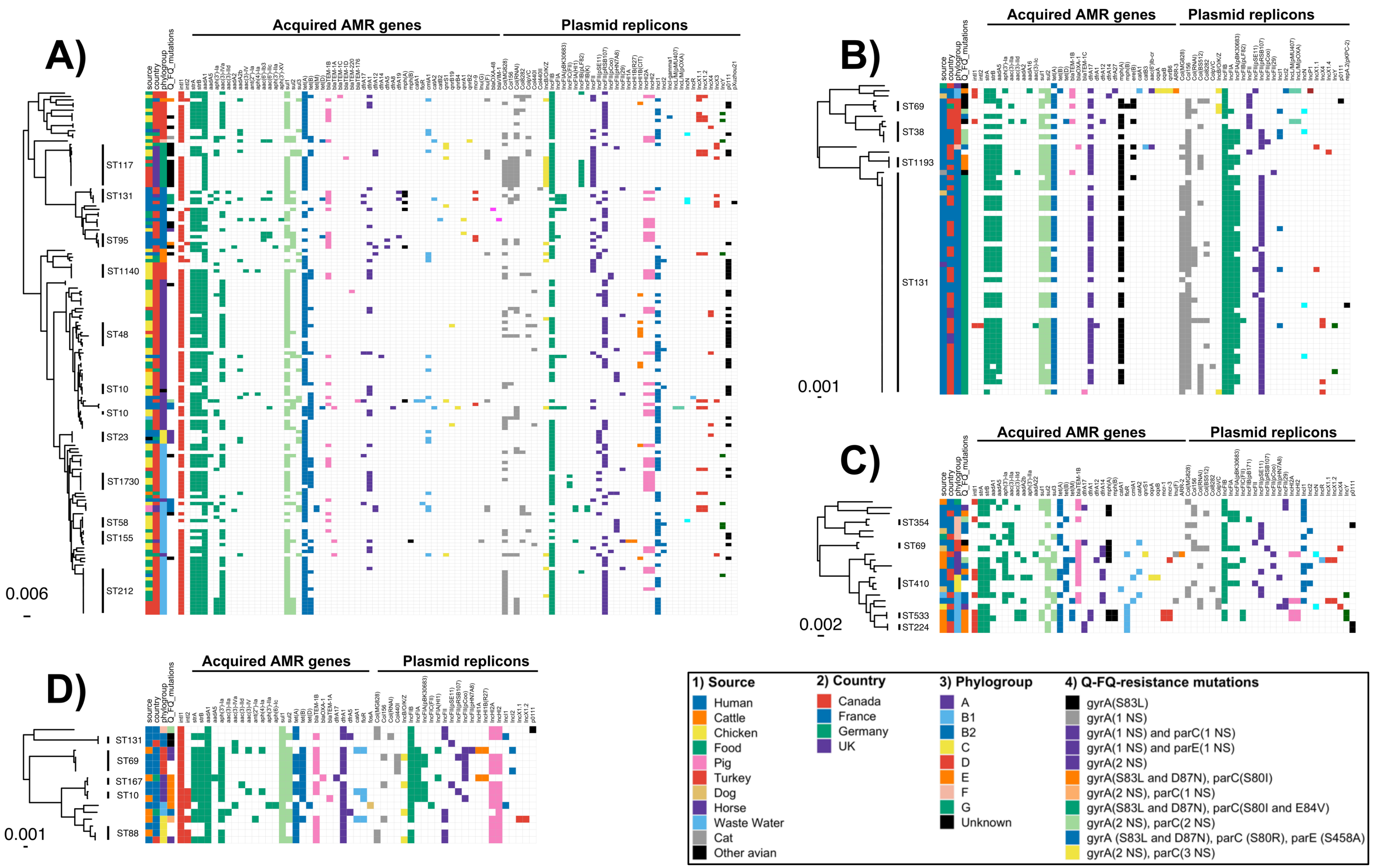

Figure S22. Core-gene phylogenetic trees for genomes with *bla*$_{\text{SHV-12}}$, *bla*$_{\text{CTX-M-27}}$, *bla*$_{\text{CTX-M-55}}$ and *bla*$_{\text{CTX-M-2}}$. Maximum likelihood tree for genomes carrying **(A)** *bla*$_{\text{SHV-12}}$ (n=153 isolates), **(B)** *bla*$_{\text{CTX-M-27}}$ (n=61 isolates), **(C)** *bla*$_{\text{CTX-M-55}}$ (n=23 isolates) and **(D)** *bla*$_{\text{CTX-M-2}}$ (n=17 isolates). STs with more than four genomes are labelled beside the tree. Source, country, phylogroup and Q-FQ-resistance mutations are coloured as shown in the inset legend. Q-FQ: quinolone-fluoroquinolone due to mutations in *gyrA*, *parC* and *parE*. NS: non-synonymous mutation.

SUPPLEMENTAL TABLES

Table S1. Bacterial isolates included in this study.

Country	Collaborators/other studies*	sample size	Reference
Canada	Mike R. Mulvey, George G. Zhanel	568	this study
	Patrick Boerlin, Gabhan Chalmers, Ashley Cormier	150	this study
France	Marisa Haenni, Jean-Yves Madec, Raquel Garcia Fierro	267	this study
	Richard Bonnet, Racha Beyrouthy	340	this study
Germany	Stefan Schwarz, Inga Eichhorn, Heike Kaspar	199	this study
	Pietsch et al., 2018[12]*	158	Doi: 10.1186/s12864-018-4976-3
UK	Kallonen et al., 2017[13]*	74	Doi:10.1101/gr.216606.116
	Ludden et al., 2019[14]*	174	Doi: 10.1128/mBio.02693-18
**TOTAL**		**1930**	

* Isolates from previous studies

Table S2. Sample size and percentage for each enteric bacterial species analysed in this study.

	Sample size	
**Enterobacterales**	**Count**	**Percentage**
Escherichia coli	1818	94.20
Klebsiella pneumoniae	46	2.38
Salmonella enterica	44	2.28
Enterobacter hormaechei	11	0.57
Klebsiella oxytoca	3	0.16
Proteus mirabilis	3	0.16
Klebsiella michiganensis	2	0.10
Citrobacter europaeus	1	0.05
Citrobacter freundii	1	0.05
Raoultella ornithinolytica	1	0.05
**TOTAL**	**1930**	**100**

Table S3. Count and percentage of *E. coli* and non-*E. coli* isolates in each ecological compartment constituted by country + source.

			E. coli isolates		
**Country**	**Canada**	**France**	**Germany**	**UK**	**TOTAL**
**Source**	**count (%)**	**count (%)**	**count (%)**	**count (%)**	**count (%)**
**Human**	340 (52.6%)	324 (56.3%)	43 (12.4%)	74 (29.8%)	781 (43.0%)
**Cattle**	22 (3.4%)	177 (30.7%)	138 (39.8%)	30 (12.1%)	367 (20.2%)
**Chicken**	177 (27.4%)	ND	49 (14.1%)	33 (13.3%)	259 (14.2%)
**Food**	64 (9.9%)	20 (3.5%)	60 (17.3%)	20 (8.1%)	164 (9.0%)
**Pig**	14 (2.2%)	ND	50 (14.4%)	38 (15.3%)	102 (5.6%)
**Turkey**	8 (1.2%)	ND	6 (1.7%)	53 (21.4%)	67 (3.7%)
**Dog**	6 (0.9%)	22 (3.8%)	ND	ND	28 (1.5%)
**Horse**	ND	19 (3.3%)	ND	ND	19 (1.0%)
**Wastewater**	16 (2.5%)	ND	ND	ND	16 (0.9%)
**Cat**	ND	14 (2.4%)	ND	ND	14 (0.8%)
**Other avian**	ND	ND	1 (0.3%)	ND	1 (0.1%)
**TOTAL**	647 (100%)	576 (100%)	347 (100%)	248 (100%)	1818 (100%)

Non-E. coli isolates					
**Country**	**Canada**	**France**	**Germany**	**UK**	**TOTAL**
**Source**	**count (%)**	**count (%)**	**count (%)**	**count (%)**	**count (%)**
**Human**	20 (28.2%)	16 (51.6%)	ND	ND	36 (32.1%)
**Cattle**	4 (5.6%)	1 (3.2%)	ND	ND	5 (4.5%)
**Chicken**	4 (5.6%)	ND	ND	ND	4 (3.6%)
**Food**	6 (8.5%)	ND	ND	ND	6 (5.4%)
**Pig**	12 (16.9%)	ND	ND	ND	12 (10.7%)
**Turkey**	17 (23.9%)	ND	ND	ND	17 (15.2%)
**Dog**	6 (8.5%)	8 (25.8%)	7 (70%)	ND	21 (18.8%)
**Horse**	1 (1.4%)	2 (6.5%)	1 (10%)	ND	4 (3.6%)
**Cat**	1 (1.4%)	4 (12.9%)	1 (10%)	ND	6 (5.4%)
**Other avian**	ND	ND	1 (10%)	ND	1 (0.9%)
**TOTAL**	71 (100%)	31 (100%)	10 (100%)	ND	112 (100%)

ND: no data

Table S4. Count and percentage of plasmid incompatibility (Inc.) type in *E. coli* collection.

Plasmid Inc. type	Count	Percentage
IncF[a]	1611	35.67
Col[b]	1010	22.36
IncI1	664	14.70
IncX[c]	253	5.60
IncB/O/K/Z	220	4.87
p0111	157	3.48
IncHI2[d]	130	2.88
IncY	127	2.79
IncN[e]	105	2.33
IncI2	81	1.79
IncA/C2	52	1.15
IncHI1[f]	52	1.15
IncR	30	0.66
IncL/M	10	0.22
IncP[g]	5	0.11
pEC4115	4	0.09
IncQ	3	0.07
repA	2	0.04
pSL483	1	0.02

[a]FIA(pBK30683), FII(pBK30683), IncFIA(HI1), IncFIA, IncFIB(AP001918), IncFIB(K), IncFIB(Mar), IncFIB(pB171), IncFIB(pECLA), IncFIB(pHCM2), IncFIB(pKPHS1), IncFIB(pLF82), IncFIB(pQil), IncFIC(FII), IncFII(29), IncFII(K), IncFII(pAMA1167-NDM-5), IncFII(pCoo), IncFII(pCRY), IncFII(pHN7A8), IncFII(pMET), IncFII(pRSB107), IncFII(pSE11), IncFII(Yp), IncFII, IncFII(pKP91) and IncFII(pSFO).

[b]Col(BS512), Col(KPHS6), Col(MG828), Col(MP18), Col156, Col440I, Col440II, Col8282, ColE10, ColpVC and ColRNAI.

[c]IncX1, IncX2, IncX3 and IncX4.

[d]IncHI2 and IncHI2A.

[e]IncN and IncN3.

[f]IncHI1A, IncHI1B, IncHI1B_CIT and IncHI1B_R27.

[g]IncP1 and IncP6.

Table S5. Numbers and percentage (within brackets) of ESC-resistance genes by compartment (country + source) in *E. coli* isolates.

Country	Source	Sample size	*bla*$_{CTX-M-1}$ n=414 (26.7%)	*bla*$_{CTX-M-15}$ n=418 (27.0%)	*bla*$_{CMY-2}$ n=188 (12.1%)	*bla*$_{CTX-M-14}$ n=183 (11.8%)	*bla*$_{SHV-12}$ n=140 (9.0%)	*bla*$_{CTX-M-27}$ n=60 (3.9%)	*bla*$_{CTX-M-55}$ n=20 (1.3%)	*bla*$_{CTX-M-2}$ n=16 (1.0%)	Other ESC n=109 (7.0%)
**Canada**	**Human**	**328**	-	**197 (60.1)**	**42 (12.8)**	**28 (8.5)**	9 (2.7)	37 (11.3)	3 (0.9)	2 (0.6)	10 (3.0)
	**Cattle**	20	-	1 (5.0)	14 (70.0)	-	-	-	3 (15.0)	-	2 (10.0)
	**Chicken**	**172**	**28 (16.3)**	-	**83 (48.3)**	-	**48 (27.9)**	-	1 (0.6)	-	12 (7.0)
	**Food**	62	14 (22.6)	1 (1.6)	1 (1.6)	-	**39 (62.9)**	1 (1.6)	1 (1.6)	-	5 (8.1)
	**Pig**	13	10 (76.9)	1 (7.7)	2 (15.4)	-	-	-	-	-	-
	**Turkey**	7	-	-	-	-	7 (100.0)	-	-	-	-
	**Dog**	4	-	-	4 (100.0)	-	-	-	-	-	-
	**Wastewater**	14	-	5 (35.7)	2 (14.3)	4 (28.6)	1 (7.1)	-	1 (7.1)	-	1 (7.1)
**France**	**Human**	**299**	**39 (13.0)**	**86 (28.8)**	**29 (9.7)**	**61 (20.4)**	13 (4.3)	14 (4.7)	4 (1.3)	8 (2.7)	45 (15.1)
	**Cattle**	**160**	**69 (43.1)**	15 (9.4)	1 (0.6)	**59 (36.9)**	-	-	5 (3.1)	4 (2.5)	7 (4.4)
	**Food**	20	15 (75.0)	-	1 (5.0)	-	1 (5.0)	-	-	-	3 (15.0)
	**Dog**	22	5 (22.7)	3 (13.6)	3 (13.6)	3 (13.6)	-	2 (9.1)	2 (9.1)	-	4 (18.2)
	**Horse**	19	15 (78.9)	3 (15.8)	-	-	-	1 (5.3)	-	-	-
	**Cat**	14	4 (28.6)	2 (14.3)	1 (7.1)	4 (28.6)	-	1 (7.1)	-	-	2 (14.3)
**Germany***	**Cattle**	**122**	**78 (63.9)**	18 (14.8)	-	**19 (15.6)**	2 (1.6)	-	-	2 (1.6)	3 (2.5)
	**Chicken**	9	6 (66.7)	-	-	-	2 (22.2)	-	-	-	1 (11.1)
	**Pig**	**37**	**33 (89.2)**	2 (5.4)	-	1 (2.7)	-	-	-	-	1 (2.7)
	**Turkey**	2	2 (100.0)	-	-	-	-	-	-	-	-
	**Other avian**	1	-	-	-	-	1 (100.0)	-	-	-	-
**UK**	**Human**	66	1 (1.5)	**52 (78.8)**	3 (4.5)	3 (4.5)	2 (3.0)	4 (6.1)	-	-	1 (1.5)
	**Cattle**	22	-	21 (95.5)	1 (4.5)	-	-	-	-	-	-
	**Chicken**	**30**	**18 (60.0)**	-	-	-	2 (6.7)	-	-	-	10 (33.3)
	**Food**	20	13 (65.0)	-	1 (5.0)	1 (5.0)	4 (20.0)	-	-	-	1 (5.0)
	**Pig**	35	21 (60.0)	11 (31.4)	-	-	2 (5.7)	-	-	-	1 (2.9)
	**Turkey**	**50**	**43 (86.0)**	-	-	-	7 (14.0)	-	-	-	-

*German genomes from Pietsch et al 2018[12], which focused on *bla*$_{CMY-2}$, were not included due to study design.

Table S6. Fisher's Exact Test for the comparison of proportions among main compartments within each major ESC-R genes.

ESC-R gene	Total n	Compartment 1 (n)	Compartment 1 (%)	Compartment 2 (n)	Compartment 2 (%)	p-value[1]	Adjusted p-value[2]
bla$_{CTX-M-1}$	471	Canada - Chicken (n=172)	16.3	France - Human (n=299)	13.0	0.340	1.000
	219	Canada - Chicken (n=172)	16.3	UK - Chicken (n=30)	60.0	1.59E-6	4.93E-05*
	332	France - Cattle (n=160)	43.1	Canada - Chicken (n=172)	16.3	7.91E-08	2.45E-06*
	459	France - Cattle (n=160)	43.1	France - Human (n=299)	13.0	2.24E-12	6.94E-11*
	282	France - Cattle (n=160)	43.1	Germany - Cattle (n=122)	63.9	7.27E-04	0.023*
	207	France - Cattle (n=160)	43.1	UK - Chicken (n=30)	60.0	0.111	1.000
	346	France - Human (n=299)	12.2	UK - Chicken (n=47)	63.8	6.14E-13	1.90E-11*
	294	Germany - Cattle (n=122)	63.9	Canada - Chicken (n=172)	16.3	4.76E-17	1.48E-15*
	421	Germany - Cattle (n=122)	63.9	France - Human (n=299)	13.0	9.57E-25	2.97E-23*
	169	Germany - Cattle (n=122)	63.9	UK - Chicken (n=30)	60.0	0.679	1.000
bla$_{CTX-M-15}$	627	Canada - Human (n=328)	60.1	France - Human (n=299)	28.8	2.42E-15	7.50E-14*
	488	Canada - Human (n=328)	60.1	France - Cattle (n=160)	9.4	4.14E-29	1.28E-27*
	450	Canada - Human (n=328)	60.1	Germany - Cattle (n=122)	14.8	8.72E-19	2.70E-17*
	394	Canada - Human (n=328)	60.1	UK - Human (n=66)	78.8	0.005	0.155
	459	France - Human (n=299)	28.8	France - Cattle (n=160)	9.4	8.82E-07	2.73E-05*
	421	France - Human (n=299)	28.8	Germany - Cattle (n=122)	14.8	0.003	0.093
	365	France - Human (n=299)	28.8	UK - Human (n=66)	78.8	8.41E-14	2.61E-12*
	282	Germany - Cattle (n=122)	14.8	France - Cattle (n=160)	9.4	0.192	1.000
	226	UK - Human (n=66)	78.8	France - Cattle (n=160)	9.4	1.35E-24	4.19E-23*
	188	UK - Human (n=66)	78.8	Germany - Cattle (n=122)	14.8	4.65E-18	1.44E-16*
bla$_{CMY-2}$	500	Canada - Chicken (n=172)	48.3	Canada - Human (n=328)	12.8	2.39E-17	7.41E-16*
	471	Canada - Chicken (n=172)	48.3	France - Human (n=299)	9.7	1.02E-20	3.16E-19*
	627	Canada - Human (n=328)	12.8	France - Human (n=299)	9.7	0.256	1.000
bla$_{CTX-M-14}$	627	France - Human (n=299)	20.4	Canada - Human (n=328)	8.5	3.01E-05	0.001*
	421	France - Human (n=299)	20.4	Germany - Cattle (n=122)	15.6	0.276	1.000
	488	France - Cattle (n=160)	36.9	Canada - Human (n=328)	8.5	1.33E-13	4.12E-12*
	459	France - Cattle (n=160)	36. 9	France - Human (n=299)	20.4	2.15E-04	0.007*
	282	France - Cattle (n=160)	36. 9	Germany - Cattle (n=122)	15. 6	8.50E-05	0.003*
	450	Germany - Cattle (n=122)	15. 6	Canada - Human (n=328)	8.5	0.037	1.000
bla$_{SHV-12}$	219	Canada - Chicken (n=172)	27.9	UK - Chicken (n=30)	6.7	0.011	0.347
bla$_{CTX-M-27}$	627	Canada - Human (n=328)	11.3	France - Human (n=299)	4.7	0.003	0.093

[1]p-values are from two-sided Fisher's Exact Test. [2]p-values were adjusted for multiple comparisons with the Bonferroni method.
*Statistically significant p-value Bonferroni corrected <0.05. These significant comparisons are also highlighted in grey.

Table S7. Proportion of ESC-R genes located on plasmid and chromosome contigs (predicted by MOB-suite alone and MOB-suite plus RFPlasmid tools).

ESC-R genes	Location	MOB-suite		MOB-suite + RFPlasmid*	
		n (%)	n (%)	n (%)	n (%)
bla$_{CTX-M-1}$ (n=412)	Chromosome contig	240 (58.3)	240 (58.3)	60 (14.6)	60 (14.6)
	Typeable plasmid contigs	136 (33.0)	172 (41.7)	136 (33)	352 (85.4)
	Non-typeable plasmid contig	36 (8.7)		216 (52.4)	
bla$_{CTX-M-15}$ (n=418)	Chromosome contig	394 (94.3)	394 (94.3)	84 (20.1)	84 (20.1)
	Typeable plasmid contigs	23 (5.5)	24 (5.7)	23 (5.5)	334 (79.9)
	Non-typeable plasmid contig	1 (0.2)		311 (74.4)	
bla$_{CMY-2}$ (n=188)	Chromosome contig	63 (33.5)	63 (33.5)	8 (4.3)	8 (4.3)
	Typeable plasmid contigs	88 (46.8)	125 (66.5)	88 (46.8)	180 (95.7)[a]
	Non-typeable plasmid contig	37 (19.7)		92 (48.9)	
bla$_{CTX-M-14}$ (n=178)	Chromosome contig	134 (75.3)	134 (75.3)	39 (21.9)	39 (21.9)
	Typeable plasmid contigs	40 (22.5)	44 (24.7)	40 (22.5)	139 (78.1)[b]
	Non-typeable plasmid contig	4 (2.2)		99 (55.6)	
bla$_{SHV-12}$ (n=140)	Chromosome contig	114 (81.4)	114 (81.4)	9 (6.4)	9 (6.4)
	Typeable plasmid contigs	25 (17.9)	26 (18.6)	26 (18.6)	131 (93.6)
	Non-typeable plasmid contig	1 (0.7)		105 (75)	

*Plasmid and chromosome origin by MOB-suite and RFPlasmid consist of: i) chromosome category as inferred by both MOB-suite and RFPlasmid, ii) plasmid category includes typeable plasmids (presence of plasmid replicon genes) and non-typeable plasmids (absence of plasmid replicon genes); the typeable plasmid (ESC-R plasmids) category included plasmid prediction by both MOB-suite and RFPlasmid; the non-typeable plasmid category included also those classified as plasmids by RFPlasmid but as chromosome by MOB-suite.

[a]*bla*$_{CMY-2}$: in three genomes the ESC-R gene location was predicted as chromosome by RFPlasmid but as plasmid by MOB-suite. Inspection of these contigs identified replicon sequences, therefore they were included within the plasmid category.

[b]*bla*$_{CTX-M-14}$: one chromosome inference by RFPlasmid but predicted as plasmid by MOB-suite. This contig also carried a replicon sequence, therefore it was included within the plasmid category.

Table S8. Proportion of plasmid types in the main ESC-R gene dataset and in the typeable plasmids subset by ESC-R genes in *E. coli*.

		Main ESC-R gene dataset (n=1,343)		Typeable plasmid subset (n=313)	
**ESC-R gene**	**Plasmid type**	**Count**	**%**	**Count**	**%**
*bla*CTX-M-1	IncF	355	31.84	5	3.68
	IncI1	266	23.86	124	91.18
	Col*	216	19.37	-	-
	IncN	60	5.38	4	2.94
	IncX	45	4.04	-	-
	IncB/O/K/Z	42	3.77	-	-
	p0111	29	2.6	-	-
	IncY	26	2.33	-	-
	IncHI2	24	2.15	-	-
	IncHI1	19	1.7	-	-
	IncI2	19	1.7	-	-
	IncR	9	0.81	-	-
	IncL/M	3	0.27	-	-
	IncQ	1	0.09	-	-
	pEC4115	1	0.09	-	-
	Other**	-	-	3	2.21
*bla*CTX-M-15	IncF	383	44.64	9	39.13
	Col*	261	30.42	2	8.7
	IncY	45	5.24	-	-
	IncX	44	5.13	1	4.35
	IncI1	38	4.43	7	30.43
	IncB/O/K/Z	24	2.8	-	-
	IncHI2	17	1.98	-	-
	IncN	13	1.52	-	-
	IncHI1	9	1.05	-	-
	p0111	9	1.05	-	-
	IncI2	5	0.58	-	-
	IncA/C2	3	0.35	-	-
	pEC4115	3	0.35	-	-
	IncR	2	0.23	-	-
	IncL/M	1	0.12	-	-
	IncP	1	0.12	-	-
	rep	-	-	3	13.04
	IncI2	-	-	1	4.35
*bla*CMY-2	IncF	168	31.23	35	39.77
	Col*	113	21.00	1	1.14
	IncI1	113	21.00	41	46.59
	IncA/C2	36	6.69	7	7.95
	IncB/O/K/Z	31	5.76	-	-
	IncX	19	3.53	2	2.28
	p0111	18	3.35	-	-
	IncHI2	12	2.23	1	1.14

	IncI2	9	1.67	-	-
	IncY	9	1.67	-	-
	IncHI1	3	0.56	-	-
	IncN	3	0.56	-	-
	IncR	3	0.56	-	-
	pSL483	1	0.19	-	-
	Other**	-	-	1	1.14
bla$_{CTX-M-14}$	IncF	173	44.13	21	52.5
	Col*	77	19.64	-	-
	IncB/O/K/Z	49	12.5	-	-
	IncI1	23	5.87	19	47.5
	IncX	22	5.61	-	-
	IncI2	13	3.32	-	-
	IncY	12	3.06	-	-
	IncHI2	7	1.79	-	-
	IncN	5	1.28	-	-
	p0111	5	1.28	-	-
	IncR	2	0.51	-	-
	IncA/C2	1	0.26	-	-
	IncHI1	1	0.26	-	-
	IncP	1	0.26	-	-
	IncQ	1	0.26	-	-
bla$_{SHV-12}$	IncF	122	32.11	14	53.85
	Col*	66	17.37	2	7.69
	IncI1	62	16.32	6	23.08
	IncHI2	35	9.21	1	3.85
	p0111	35	9.21	-	-
	IncX	22	5.79	1	3.85
	IncB/O/K/Z	12	3.16	-	-
	IncHI1	10	2.63	-	-
	IncY	7	1.84	-	-
	IncI2	5	1.32	-	-
	IncN	2	0.53	-	-
	IncR	1	0.26	-	-
	pXuzhou21	1	0.26	-	-
	rep	-	-	1	3.85
	Other**	-	-	1	3.85

*Col(BS512), Col(KPHS6), Col(MG828), Col(MP18), Col156, Col440I, Col440II, Col8282, ColE10, ColpVC and ColRNAI.
**Cases where two distinct replicons were found in the same plasmid (i.e., ColRNAI+IncI1, IncF+IncI1, IncI1+rep, rep+IncH).
The main plasmid types are highlighted in the table and the dash "-" indicate of the absence of the data.

Table S9. Summary of logistic regression analysis for the trend of ESC-R types/genes over a 12-year period by compartment in *E. coli*.

By ESC-R type

Country	Source	ESC-R types	Occurrence#	Estimate	OR [95% CI]	p-value	Adjusted p-value[1]
Canada	Human	AmpC	43	-0.120	0.89 [0.79-0.99]	0.035	0.175
Canada	Human	ESBL	283	0.120	1.13 [1.01-1.26]	0.035	0.175
Canada	Farm animals	AmpC	99	-0.998	0.37 [0.28-0.47]	1.97E-14	9.85E-14*
Canada	Farm animals	ESBL	106	0.998	2.71 [2.14-3.58]	1.97E-14	9.85E-14*
France	Human	AmpC	33	0.417	1.52 [1.29-1.82]	2.38E-06	1.19E-06*
France	Human	ESBL	251	-0.417	0.66 [0.55-0.78]	2.38E-06	1.19E-06*
France	Farm animals	ESBL	157	0.187	1.21 [0.54-2.63]	0.585	1.000
France	Companion animals	ESBL	51	-0.174	0.84 [0.45-1.37]	0.524	1.000

By ESC-R gene

Country	Source	ESC-R genes	Occurrence#	Estimate	OR [95% CI]	p-value	Adjusted p-value
Canada	Human	*bla*$_{CTX\text{-}M\text{-}15}$	197	-0.031	0.97 [0.90-1.05]	0.427	1.000
Canada	Human	*bla*$_{CMY\text{-}2}$	42	-0.127	0.88 [0.79-0.99]	0.027	0.459
Canada	Human	*bla*$_{CTX\text{-}M\text{-}14}$	28	-0.031	0.97 [0.85-1.11]	0.650	1.000
Canada	Human	*bla*$_{CTX\text{-}M\text{-}27}$	37	0.201	1.22 [1.07-1.41]	0.004	0.068
France	Human	*bla*$_{CTX\text{-}M\text{-}1}$	39	-0.047	0.95 [0.83-1.09]	0.495	1.000
France	Human	*bla*$_{CTX\text{-}M\text{-}15}$	86	-0.147	0.86 [0.78-0.96]	0.006	0.102
France	Human	*bla*$_{CMY\text{-}2}$	25	0.420	1.52 [1.26-1.89]	3.82E-05	6.49E-04*
France	Human	*bla*$_{CTX\text{-}M\text{-}14}$	61	0.010	1.01 [0.90-1.13]	0.863	1.000
UK	Human	*bla*$_{CTX\text{-}M\text{-}15}$	52	-0.268	0.76 [0.51-1.11]	0.174	1.000
Canada	Chicken	*bla*$_{CTX\text{-}M\text{-}1}$	28	0.198	1.22 [1.03-1.48]	0.031	0.527
Canada	Chicken	*bla*$_{CMY\text{-}2}$	83	-1.181	0.31 [0.21-0.41]	2.71E-12	4.61E-11*
France	Cattle	*bla*$_{CTX\text{-}M\text{-}1}$	68	-0.063	0.94 [0.83-1.06]	0.303	1.000
France	Cattle	*bla*$_{CTX\text{-}M\text{-}15}$	15	-0.052	0.95 [0.78-1.17]	0.604	1.000
France	Cattle	*bla*$_{CTX\text{-}M\text{-}14}$	58	0.116	1.12 [0.99-1.29]	0.083	1.000
Germany	Cattle	*bla*$_{CTX\text{-}M\text{-}1}$	78	-0.219	0.80 [0.68-0.94]	0.008	0.136
Germany	Cattle	*bla*$_{CTX\text{-}M\text{-}15}$	18	0.096	1.10 [0.90-1.37]	0.365	1.000
Germany	Cattle	*bla*$_{CTX\text{-}M\text{-}14}$	19	0.280	1.32 [1.06-1.70]	0.017	0.289

[1]p-values were adjusted for multiple comparisons with the Bonferroni method.
#Occurrence for each ESC-R gene by compartment (country + source).
*Statistically significant p-value Bonferroni corrected <0.05

Table S10. Proportion of resistance to each AMR class by compartment (country and source). Bold text represents main compartments that were further analysed with Fisher's Exact Test between compartments within each main drug class (See the statistical testing in Table S11).

Country	Source	Sample size	Aminoglycosides % (n)	Sulfonamides % (n)	Tetracyclines % (n)	Q-FQ (mutations)* % (n)	Beta-lactams % (n)	Diaminopyrimidines % (n)	Macrolides % (n)	Phenicols % (n)	Aminoglycoside-FQ % (n)	Quinolone-FQ** % (n)	Colistin % (n)	Lincosamides % (n)	Fosfomycins % (n)	Rifampicin % (n)	Carbapenem % (n)
**Canada**	**Human**	**340**	**82.1 (n=279)**	**72.4 (n=246)**	**67.9 (n=231)**	**85.3 (n=290)**	**62.9 (n=214)**	**65.9 (n=224)**	**55.6 (n=189)**	**13.5 (n=46)**	39.7 (n=135)	2.9 (n=10)	0.9 (n=3)	0.3 (n=1)	0 (n=0)	0.3 (n=1)	0 (n=0)
**France**	**Human**	**324**	**72.5 (n=235)**	**67.6 (n=219)**	**66.7 (n=216)**	**64.2 (n=208)**	**65.7 (n=213)**	**56.5 (n=183)**	**30.6 (n=99)**	**24.1 (n=78)**	16.8 (n=58)	4.9 (n=16)	1.2 (n=4)	0.9 (n=3)	2.2 (n=7)	1.2 (n=4)	1.2 (n=4)
Germany	**Human**	43	72.1 (n=31)	67.4 (n=29)	51.2 (n=22)	60.5 (n=26)	58.1 (n=25)	44.2 (n=19)	18.6 (n=8)	34.9 (n=15)	14 (n=6)	7 (n=3)	0 (n=0)	0 (n=0)	0 (n=0)	7 (n=3)	0 (n=0)
UK	**Human**	74	91.9 (n=68)	86.5 (n=64)	55.4 (n=41)	85.1 (n=63)	87.8 (n=65)	82.4 (n=61)	63.5 (n=47)	13.5 (n=10)	64.9 (n=48)	0 (n=0)	0 (n=0)	2.7 (n=2)	0 (n=0)	0 (n=0)	0 (n=0)
Canada	**Cattle**	22	100 (n=22)	90.9 (n=20)	90.9 (n=20)	9.1 (n=2)	27.3 (n=6)	68.2 (n=15)	18.2 (n=4)	77.3 (n=17)	0 (n=0)	18.2 (n=4)	0 (n=0)	4.5 (n=1)	0 (n=0)	13.6 (n=3)	0 (n=0)
**France**	**Cattle**	**177**	**95.5 (n=169)**	**94.9 (n=168)**	**90.4 (n=160)**	**67.2 (n=119)**	**74.6 (n=132)**	**62.1 (n=110)**	**31.1 (n=55)**	**57.1 (n=101)**	2.8 (n=5)	1.1 (n=2)	19.2 (n=34)	0.6 (n=1)	0 (n=0)	0 (n=0)	0 (n=0)
**Germany**	**Cattle**	**138**	**80.4 (n=111)**	**74.6 (n=103)**	**73.9 (n=102)**	**68.8 (n=95)**	**57.2 (n=79)**	**42.8 (n=59)**	**41.3 (n=57)**	**54.3 (n=75)**	9.4 (n=13)	1.4 (n=2)	4.3 (n=6)	0 (n=0)	0 (n=0)	0 (n=0)	0 (n=0)
UK	**Cattle**	30	73.3 (n=22)	73.3 (n=22)	73.3 (n=22)	0 (n=0)	76.7 (n=23)	70 (n=21)	0 (n=0)	63.3 (n=19)	33.3 (n=10)	70 (n=21)	0 (n=0)	0 (n=0)	0 (n=0)	0 (n=0)	0 (n=0)
**Canada**	**Chicken**	**177**	**72.3 (n=128)**	**74.6 (n=132)**	**73.4 (n=130)**	**14.1 (n=25)**	**16.4 (n=29)**	**11.3 (n=20)**	**0.6 (n=1)**	**10.2 (n=18)**	0 (n=0)	1.1 (n=2)	0.6 (n=1)	1.7 (n=3)	2.3 (n=4)	0 (n=0)	0 (n=0)
Germany	**Chicken**	49	34.7 (n=17)	44.9 (n=22)	32.7 (n=16)	65.3 (n=32)	30.6 (n=15)	20.4 (n=10)	6.1 (n=3)	14.3 (n=7)	0 (n=0)	2 (n=1)	6.1 (n=3)	6.1 (n=3)	0 (n=0)	0 (n=0)	0 (n=0)
**UK**	**Chicken**	**33**	**97 (n=32)**	**66.7 (n=22)**	**97 (n=32)**	**6.1 (n=2)**	**36.4 (n=12)**	**63.6 (n=21)**	**54.5 (n=18)**	**3 (n=1)**	0 (n=0)	0 (n=0)	0 (n=0)	45.5 (n=15)	27.3 (n=9)	0 (n=0)	0 (n=0)
Canada	Food	64	81.2 (n=52)	93.8 (n=60)	93.8 (n=60)	12.5 (n=8)	14.1 (n=9)	23.4 (n=15)	1.6 (n=1)	4.7 (n=3)	0 (n=0)	6.2 (n=4)	0 (n=0)	0 (n=0)	4.7 (n=3)	0 (n=0)	0 (n=0)

France		20	65 (n=13)	75 (n=15)	60 (n=12)	50 (n=10)	20 (n=4)	60 (n=12)	10 (n=2)	10 (n=2)	0 (n=0)	0 (n=0)	0 (n=0)	5 (n=1)	0 (n=0)	0 (n=0)	0 (n=0)
Germany		60	48.3 (n=29)	50 (n=30)	41.7 (n=25)	63.3 (n=38)	36.7 (n=22)	21.7 (n=13)	5 (n=3)	20 (n=12)	0 (n=0)	6.7 (n=4)	5 (n=3)	3.3 (n=2)	1.7 (n=1)	0 (n=0)	0 (n=0)
UK		20	80 (n=16)	90 (n=18)	80 (n=16)	30 (n=6)	30 (n=6)	60 (n=12)	35 (n=7)	10 (n=2)	0 (n=0)	10 (n=2)	(n=0)	10 (n=2)	10 (n=2)	0 (n=0)	0 (n=0)
Canada	Pig	14	64.3 (n=9)	92.9 (n=13)	78.6 (n=11)	0 (n=0)	42.9 (n=6)	28.6 (n=4)	7.1 (n=1)	7.1 (n=1)	0 (n=0)	0 (n=0)	0 (n=0)	7.1 (n=1)	0 (n=0)	0 (n=0)	0 (n=0)
Germany		50	78 (n=39)	72 (n=36)	56 (n=28)	44 (n=22)	44 (n=22)	50 (n=25)	34 (n=17)	16 (n=8)	4 (n=2)	6 (n=3)	18 (n=9)	2 (n=1)	0 (n=0)	0 (n=0)	0 (n=0)
UK		38	81.6 (n=31)	81.6 (n=31)	94.7 (n=36)	28.9 (n=11)	60.5 (n=23)	89.5 (n=34)	23.7 (n=9)	34.2 (n=13)	0 (n=0)	31.6 (n=12)	0 (n=0)	34.2 (n=13)	0 (n=0)	0 (n=0)	0 (n=0)
Canada	Turkey	8	100 (n=8)	87.5 (n=7)	100 (n=8)	0 (n=0)	12.5 (n=1)	12.5 (n=1)	0 (n=0)	0 (n=0)	0 (n=0)	0 (n=0)	0 (n=0)	0 (n=0)	0 (n=0)	0 (n=0)	0 (n=0)
Germany		6	33.3 (n=2)	33.3 (n=2)	16.7 (n=1)	100 (n=6)	66.7 (n=4)	33.3 (n=2)	33.3 (n=2)	0 (n=0)	0 (n=0)	0 (n=0)	16.7 (n=1)	0 (n=0)	0 (n=0)	0 (n=0)	0 (n=0)
UK		53	94.3 (n=50)	22.6 (n=12)	100 (n=53)	81.1 (n=43)	32.1 (n=17)	9.4 (n=5)	0 (n=0)	0 (n=0)	0 (n=0)	0 (n=0)	0 (n=0)	0 (n=0)	0 (n=0)	0 (n=0)	0 (n=0)
France	Horse	19	100 (n=19)	89.5 (n=17)	89.5 (n=17)	26.3 (n=5)	36.8 (n=7)	84.2 (n=16)	57.9 (n=11)	52.6 (n=10)	5.3 (n=1)	5.3 (n=1)	0 (n=0)	0 (n=0)	0 (n=0)	5.3 (n=1)	0 (n=0)
Canada	Dog	6	16.7 (n=1)	33.3 (n=2)	66.7 (n=4)	0 (n=0)	0 (n=0)	0 (n=0)	0 (n=0)	0 (n=0)	0 (n=0)	0 (n=0)	0 (n=0)	0 (n=0)	0 (n=0)	0 (n=0)	0 (n=0)
France		22	72.7 (n=16)	59.1 (n=13)	63.6 (n=14)	72.7 (n=16)	54.5 (n=12)	40.9 (n=9)	13.6 (n=3)	31.8 (n=7)	9.1 (n=2)	0 (n=0)	4.5 (n=1)	0 (n=0)	0 (n=0)	0 (n=0)	0 (n=0)
Canada	Wastewater	16	81.2 (n=13)	68.8 (n=11)	75 (n=12)	62.5 (n=10)	37.5 (n=6)	68.8 (n=11)	37.5 (n=6)	25 (n=4)	12.5 (n=2)	18.8 (n=3)	0 (n=0)	0 (n=0)	0 (n=0)	0 (n=0)	0 (n=0)
France	Cat	14	64.3 (n=9)	64.3 (n=9)	71.4 (n=10)	50 (n=7)	64.3 (n=9)	50 (n=7)	42.9 (n=6)	14.3 (n=2)	7.1 (n=1)	0 (n=0)	0 (n=0)	0 (n=0)	0 (n=0)	0 (n=0)	0 (n=0)
Germany	Other avian	1	100 (n=1)	100 (n=1)	100 (n=1)	100 (n=1)	0 (n=0)	0 (n=0)	0 (n=0)	100 (n=1)	0 (n=0)	0 (n=0)	0 (n=0)	0 (n=0)	0 (n=0)	0 (n=0)	0 (n=0)
**Total**		**1818**	**78.2 (n=1422)**	**72.8 (n=1324)**	**71.5 (n=1300)**	**57.5 (n=1045)**	**52.9 (n=961)**	**50.0 (n=909)**	**30.2 (n=549)**	**24.9 (n=452)**	**15.4 (n=283)**	**5.0 (n=90)**	**3.5 (n=65)**	**2.7 (n=49)**	**1.4 (n=26)**	**0.7 (n=12)**	**0.2 (n=4)**

*Q-FQ (mutations); resistance to quinolone-fluoroquinolone due to mutations in *gyrA*, *parC* and *parE*.

**Quinolone-FQ; resistance to quinolone-fluoroquinolone due to acquired AMR genes.

Table S11. Results of Fisher's Exact Tests for the comparison of proportion of resistance between compartments within each main AMR class. Comparisons were evaluated using Fisher's Exact test available in the rstatix[15] R package. Only significant p-values Bonferroni corrected are considered in this table.

Antimicrobial class	Total	Compartment 1 (n)	Compartment 1 (%)	Compartment 2 (n)	Compartment 2 (%)	p-value[1]	Adjusted p-value[2]
Aminoglycosides	517	France - Cattle (n=177)	95.5	Canada - Human (n=340)	82.1	8.70E-06	0.0010
	522	France - Cattle (n=177)	95.5	France - Human (n=324)	72.5	2.20E-11	2.64E-09
	315	France - Cattle (n=177)	95.5	Germany - Cattle (n=138)	80.4	3.99E-05	0.0048
	354	France - Cattle (n=177)	95.5	Canada - Chicken (n=177)	72.3	1.68E-09	2.02E-07
Sulfonamides	517	France - Cattle (n=177)	94.9	Canada - Human (n=340)	72.4	5.95E-11	7.14E-09
	522	France - Cattle (n=177)	94.9	France - Human (n=324)	67.6	7.42E-14	8.90E-12
	315	France - Cattle (n=177)	94.9	Germany - Cattle (n=138)	74.6	3.25E-07	3.90E-05
	354	France - Cattle (n=177)	94.9	Canada - Chicken (n=177)	74.6	8.71E-08	1.05E-05
	210	France - Cattle (n=177)	94.9	UK - Chicken (n=33)	66.7	1.82E-05	0.0022
Tetracyclines	517	France - Cattle (n=177)	90.4	Canada - Human (n=340)	67.9	3.57E-09	4.28E-07
	522	France - Cattle (n=177)	90.4	France - Human (n=324)	66.7	8.65E-10	1.04E-07
	315	France - Cattle (n=177)	90.4	Germany - Cattle (n=138)	73.9	0.000129	0.0155
	345	France - Cattle (n=177)	90.4	Canada - Chicken (n=177)	73.4	4.85E-05	0.0058
	373	UK - Chicken (n=33)	97.0	Canada - Human (n=340)	67.9	0.0002	0.0221
	357	UK - Chicken (n=33)	97.0	France - Human (n=324)	66.7	9.03E-05	0.0108
Quinolones-fluoroquinolones (mutations)*	685	Canada - Human (n=340)	85.3	France - Human (n=324)	64.2	3.92E-10	4.70E-08
	517	Canada - Human (n=340)	85.3	France - Cattle (n=177)	67.2	3.88E-06	4.66E-04
	478	Canada - Human (n=340)	85.3	Germany - Cattle (n=138)	68.8	6.91E-05	0.0083
	517	Canada - Human (n=340)	85.3	Canada - Chicken (n=177)	14.1	8.15E-59	9.78E-57
	390	Canada - Human (n=340)	85.3	UK - Chicken (n=50)	12.0	2.77E-25	3.32E-23
	522	Canada - Chicken (n=177)	14.1	France - Human (n=324)	64.2	1.12E-28	1.34E-26
	354	Canada - Chicken (n=177)	14.1	France - Cattle (n=177)	67.2	3.08E-25	3.70E-23
	315	Canada - Chicken (n=177)	14.1	Germany - Cattle (n=138)	68.8	7.07E-24	8.48E-22
	357	UK - Chicken (n=33)	6.1	France - Human (n=324)	64.2	3.25E-11	3.90E-9
	210	UK - Chicken (n=33)	6.1	France - Cattle (n=177)	67.2	1.84E-11	2.21E-9
	171	UK - Chicken (n=33)	6.1	Germany - Cattle (n=138)	68.8	1.54E-11	1.85E-9
Beta-lactams	517	Canada - Chicken (n=177)	16.4	Canada - Human (n=340)	62.9	3.45E-25	4.14E-23
	522	Canada - Chicken (n=177)	16.4	France - Human (n=324)	65.7	1.65E-27	1.98E-25
	354	Canada - Chicken (n=177)	16.4	France - Cattle (n=177)	74.6	2.37E-29	2.84E-27
	315	Canada - Chicken (n=177)	16.4	Germany - Cattle (n=138)	57.2	3.27E-14	3.92E-12
	210	UK - Chicken (n=33)	36.4	France - Cattle (n=177)	74.6	5.36E-05	0.0064
Diaminopyrimidines	478	Canada - Human (n=340)	65.9	Germany - Cattle (n=138)	42.8	5.35E-06	6.42E-04

	517	Canada - Chicken (n=177)	11.3	Canada - Human (n=340)	65.9	5.98E-35	7.18E-33
	522	Canada - Chicken (n=177)	11.3	France - Human (n=324)	56.5	7.88E-25	9.46E-23
	354	Canada - Chicken (n=177)	11.3	France - Cattle (n=177)	62.1	3.05E-24	3.66E-22
	315	Canada - Chicken (n=177)	11.3	Germany - Cattle (n=138)	42.8	2.54E-10	3.05E-08
	210	Canada - Chicken (n=177)	11.3	UK - Chicken (n=33)	63.6	6.31E-10	7.57E-08
Macrolides	685	Canada - Human (n=340)	55.6	France - Human (n=324)	30.6	6.92E-11	8.30E-09
	517	Canada - Human (n=340)	55.6	France - Cattle (n=177)	31.1	1.08E-07	1.30E-05
	517	Canada - Human (n=340)	55.6	Canada - Chicken (n=177)	0.6	1.40E-44	1.68E-42
	522	Canada - Chicken (n=177)	0.6	France - Human (n=324)	30.6	2.41E-20	2.89E-18
	354	Canada - Chicken (n=177)	0.6	France - Cattle (n=177)	31.1	1.27E-17	1.52E-15
	315	Canada - Chicken (n=177)	0.6	Germany - Cattle (n=138)	41.3	4.18E-23	5.02E-21
	210	Canada - Chicken (n=177)	0.6	UK - Chicken (n=33)	54.5	3.92E-16	4.70E-14
Phenicols	522	France - Human (n=324)	24.1	Canada - Chicken (n=177)	10.2	2.91E-05	0.0035
	517	France - Cattle (n=177)	57.1	Canada - Human (n=340)	13.5	1.40E-24	1.68E-22
	522	France - Cattle (n=177)	57.1	France - Human (n=324)	24.1	4.74E-13	5.69E-11
	354	France - Cattle (n=177)	57.1	Canada - Chicken (n=177)	10.2	1.24E-21	1.49E-19
	210	France - Cattle (n=177)	57.1	UK - Chicken (n=33)	3.0	8.97E-10	1.08E-07
	478	Germany - Cattle (n=138)	54.3	Canada - Human (n=340)	13.5	3.28E-19	3.94E-17
	483	Germany - Cattle (n=138)	54.3	France - Human (n=324)	24.1	7.81E-10	9.37E-08
	315	Germany - Cattle (n=138)	54.3	Canada - Chicken (n=177)	10.2	6.10E-18	7.32E-16
	171	Germany - Cattle (n=138)	54.3	UK - Chicken (n=33)	3.0	8.38E-09	1.01E-06

[1]p-values are from two-sided Fisher's Exact Test. [2]p-values were adjusted for multiple comparisons with the Bonferroni method.
*Q-FQ (mutations); resistance to quinolone-fluoroquinolone due to mutations in *gyrA*, *parC* and *parE*.

Table S12. List and frequency of non-synonymous mutation in gyrase A and topoisomerase IV genes in *E. coli* (n=1,045). Count and percentage of the substitutions in topoisomerase subunits by compartments (country + source). The most frequent substitutions were highlighted in grey.

		**Canada (n=335)**					**France (n=365)**						**Germany (n=220)**							**UK (n=125)**				
	**Total n (%)**	**Human**	**Cattle**	**Chicken**	**Food**	**Wastewater**	**Human**	**Cattle**	**Food**	**Dog**	**Horse**	**Cat**	**Human**	**Cattle**	**Chicken**	**Food**	**Pig**	**Turkey**	**Other avian**	**Human**	**Chicken**	**Food**	**Pig**	**Turkey**
	1045	290	2	25	8	10	208	119	10	16	5	7	26	95	32	38	22	6	1	63	2	6	11	43
**gyrA S83L**	**223 (21.2)**	17 (5.9)	-	**20 (80.0)**	7 (87.5)	2 (20.0)	22 (10.6)	**23 (19.3)**	6 (60.0)	2 (12.5)	-	1 (14.3)	**10 (38.5)**	8 (8.4)	**25 (78.1)**	**22 (57.9)**	**7 (31.8)**	2 (33.3)	-	1 (1.6)	2 (100)	3 (50.0)	1 (9.1)	**42 (97.7)**
gyrA S83A	5 (0.5)	1 (0.3)	-	-	-	1 (10.0)	2 (1.0)	1 (0.8)	-	-	-	-	-	-	-	-	-	-	-	-	-	-	-	-
gyrA S83V	1 (0.1)	-	-	-	-	-	-	1 (0.8)	-	-	-	-	-	-	-	-	-	-	-	-	-	-	-	-
gyrA D87Y	13 (1.2)	2 (0.7)	-	1 (4.0)	-	-	-	1 (0.8)	-	-	-	-	-	-	1 (3.1)	5 (13.2)	3 (13.6)	-	-	-	-	-	-	-
gyrA D87G	8 (0.8)	1 (0.3)	-	1 (4.0)	-	-	-	1 (0.8)	-	-	-	-	-	2 (2.1)	2 (6.2)	-	1 (4.5)	-	-	-	-	-	-	-
gyrA D87N	8 (0.8)	-	-	-	-	-	-	1 (0.8)	1 (10.0)	-	-	-	-	-	1 (3.1)	3 (7.9)	-	-	-	-	-	2 (33.3)	-	-
gyrA S83L, gyrA D87N	1 (0.1)	-	-	-	-	-	-	-	-	-	-	-	-	-	-	-	1 (4.5)	-	-	-	-	-	-	-
gyrA S83L, parC E84K	1 (0.1)	-	-	1 (4.0)	-	-	-	-	-	-	-	-	-	-	-	-	-	-	-	-	-	-	-	-
gyrA S83L, parC S57T	7 (0.7)	-	-	-	-	-	1 (0.5)	4 (3.4)	-	-	-	-	-	2 (2.1)	-	-	-	-	-	-	-	-	-	-
gyrA S83L, parC S80I	8 (0.8)	1 (0.3)	-	-	-	1 (10.0)	1 (0.5)	1 (0.8)	1 (10.0)	-	1 (20.0)	-	1 (3.8)	-	-	1 (2.6)	-	-	-	-	-	-	-	-
gyrA S83L, parC S80R	5 (0.5)	1 (0.3)	-	1 (4.0)	-	-	1 (0.5)	1 (0.8)	-	-	-	-	-	-	-	1 (2.6)	-	-	-	-	-	-	-	-
gyrA S83V, parC S80I	4 (0.4)	-	-	-	-	-	3 (1.4)	-	-	-	-	-	-	-	-	-	-	-	1 (100)	-	-	-	-	-
gyrA S83L, parE S458A	4 (0.4)	2 (0.7)	-	-	-	-	-	-	-	-	-	1 (14.3)	-	-	-	-	-	-	-	1 (1.6)	-	-	-	-
gyrA D87G, parC S57T	2 (0.2)	-	-	-	-	-	-	-	-	-	-	-	-	-	-	2 (5.3)	-	-	-	-	-	-	-	-
gyrA D87Y, parC S57T	1 (0.1)	-	-	-	-	-	-	-	-	-	-	-	-	-	-	1 (2.6)	-	-	-	-	-	-	-	-
**gyrA S83L, gyrA D87N, parC S80I**	**130 (12.4)**	14 (4.8)	2 (100.0)	-	-	1 (10.0)	**38 (18.3)**	**29 (24.4)**	2 (20.0)	2 (12.5)	-	-	**4 (15.4)**	**31 (32.6)**	1 (3.1)	2 (5.3)	3 (13.6)	-	-	-	-	-	-	1 (2.3)
gyrA S83L, gyrA D87Y, parC S80I	20 (1.9)	-	-	-	-	-	2 (1.0)	14 (11.8)	-	-	-	-	1 (3.8)	2 (2.1)	-	1 (2.6)	-	-	-	-	-	-	-	-

gyrA S83L, gyrA D87G, parC S80I	3 (0.3)	1 (0.3)	-	-	-	-	-	-	-	-	-	-	-	2 (2.1)	-	-	-	-	-	-	-	-	-	-
gyrA S83L, gyrA D87N, parC S80R	5 (0.5)	-	-	-	-	-	1 (0.5)	2 (1.7)	-	-	-	-	-	2 (2.1)	-	-	-	-	-	-	-	-	-	-
gyrA S83L, gyrA D87N, parC S80W	1 (0.1)	-	-	-	-	-	1 (0.5)	-	-	-	-	-	-	-	-	-	-	-	-	-	-	-	-	-
gyrA S83V, gyrA D87Y, parC S80I	1 (0.1)	-	-	-	-	-	-	-	-	-	-	-	-	-	-	-	-	-	-	1 (1.6)	-	-	-	-
gyrA S83L, gyrA D87N, parC E84K	11 (1.1)	-	-	-	-	-	4 (1.9)	4 (3.4)	-	-	-	-	-	2 (2.1)	-	-	-	-	-	1 (1.6)	-	-	-	-
gyrA S83L, gyrA D87N, parC S80I, parC E84G	28 (2.7)	5 (1.7)	-	-	-	-	8 (3.8)	4 (3.4)	-	1 (6.2)	-	-	1 (3.8)	7 (7.4)	1 (3.1)	-	-	1 (16.7)	-	-	-	-	-	-
**gyrA S83L, gyrA D87N, parC S80I, parC E84V**	**296 (28.3)**	**181 (62.4)**	-	-	-	1 (10.0)	**55 (26.4)**	-	-	3 (18.8)	1 (20.0)	1 (14.3)	1 (3.8)	-	-	-	1 (4.5)	-	-	**52 (82.5)**	-	-	-	-
gyrA S83L, gyrA D87N, parC S80I, parC E84A	2 (0.2)	1 (0.3)	-	-	-	-	1 (0.5)	-	-	-	-	-	-	-	-	-	-	-	-	-	-	-	-	-
gyrA S83L, gyrA D87N, parC S80I, parC E84K	1 (0.1)	-	-	-	-	-	-	-	-	-	-	1 (14.3)	-	-	-	-	-	-	-	-	-	-	-	-
gyrA S83L, gyrA D87N, parC S80I, parC E84Q	1 (0.1)	-	-	-	-	-	-	-	-	1 (6.2)	-	-	-	-	-	-	-	-	-	-	-	-	-	-
gyrA S83L, gyrA D87Y, parC S80I, parC E84G	1 (0.1)	-	-	-	-	-	-	1 (0.8)	-	-	-	-	-	-	-	-	-	-	-	-	-	-	-	-
gyrA S83L, gyrA D87Y, parC S80R, parC G78C	1 (0.1)	-	-	-	-	-	-	-	-	-	-	-	-	-	-	-	1 (4.5)	-	-	-	-	-	-	-
gyrA S83L, gyrA D87G, parC S80R, parC G78C	1 (0.1)	-	-	-	-	-	-	1 (0.8)	-	-	-	-	-	-	-	-	-	-	-	-	-	-	-	-
gyrA S83L, gyrA D87N, parC S80I, parC G78C	2 (0.2)	1 (0.3)	-	-	-	-	-	-	-	-	-	-	1 (3.8)	-	-	-	-	-	-	-	-	-	-	-
gyrA S83L, gyrA D87N, parC S80I, parC A56T	21 (2.0)	2 (0.7)	-	-	-	-	4 (1.9)	8 (6.7)	-	-	-	-	1 (3.8)	4 (4.2)	-	-	1 (4.5)	-	-	-	-	1 (16.7)	-	-
gyrA S83L, gyrA D87N, parC S80I, parC S57T	9 (0.9)	1 (0.3)	-	-	-	-	2 (1.0)	-	-	-	-	-	-	5 (5.3)	-	-	-	-	-	1 (1.6)	-	-	-	-
gyrA S83L, gyrA D87N, parC S80I, parC A90V	3 (0.3)	-	-	-	-	-	3 (1.4)	-	-	-	-	-	-	-	-	-	-	-	-	-	-	-	-	-
gyrA S83L, gyrA D87N, parC S80I, parC A108T	1 (0.1)	-	-	-	-	-	-	1 (0.8)	-	-	-	-	-	-	-	-	-	-	-	-	-	-	-	-
gyrA S83L, gyrA D87N, parC S80I, parE L445H	1 (0.1)	1 (0.3)	-	-	-	-	-	-	-	-	-	-	-	-	-	-	-	-	-	-	-	-	-	-
gyrA S83L, gyrA D87N, parC S80I, parE L445I	1 (0.1)	-	-	-	-	-	1 (0.5)	-	-	-	-	-	-	-	-	-	-	-	-	-	-	-	-	-
**gyrA S83L, gyrA D87N, parC S80I, parE S458A**	**183 (17.5)**	**49 (16.9)**	-	1 (4.0)	1 (12.5)	2 (20.0)	**48 (23.1)**	**17 (14.3)**	-	6 (37.5)	3 (60.0)	3 (42.9)	**4 (15.4)**	**26 (27.4)**	1 (3.1)	-	4 (18.2)	3 (50.0)	-	5 (7.9)	-	-	10 (90.9)	-
gyrA S83L, gyrA D87N, parC S80I, parE S458T	9 (0.9)	3 (1.0)	-	-	-	-	2 (1.0)	1 (0.8)	-	-	-	-	1 (3.8)	1 (1.1)	-	-	-	-	-	1 (1.6)	-	-	-	-

gyrA S83L, gyrA D87N, parC S80I, parE S458W	4 (0.4)	2 (0.7)	-	-	-	-	-	1 (0.8)	-	-	-	-	1 (3.8)	-	-	-	-	-	-	-	-	-	-	-
gyrA S83L, gyrA D87N, parC S80R, parE S458A	2 (0.2)	-	-	-	-	-	-	2 (1.7)	-	-	-	-	-	-	-	-	-	-	-	-	-	-	-	-
gyrA S83L, gyrA D87N, parC S80R, parE S458P	5 (0.5)	-	-	-	-	-	4 (1.9)	-	-	-	-	-	-	1 (1.1)	-	-	-	-	-	-	-	-	-	-
gyrA S83L, gyrA D87Y, parC S80I, parE S458A	6 (0.6)	1 (0.3)	-	-	-	1 (10.0)	3 (1.4)	-	-	1 (6.2)	-	-	-	-	-	-	-	-	-	-	-	-	-	-
gyrA S83L, gyrA D87N, parC S80I, parC E84G, parC S57T	1 (0.1)	1 (0.3)	-	-	-	-	-	-	-	-	-	-	-	-	-	-	-	-	-	-	-	-	-	-
gyrA S83L, gyrA D87N, parC S80I, parC E84V, parC S57T	1 (0.1)	1 (0.3)	-	-	-	-	-	-	-	-	-	-	-	-	-	-	-	-	-	-	-	-	-	-
gyrA S83L, gyrA D87N, parC S80I, parC E84G, parC L88Q	2 (0.2)	-	-	-	-	1 (10.0)	1 (0.5)	-	-	-	-	-	-	-	-	-	-	-	-	-	-	-	-	-
gyrA S83L, gyrA D87N, parC S80I, parC E84V, parC G107V	1 (0.1)	1 (0.3)	-	-	-	-	-	-	-	-	-	-	-	-	-	-	-	-	-	-	-	-	-	-

In the table above is listed the six non-synonymous mutations identified in *gyr*A and they encode the following amino acid substitutions: S83A, S83L, S83V, D87G, D87N and D87Y. In *par*C was found fifteen non-synonymous mutations: A56T, S57T, G78C, S80I, S80R, S80W, E84A, E84D, E84G, E84K, E84Q, E84V, L88Q, A90V and G107V. In *par*E, six non-synonymous mutations were identified: L445H, L445I, S458A, S458P, S458T and S458W. Overall 57.5% (1045/1818) of the *E. coli* isolates were resistant to quinolone-fluoroquinolone; 43.3% (787/1818) was fluoroquinolone resistant due to multiple mutations in *gyr*A, *par*C and *par*E and 14.2% (258/1818) was resistant to quinolone due to single mutations in *gyr*A. No mutations in the resistance-determining sites (amino acids 426 and 447) were found in *gyr*B.

Table S13. Proportion of plasmid types in the main ESC-R gene dataset and in the typeable plasmids subset in *E. coli*.

	Main ESC-R gene dataset (n=1,343)		Typeable plasmids subset (n=313)	
**Plasmid type**	**Count**	**%**	**Count**	**%**
IncF	1201	36.58	84	26.84
Col*	733	22.33	5	1.6
IncI1	502	15.29	197	62.94
IncB/O/K/Z	158	4.81	-	-
IncX	152	4.63	4	1.28
IncY	99	3.02	-	-
p0111	96	2.92	-	-
IncHI2	95	2.89	2	0.64
IncN	83	2.53	4	1.28
IncI2	51	1.55	1	0.32
IncHI1	42	1.28	-	-
IncA/C2	40	1.22	7	2.24
IncR	17	0.52	-	-
IncL/M	4	0.12	-	-
pEC4115	4	0.12	-	-
IncP	2	0.06	-	-
IncQ	2	0.06	-	-
pSL483	1	0.03	-	-
repA	-	-	4	1.28
Other**	-	-	5	1.6
TOTAL	3282	100.0	313	100.0

*Col(BS512), Col(KPHS6), Col(MG828), Col(MP18), Col156, Col440I, Col440II, Col8282, ColE10, ColpVC and ColRNAI.

**Cases where two distinct replicons were found in the same plasmid (i.e., ColRNAI+IncI1, IncF+IncI1, IncI1+rep, rep+IncH).

The main plasmid types are highlighted in the table and the dash "-" indicate of the absence of the data.

Table S14. Number of plasmid subtypes for the typeable plasmids subset in *E. coli* and non-*E. coli* species.

ESC-R genes	Plasmid type	pMLST or RST	plasmids in *E. coli* (n=313)	Plasmids in non-*E. coli* (n=36)[&]	non-*E. coli* species
bla$_{CTX-M-1}$	IncF	IncF F59:A-:B-	4	-	
	IncF	IncF*	1	-	
	IncI1	IncI1 pST3	84	-	
	IncI1	IncI1 pST7	8	-	
	IncI1	IncI1 pST26	1	-	
	IncI1	IncI1 pST312	1	-	
	IncI1	IncI1 pST35	1	-	
	IncI1	IncI1 pST63, IncF F56:A-:B-	1	-	
	IncI1	IncI1*	30	-	
	IncN	IncN pST1	4	13	*S. enterica*
	IncH	IncH*	1	-	
bla$_{CTX-M-15}$	IncF	IncF F2:A-:B-	5	-	
	IncF	IncF F1:A-:B-	1	-	
	IncF	IncF F2:A(1, 6):B-	1	-	
	IncF	IncF F22:A-:B-	1	-	
	IncF	IncF F46:A-:B-	1	-	
	IncI1	IncI1 pST16	1	-	
	IncI1	IncI1*	6	-	
	IncI2	IncI2*	1	-	
	IncX	IncX1**	1	-	
	ColRNAI	ColRNAI**	2	1	*K. pneumoniae*
	rep	rep**	3	-	
	IncR	IncR**	-	2	*K. pneumoniae*
bla$_{CMY-2}$	IncF	IncF F-:A-:B1	3	-	
	IncF	IncF F34:A-:B-	1	-	
	IncF	IncF F6:A-:B-	1	-	
	IncF	IncF*	30	-	
	IncI1	IncI1 pST12	6	7	*S. enterica*
	IncI1	IncI1 pST2	7	-	
	IncI1	IncI1 pST20	3	-	
	IncI1	IncI1 pST26	1	-	
	IncI1	IncI1 pST43	1	-	
	IncI1	IncI1*	24	-	
	IncA/C2	IncA/C2 pST3	7	-	
	IncH	IncH*	1	-	
	IncX	IncX1**	1	-	
	IncX	IncX2**	1	-	
	ColRNAI	ColRNAI**	1	-	
bla$_{CTX-M-14}$	IncF	IncF F2:A-:B-	17	-	
	IncF	IncF F-:A(2, 6):B-	1	-	
	IncF	IncF F35:A-:B-	1	-	
	IncF	IncF*	2	1	*K. pneumoniae*
	IncI1	IncI1 pST166	1	-	
	IncI1	IncI1*	18	-	
	ColRNAI	ColRNAI**	-	1	*K. pneumoniae*
bla$_{SHV-12}$	IncF	IncF F4:A-:B-	5	-	
	IncF	IncF F-:A-:B73	1	-	
	IncF	IncF*	8	-	
	IncI1	IncI1 pST26	1	4	*S. enterica*
	IncI1	IncI1*	6	7	*S. enterica*
	IncH	IncH*	1	-	
	IncX	IncX3**	1	-	

ColRNAI	ColRNAI**	2	-
rep	rep**	1	-

[&]In our dataset of 108 non-*E. coli*, 98 genomes carry the main ESC-R genes, of which were recovered 36 typeable plasmids.
*Plasmids where the sequences for subtyping were not found in the same contig.
**There is no pMLST scheme for IncX, IncR and ColRNAI plasmids.
Plasmid subtypes found in *E. coli* and non-*E. coli* are highlighted in the table.

SUPPLEMENTAL REFERENCES

1. Government of Canada. *Canadian integrated program for antimicrobial resistance surveillance (CIPARS) 2018: Design and methods*. https://www.canada.ca/content/dam/phac-aspc/documents/services/surveillance/canadian-integrated-program-antimicrobial-resistance-surveillance-cipars/cipars-reports/2018-annual-report-design-methods/2018-annual-report-design-methods.pdf (2020).
2. Chalmers, G. *et al.* Determinants of virulence and of resistance to ceftiofur, gentamicin, and spectinomycin in clinical *Escherichia coli* from broiler chickens in Québec, Canada. *Vet. Microbiol.* **203**, 149–157 (2017).
3. Cormier, A. C. *et al.* Presence and diversity of extended-spectrum cephalosporin resistance among *Escherichia coli* from urban wastewater and feedlot cattle in Alberta, Canada. *Microb. Drug Resist.* **26**, 300–309 (2020).
4. Zhang, P. L. C. *et al.* Prevalence and mechanisms of extended-spectrum cephalosporin resistance in clinical and fecal *Enterobacteriaceae* isolates from dogs in Ontario, Canada. *Vet. Microbiol.* **213**, 82–88 (2018).
5. Wick, R. R., Judd, L. M., Gorrie, C. L. & Holt, K. E. Completing bacterial genome assemblies with multiplex MinION sequencing. *Microb. Genomics* **3**, (2017).
6. Wick, R. R., Judd, L. M., Gorrie, C. L. & Holt, K. E. Unicycler: Resolving bacterial genome assemblies from short and long sequencing reads. *PLOS Comput. Biol.* **13**, e1005595 (2017).
7. Walker, B. J. *et al.* Pilon: an integrated tool for comprehensive microbial variant detection and genome assembly improvement. *PLoS One* **9**, e112963 (2014).
8. Zankari, E. *et al.* Identification of acquired antimicrobial resistance genes. *J. Antimicrob. Chemother.* **67**, 2640–2644 (2012).
9. Seemann, T. Prokka: rapid prokaryotic genome annotation. *Bioinformatics* **30**, 2068–9 (2014).
10. Page, A. J. *et al.* Roary: rapid large-scale prokaryote pan genome analysis. *Bioinformatics* **31**, 3691–3693 (2015).
11. Abudahab, K. *et al.* PANINI: pangenome neighbour identification for bacterial populations. *Microb. Genomics* **5**, (2019).
12. Pietsch, M. *et al.* Whole genome analyses of CMY-2-producing *Escherichia coli* isolates from humans, animals and food in Germany. *BMC Genomics* **19**, 601 (2018).
13. Kallonen, T. *et al.* Systematic longitudinal survey of invasive *Escherichia coli* in England demonstrates a stable population structure only transiently disturbed by the emergence of ST131. *Genome Res.* **27**, 1437–1449 (2017).
14. Ludden, C. *et al.* One Health genomic surveillance of *Escherichia coli* demonstrates distinct lineages and mobile genetic elements in isolates from humans versus livestock. *MBio* **10**, (2019).

15. Kassambara, A. Package ‘rstatix’: pipe-friendly framework for basic statistical tests.
