# Supplementary material for: Dynamics of extended-spectrum cephalosporin resistance genes in Escherichia coli from Europe and North America
Source: Nat Commun. 2022 Dec 12;13:7490. doi: 10.1038/s41467-022-34970-7 (PMC9744880; doi:10.1038/s41467-022-34970-7)
Supplement: Supplementary file 2 — Reporting Summary [file 41467_2022_34970_MOESM2_ESM.pdf]

nature portfolio

Corresponding author(s): Alison E. Mather

Last updated by author(s): Oct 27, 2022

Reporting Summary

Nature Portfolio wishes to improve the reproducibility of the work that we publish. This form provides structure for consistency and transparency in reporting. For further information on Nature Portfolio policies, see our Editorial Policies and the Editorial Policy Checklist.

Statistics

For all statistical analyses, confirm that the following items are present in the figure legend, table legend, main text, or Methods section.

n/a	Confirmed	
☐	☒	The exact sample size (*n*) for each experimental group/condition, given as a discrete number and unit of measurement
☒	☐	A statement on whether measurements were taken from distinct samples or whether the same sample was measured repeatedly
☐	☒	The statistical test(s) used AND whether they are one- or two-sided<br>*Only common tests should be described solely by name; describe more complex techniques in the Methods section.*
☒	☐	A description of all covariates tested
☐	☒	A description of any assumptions or corrections, such as tests of normality and adjustment for multiple comparisons
☐	☒	A full description of the statistical parameters including central tendency (e.g. means) or other basic estimates (e.g. regression coefficient) AND variation (e.g. standard deviation) or associated estimates of uncertainty (e.g. confidence intervals)
☐	☒	For null hypothesis testing, the test statistic (e.g. *F*, *t*, *r*) with confidence intervals, effect sizes, degrees of freedom and *P* value noted<br>*Give P values as exact values whenever suitable.*
☒	☐	For Bayesian analysis, information on the choice of priors and Markov chain Monte Carlo settings
☒	☐	For hierarchical and complex designs, identification of the appropriate level for tests and full reporting of outcomes
☒	☐	Estimates of effect sizes (e.g. Cohen's *d*, Pearson's *r*), indicating how they were calculated

Our web collection on statistics for biologists contains articles on many of the points above.

Software and code

Policy information about availability of computer code

Data collection

No software was used for data collection

Data analysis

- Adapters and poor quality bases in short-reads data were removed with Trimmomatic v0.33
- Draft genomes were assembled using Spades v3.11.1
- Summary statistics of the assembly was performed with Quast v4.5
- Quality of the genomes was assessed using CheckM v1.1.2
- Species detection was performed using Centrifuge v1.0.3-beta and Kleborate v2.0.4
- Average nucleotide identity (ANI) was estimated between NCBI genome references and the genomes with discrepancies between Centrifuge and Kleborate species assignation. The ANI was estimated using FastANI v1.32
- Depth coverage was obtained using BWA v0.7.17, Samtools v1.9 and Bcftools v1.8
- Sequence type was identified using multilocus sequence typing (MLST) scheme applied in ARIBA v2.13.5
- Phylogroups were obtained with ClermonTyping tool v1.4.1.
- Short-reads for main STs (ST131, ST10 and ST117) were mapped against reference genomes using Snippy v4.3.6
- Pairwise SNP distances were obtained using snp-dists v0.7.0
- AMR genes and plasmid replicons were identified using the ResFinder and PlasmidFinder databases with ARIBA v2.13.5 and Abricate v0.9.8.
- Mutations in gyrase genes and topoisomerase IV genes were obtained using ARIBA v2.13.5
- Prediction of contigs as plasmid or chromosomal origin was performed using MOB-suite v2.0 and RFPlasmid.
- Plasmid subtyping was performed using the Blast+ v2.9.0 and the plasmid MLST database (https://bitbucket.org/genomicepidemiology/pmlst_db/)
- Diversity analysis was performed using R packages vegan v2.5-6 and iNEXT v2.0.20
- Draft assemblies were annotated using Prokka v.1.13

- Pangenome was obtained with Roary v3.12.0
- SNPs were extracted from the core gene alignment with snp-sites v2.5.1
- Phylogenetic trees were constructed using IQ-tree v1.6.11
- Tree and metadata were plotted with ggtree v2.0.2
- Statistical analysis (Fisher's Exact Test, Kruskal-Wallis and Mann-Whitney U) were performed using rstatix's v0.5.0
- Plasmidome and pangenome network were analyzed using PANINI web tool (https://panini.cgps.group/)
- Base-calling of the fast5 files and demultiplexing of the Nanopore reads were performed with Guppy Basecaller v4.5.4 and Guppy Barcoder v4.5.4. These tools are mentioned in 'Section 2' of the 'Supplemental Methods' available in the supplementary material file.
- Long-reads were trimmed and filtered using Porechop v0.2.4 and Filtlong v0.2.0, respectively. These tools are mentioned in 'Section 2' of the 'Supplemental Methods' available in the supplementary material file.
- Hybrid assemblies were obtained using Unicycler v0.4.8 and polished with Pilon v1.22. These tools are mentioned in 'Section 2' of the 'Supplemental Methods' available in the supplementary material file.

For manuscripts utilizing custom algorithms or software that are central to the research but not yet described in published literature, software must be made available to editors and reviewers. We strongly encourage code deposition in a community repository (e.g. GitHub). See the Nature Portfolio guidelines for submitting code & software for further information.

Data

Policy information about availability of data

All manuscripts must include a data availability statement. This statement should provide the following information, where applicable:

- Accession codes, unique identifiers, or web links for publicly available datasets
- A description of any restrictions on data availability
- For clinical datasets or third party data, please ensure that the statement adheres to our policy

Illumina sequence read data generated in this study have been deposited in the European Nucleotide Archive (ENA) database and are publicly available under BioProject PRJEB38235 [https://www.ebi.ac.uk/ena/browser/view/PRJEB38235], PRJEB42322 [https://www.ebi.ac.uk/ena/browser/view/PRJEB42322], PRJNA523640 [https://www.ebi.ac.uk/ena/browser/view/PRJNA523640], PRJNA556083 [https://www.ebi.ac.uk/ena/browser/view/PRJNA556083], PRJNA740259 [https://www.ebi.ac.uk/ena/browser/view/PRJNA740259] and PRJEB50837 [https://www.ebi.ac.uk/ena/browser/view/PRJEB50837] and individual accession numbers are available in 'Source Data' file. Long-read data for the subset of 20 genomes are deposited in the ENA under BioProject PRJEB54884 [https://www.ebi.ac.uk/ena/browser/view/PRJEB54884]; the accession numbers for each genome are placed in the 'Source Data' file. In the 'Source Data' file, accession numbers and hyperlinks for each genome analysed in this study, alongside with the isolate collection metadata, are included.

Human research participants

Policy information about studies involving human research participants and Sex and Gender in Research.

Reporting on sex and gender	N/A
Population characteristics	N/A
Recruitment	N/A
Ethics oversight	N/A

Note that full information on the approval of the study protocol must also be provided in the manuscript.

Field-specific reporting

Please select the one below that is the best fit for your research. If you are not sure, read the appropriate sections before making your selection.

☐ Life sciences ☐ Behavioural & social sciences ☒ Ecological, evolutionary & environmental sciences

For a reference copy of the document with all sections, see nature.com/documents/nr-reporting-summary-flat.pdf

Ecological, evolutionary & environmental sciences study design

All studies must disclose on these points even when the disclosure is negative.

Study description	This study assessed the distribution and dynamics of extended-spectrum cephalosporin resistance (ESC-R) genes in a large and comprehensive collection of Enterobacterales from diverse sources and countries. This was investigated using whole genome sequences (WGS).
Research sample	The research sample comprises bacterial genome data originally isolated from humans, animals, food and the environment. The research samples used here were from researchers/co- authors of this study based at national reference laboratories in Canada, France and Germany, countries with extensive and long- term bacterial collections (from human, animal, environmental and food/ meat sources) with associated epidemiological metadata. The bacterial collection used in this study is predominantly comprised of Escherichia coli resistant to extended spectrum cephalosporins. Additionally, we included publicly available E. coli genomes from

	other studies (Pietsch et al 2018 with isolates from Germany [https://doi.org/10.1186/s12864-018-4976-3], Kallonen et al 2017 with isolates from the UK [http://www.genome.org/cgi/doi/10.1101/gr.216606.116], Ludden et al 2019 with isolates from the UK [https://doi.org/10.1128/mBio.02693-18]) for context.
Sampling strategy	The main aim of our study was to examine broader trends in ESC-R E. coli (n=1,524) within the primary three countries (France, Canada and Germany) across One Health compartments. Therefore, our strategy for sample selection was as follows: the majority (84%) of isolates were selected to include representatives from different ecological compartments (human, animal, food/ meat) of ESC-R E. coli isolated from 2008 to 2016. These isolates were randomly selected from available culture collections across three countries: France, Germany and Canada. The remaining 16% of isolates were selected to provide context, namely: 1) ESC-R E. coli isolates obtained prior to 2008 or after 2016 (4%); 2) ESC-R isolates of bacterial species other than E. coli from 2008 to 2016 (5.6%); and 3) ESC-susceptible Enterobacterales (6.5%). These genomes have extensive associated metadata, allowing us to investigate the dynamics of ESC-R across time, host species and countries.<br><br>In addition, genomes from Germany (n=158) and the UK (n=248) from previously published studies were included; these genomes were selected following a similar approach of sample selection as described above, where all genomes available between 2008 and 2016 were included and a small number of genomes collected prior to 2008 and/or genomes of ESC-susceptible isolates were selected.
Data collection	An extensive collection of 1,930 Enterobacterales genomes was compiled. These genomes were from Canada (n=718), France (n=607), Germany (n=357) and the UK (n=248). They belong to the following bacterial species: E. coli (n=1818), Klebsiella pneumoniae (n=46), Salmonella enterica (n=44) and other enteric bacteria species (n=22). Of these, 1,524 isolates were from this study and 406 genome sequences from previously published studies. The genomes derived mainly from human (n=817), animal (n=947) and food (n=150) sources. Epidemiological metadata are available for our large genome collection. Moreover, long-read data were generated for a subset of 20 genomes.<br><br>The accession numbers for the genome data (n=1,930) and respective metadata are publicly available and can be found in the 'Source Data' file (Excel document).
Timing and spatial scale	The samples were collected between 2003 and 2017 from Canada, Germany, France and the UK, with the majority from 2008 to 2016.
Data exclusions	Genomes were excluded if they have:<br>- Depth coverage below 10X<br>- High number of contigs (>900 contigs)<br>- Genome completeness below 95.0% and/or contamination in the assembly with >15.0%
Reproducibility	To ensure reproducibility we have submitted the genome data (short- and long-reads data) in the ENA database (https://www.ebi.ac.uk/ena/browser/home) and are publicly available. Furthermore, we have reported in the 'Source Data' file the accession number for each genome along with the metadata (source, country and year), assembly summary (number of contigs, largest contig and total length) and genetic data (bacteria species, sequence type (ST), ESC-R genes, other acquired AMR genes and plasmid replicons). All bioinformatics tools used in this study were described in the manuscript and supplemental methods (section 2).
Randomization	These isolates were randomly selected from available large bacterial collections across three countries: France, Germany and Canada. Moreover, the ESC-R isolates were selected on their resistance to an ESC drug, not on their genotype (i.e., specific ESCR gene). Therefore, the relative frequency and occurrence of these genes are representative of what was circulating in the general population during the time frame and can be statistically compared.
Blinding	Blinding was not relevant for this study because it is not a clinical trial. This is a retrospective and descriptive study where we examine the distribution and dynamics over time of ESC-R genes among compartments (country + source) in ESC-R E. coli. Furthermore, all ESC-R isolates included in this study were selected on their resistance phenotype to an ESC drug, not on their genotype (i.e., specific ESC-R gene).

Did the study involve field work? ☐ Yes ☒ No

Reporting for specific materials, systems and methods

We require information from authors about some types of materials, experimental systems and methods used in many studies. Here, indicate whether each material, system or method listed is relevant to your study. If you are not sure if a list item applies to your research, read the appropriate section before selecting a response.

Materials & experimental systems

n/a	Involved in the study
☒	☐ Antibodies
☒	☐ Eukaryotic cell lines
☒	☐ Palaeontology and archaeology
☒	☐ Animals and other organisms
☒	☐ Clinical data
☒	☐ Dual use research of concern

Methods

n/a	Involved in the study
☒	☐ ChIP-seq
☒	☐ Flow cytometry
☒	☐ MRI-based neuroimaging
